# Supplementary material for: Ancient genomes from the last three millennia support multiple human dispersals into Wallacea
Source: Nat Ecol Evol. 2022 Jun 9;6(7):1024–34. doi: 10.1038/s41559-022-01775-2 (PMC9262713; doi:10.1038/s41559-022-01775-2)
Supplement: Supplementary file 1 — Supplementary Figs. 1–11. [file 41559_2022_1775_MOESM1_ESM.pdf]

nature portfolio

https://doi.org/10.1038/s41559-022-01775-2

Supplementary information

Ancient genomes from the last three millennia support multiple human dispersals into Wallacea

In the format provided by the authors and unedited

Supplementary Information

1. Ethics statement

The human remains analyzed in this study are considered the cultural heritage of the originating countries. Appropriate research permits and export approvals were obtained from Indonesia to conduct the current research. For the skeletal material from Tanjung Pinang and Uattamdi, the research was undertaken as part of a collaborative project between Pusat Penelitian Arkeologi Nasional (Jakarta) and the Australian National University, under Lembaga Ilmu Pengetahuan Indonesia Research Permits 6939/S.K./1990, 307/I/KS/1994, and 10290/V3/KS/1995. For the skeletal material from Aru Manara, the research was undertaken as part of a collaborative project between Pusat Penelitian Arkeologi Nasional (Jakarta) and the Tokai University, under Kementarian Riset dan Teknologi Research Permits 0291/SIP/FRP/VIII/2011 and Pusat Penelitian Arkeologi Nasional export permit UM.001/2595/PAN/KPK/IX/2012, 2634/H5/TU/2017. For the skeletal material from Topogaro, the research was undertaken as part of a collaborative project between Pusat Penelitian Arkeologi Nasional (Jakarta) and the Tokai University, under Kementarian Riset dan Teknologi Research Permits 40/EXT/SIP/FRP/SM/VII/2015 and 194/SIP/FRP/E5/Dit.KI/VII/2017 and Pusat Penelitian Arkeologi Nasional export permit 2634/H5/TU/2017. For the skeletal material from Liang Bua, Liang Toge and Komodo, the research was undertaken as part of a collaborative project between Universitas Airlangga (Surabaya, Indonesia) and the Max Planck Institute for the Science of Human History (Jena, Germany) under the Kementerian Ristekdikti Research Permit 303/SIP/FRP/E5/Dit.KI/IX/2017 and Pusat Penelitian Arkeologi Nasional export permit 11404H5/TU/2017. Research of the human remains from Jareng Bori were carried out as part of a collaboration between Australian National University and Universitas Gaja Madja under the Kementerian Ristekdikti Research Permit 1209/FRP/E5/Dit.KI/VI/2016.

2. Radiocarbon dating

Nine new radiocarbon dates for this study (Supplementary Table 12) were produced at the Curt-Engelhorn-Zentrum Archäometrie gGmbH in Mannheim, Germany. There, collagen from bone and dentin was extracted using a modified Longin method[1] and long molecules removed with ultrafiltration before freeze-drying the product[2]. After pretreatment, organic samples were combusted in an elemental analyser (Microcube, Elementar, Hanau, Germany). Following catalytic reduction to graphite, the ^{14}C content was measured with an AMS-System type MICADAS[3]. The Liang Bua individual was dated at the Radiocarbon Dating Laboratory, University of Waikato. The bone was decalcified and then gelatinized before ultrafiltration. Pressed graphite was analysed at the Keck Radiocarbon Dating Laboratory, the University of California on a NEC 0.5MV 1.5SDH-1 AMS system coupled with an in-house modified ion source[4]. Reported $\delta^{15}N$, $\delta^{13}C$ values for dietary calibration correction were measured by Isotope Ratio Mass Spectrometry (IRMS) at Iso-trace Research Department of Chemistry, the University of Otago on a Carlo Erba NA 1500 elemental analyser (EA), coupled with either a Europa Scientific ‘20/20 Hydra’ or a Thermo Finnigan Delta Plus Advantage. The Uattamdi individual was dated in the University of Oxford's Radiocarbon Accelerator Unit (ORAU). The bone powder was given a solvent wash due to possible glue present. This comprised a sequence of extraction in acetone, methanol and chloroform. This was then followed by routine ABA pretreatment and ultrafiltration[5]. The ultrafiltered collagen was combusted using an automated carbon and nitrogen elemental analyzer (Carlo Erba EA1108) coupled with a continuous-flow isotope monitoring mass spectrometer (Europa Geo 20/20). $\delta^{15}N$ and $\delta^{13}C$ values, % carbon values, as well as C:N atomic ratios were obtained. We used VPDB as the standard for determining $\delta^{13}C$ values and AIR for the $\delta^{15}N$ equivalent. AMS radiocarbon measurement was undertaken using the MiCaDaS accelerator[6, 7, 8]. The Tanjung Pinang individual was dated in the Australian National University. The sample was crushed and treated with Acetic Acid then rinsed to separate the bone apatite.

The bone protein samples were corrected for marine reservoir effect using the Mixed_Curves approach in OxCal 4.4 with Marine20 and INTCAL20 curves[9, 10] assuming a regional ΔR average for the Indonesian islands of -173±75 ^{14}C yrs obtained from the Marine Reservoir Corrections Database (http://calib.org/marine/). The percent contribution of

the marine curve was determined based on a linear interpolation of the measured $\delta^{13}C$ value between assumed endpoints of -21 and -12‰ (terrestrial and marine). Bone carbonate $\delta^{13}C$ (e.g., ANU-8439) is a function of the bulk diet and is offset by around +12‰ to +14‰[11]; consequently, the reported value represents a terrestrial diet and has been calibrated accordingly.

The radiocarbon dates and quality collagen indicators (collagen yields, C/N ratios, %C and %N) are reported in Supplementary Table 12.

3. Archaeological information

Aru Manara. Aru Manara is a limestone cave located about 275 m inland from the southeastern coast of Morotai island, North Maluku Islands, Eastern Indonesia, excavated in 2011. The stratigraphy of the site comprises four deep sediment layers 2 m thick that terminated in the bedrock. The individuals studied here were excavated from Layer 3. The excavations yielded a large number of fragmented human bone (NISP=57,273) and teeth (n=1550), as well as early Metal Age sherds dated to between 2300 and 1800 cal BP, (mainly from Layer 3)[12-14]. The other sherds found at the site (n=504) included 32 red-slipped and 79 decorated specimens. One type of pottery decoration, with linear and curvilinear motifs, are similar to those in contemporary Moluccan assemblages at Tanjung Pinang on Morotai, also analyzed in this study[15]. However, the occurrence of anthropo- and zoomorphic decorations are unique to this site within the Northern Moluccas. The existence of pottery with applied-relief decorations, including a human face and lizard, suggest the development of maritime networks with East Indonesia and Near Oceania from 2700 to 2000 years BP, but is also linked with ceramics from Lapita associated burials in Remote Oceania[16]. The use of large burial jars and box typed jars may indicate possible human interaction between the Northern Moluccas and the Philippines[17]. The skeletal remains from Aru Manara are curated by the Pusat Penelitian Arkeologi Nasional (Jakarta, Indonesia).

Topogaro 1. Topogaro 1 is a cave that is part of a larger system of three caves (Topogaro 1-3) and rockshelters (Topogaro 4-7), located about 3.5 km inland from the eastern coast of Central Sulawesi in Morowali District. Topogaro 1 contains more than 30 broken coffins with human skeletal remains and surface finds, including fragments of prehistoric pottery, Chinese and European ceramics, achert flakes (including finely retouched tools), and shell. The presence of a number of flakes and shellfish clearly indicate that the cave had been used as a prehistoric habitation or tool production site, while wooden coffins and the variety of Chinese and European ceramics show the site had been used as a burial site more recently, between 300 and 100 years BP, based on AMS dates acquired from the coffin itself and some associated human teeth. The skeletal remains from Topogaro are curated by the Pusat Penelitian Arkeologi Nasional (Jakarta, Indonesia).

Gua Uattamdi. Gua Uattamdi is a limestone rock shelter situated on Kayoa Island in the northern Moluccas (0° 127°20’E). This site has yielded skull fragments associated with jar burial, incised pottery, glass beads and metal fragments, with one individual analysed in this report directly dated to 1820 - 1530 cal BP. The oldest material culture from Uattamdi contained red-slipped Neolithic pottery, dated to c.3300–2500 BP, with connections with the Philippines and eastern Taiwan, as well as other Neolithic sites in Wallacea. This Neolithic layer contained no human remains. The younger incised, impressed and appliqué pottery associated with the analysed skull is paralleled in sites on Morotai, including the two sites of Aru Manara and Tanjung Pinang that are also analysed in this study (ref[15], ch.7). This style of pottery dates to c.2500 BP and later. The skeletal remains from Gua Uattamdi are curated by the Pusat Penelitian Arkeologi Nasional (Jakarta, Indonesia).

Tanjung Pinang. Tanjung Pinang is a rock shelter situated on Morotai Island (2°05’N, 128°40’E). There is a direct date for one of the four individuals included in this study (TanjungPinang1) of 2690 - 1600 cal BP. The date was produced from bone apatite in 1993, therefore most likely showing a minimum date (Rachel Wood, ANU Radiocarbon Laboratory, pers. Comm.), and it exhibits a large calibration range. However, the incised decoration of the pottery found at this site is similar to the Metal Age pottery in Gua Uattamdi (ref[15], ch7), supporting a date younger than the Neolithic red-slipped pottery in Uattamdi. Nevertheless, the associated pottery and the apatite

analysis allow a possibility that the Tanjung Pinang sample could be as old as 2500 BP. A morphological analysis of the Tanjung Pinang skulls showed Papuan features[18]. The skeletal remains from Tanjung Pinang are curated by the Pusat Penelitian Arkeologi Nasional (Jakarta, Indonesia).

Liang Bua. Liang Bua is best known as the cave where *Homo floresiensis* was discovered[19, 20]. However, Liang Bua also contains archaeological deposits dating to the Neolithic and Proto-Metallic periods. These later deposits were first excavated in 1965 by T.H. Verhoeven who discovered six skeletons[21, 22]. Five skulls from that excavation are curated at Universitas Airlangga and loose petrous bones from Burials 1 and 2 were sampled for the current study. It was later found that both petrous fragments belonged to the crania of Burial 2. The individual included[23] in this study was directly dated to 2588 ± 23 BP (Wk-51763). In both the earlier and later excavations, the burials were found associated with Neolithic and Proto-Metallic material culture and domestic fauna, such as plain and decorated pottery (*periuk* [jars], *kendi*[pitcher/ewer], *buli buli* [little jars], and *tutup* [lid]), flaked adzes, bone tools, pig tusks and a bronze axe[19, 22, 24]. Bioarchaeological analysis confirmed that 3/3 (100%) of the Liang Bua individuals curated at Universitas Airlangga who had preserved anterior maxillary teeth (including burial 2) had undergone ritual tooth ablation, a practice thought to have been introduced by the Neolithic Austronesian settlers to East Nusa Tenggara[23, 25-27].

Liang Toge. One individual was analysed in this study from the cave site of Liang Toge on Flores Island, located near Warukia, 1 km south of Lepa, in the Manggarai district of Flores[28]. Excavated by T.H. Verhoeven in the mid 20th century, there is little published information about the human remains from Liang Toge, which are currently curated at Universitas Airlangga, Surabaya. Visual inspection of the Liang Toge skeletal assemblage suggests that these remains may have been (or become after excavation) commingled because there were mixed cranial and post-cranial elements from multiple individuals in the collection and no apparent burial numbers. The petrous bone analyzed for this study was dated to 800 – 720 cal BP (MAMS 40606), in addition to two ribs which provided dates of 1066 – 973 cal BP (MAMS 35084) and 911 – 786 cal BP (MAMS 35085).

Komodo. The individual (Komodo I) analysed from Komodo Island, West Manggarai Regency, East Nusa Tenggara (S 08° 32’ 35.9988” E 119° 29’ 21.9876”) was one of two burials found during excavations carried out by Walter Auffenberg (University of Florida, USA) and Putra Sastrawan (Universitas Udayana, Indonesia) in 1969-1970. During their survey for Komodo dragons, Auffenberg and Putra Sastrawan identified 18 archaeological sites across Komodo and the two burials were found at the hill site of Ntodo Leseh. The crania were removed from the primary flexed (or squatting) burials (both with their heads facing west) and they were designated Komodo I and Komodo II[29, 30]. Komodo I was buried 16 cm below the surface and had a circle of flat stones marking the grave. Komodo II was buried 19 cm below the surface and had a triangle of flat stones marking the grave. From comparisons of burial type with other prehistoric interments on Flores and East Java, in addition to material culture (stone flakes and blades and Neolithic hand axes) found at the other sites on Komodo, ref. [23] proposed that the Komodo burials were between 3000-5000 years old. However, recent direct dating of the petrous bone from Komodo I provided a much more modern date of 570 - 240 cal BP (MAMS 40602) indicating that this individual lived during the period when East Nusa Tenggara was under the rule of the Majapahit Empire[31]. The skeletal remains from Jareng Bori are curated at Universitas Airlangga (Surabaya, Indonesia).

Jareng Bori. The site of Jareng Bori, Pantar island, East Nusa Tenggara (S 08° 15’51.7 E 124°17’55.4) was excavated in 2016 as part of a joint research project between researchers from the Australian National University (Australia) and Universitas Gaja Madja (Indonesia). The small rock shelter (40 m2 living floor) was located beside a large boulder at the base of a cliff on a coastal beach flat approximately 120 m from the current shoreline. A 1 x 1 m test pit (test pit A) was excavated and seven stratigraphic layers were identified at the site. The period of site use was dated between the Metal Age, *ca* 1800 BP, and the late Historic Period. A site report detailing the full excavation and results of the dating, faunal and material culture analyses is available[32]. At Jareng Bori, a poorly preserved human burial (analyzed for the current study) was found cut into the upper layers with a skeleton in a flexed position in the south area of the test pit. Only the upper half (thorax, upper limbs and skull) of the skeleton was excavated and the remainder was left

in situ because it extended into the southern baulk of the test pit. The skeletal remains were analysed at Australian National University and a full bioarchaeology report is forthcoming. Of the 21 charcoal dates analysed from the site, four were associated with the burial (ANU 53127, ANU 53130, ANU 53129, and ANU136) and ranged between 0-429 cal BP[32]. The direct date of the petrous bone for this burial provided results of 530 – 490 cal BP (MAMS 40607), indicating that this individual lived during the rule of the Majahapit Empire in the region, directly before the first Portuguese contact with the area at the beginning of the 16th century AD[31]. Tooth modifications in the form of labial filing of the maxillary incisors was found on the dentition of the Jareng Bori individual. This type of tooth modification has been observed on skeletons dating to a similar time period in Java, Bali, Sumba and Flores. Tooth filing has been suggested as a cultural tradition that is a unique characteristic of later inhabitants of the region[25, 27, 33], whereas tooth ablation (in the form of the removal of the maxillary lateral incisors and canines) has been interpreted as an earlier Neolithic/Austronesian ritual practiced across East Nusa Tenggara *ca.* 3000 - 2000 BP[25-27]. The skeletal remains from Jareng Bori are curated at Universitas Airlangga (Surabaya, Indonesia).

References

1 Longin, R. New method of collagen extraction for radiocarbon dating. *Nature* **230**, 241-242 (1971).

2 Higham, T. F., Jacobi, R. M. & Ramsey, C. B. AMS radiocarbon dating of ancient bone using ultrafiltration. *Radiocarbon* **48**, 179-195 (2006).

3 Kromer, B., Lindauer, S., Synal, H.-A., Wacker, L. MAMS - a new AMS facility at the Curt-Engelhorn-Centre for achaeometry, Mannheim, Germany. *Nucl Instrum Methods Phys Res,* Sect. B **294**, 11–13 (2013).

4 Beverly, R.K. *et al*. The Keck Carbon Cycle AMS Laboratory, University of California, Irvine: Status Report. *Radiocarbon*, **52** (2-3), 301–309 (2010)

5 Brock, F., Higham, T., Ditchfield, P. & Ramsey, C. B. Current pretreatment methods for AMS radiocarbon dating at the Oxford Radiocarbon Accelerator Unit (ORAU). *Radiocarbon* **52**, 103-112 (2010).

6 Stuiver, M. & Polach, H. A. Discussion reporting of 14 C data. *Radiocarbon* **19**, 355-363 (1977).

7 Dee, M. & Ramsey, C. B. Refinement of graphite target production at ORAU. *Nucl Instrum Methods Phys Res B* **172**, 449-453 (2000).

8 Ramsey, C. B., Higham, T. & Leach, P. Towards high-precision AMS: progress and limitations. *Radiocarbon* **46**, 17-24 (2004).

9 Reimer, P. J. *et al.* The IntCal20 Northern Hemisphere radiocarbon age calibration curve (0–55 cal kBP). *Radiocarbon* **62**, 725-757 (2020).

10 Heaton, T. J. *et al.* Marine20—the marine radiocarbon age calibration curve (0–55,000 cal BP). *Radiocarbon* **62**, 779-820 (2020).

11 Petchey, F., Spriggs, M., Bedford, S., & Valentin, F. The chronology of occupation at Teouma, Vanuatu: Use of a modified chronometric hygiene protocol and Bayesian modelling to evaluate midden remains. *Journal of Archaeological Science: Reports* **4**, 95-105 (2015).

12 Ono, R. *et al.* Development of regional maritime networks during the Early Metal Age in northern Maluku Islands: A view from excavated glass ornaments and pottery variation. *The J Island Coast Archaeol* **13**, 90-108 (2018).

13 Ono, R. *et al.* Development of pottery making tradition and maritime networks during the Early Metal Ages in Northern Maluku Islands. *AMERTA* 35 **2**, 109-122 (2017).

14 Ono, R. *et al.* Early Metal Age interactions in Island Southeast Asia and Oceania: jar burials from Aru Manara, northern Moluccas. *Antiquity* **92**, 1023-1039 (2018).

15 Bellwood, P. *The Spice Islands in Prehistory: Archaeology in the Northern Moluccas, Indonesia*. (ANU Press, 2019).

16 Spriggs, M. Archaeological and Ethnoarchaeological Research (1990).

17 Ono, R, Oktaviana AA, Sriwigati, Aziz N. In *The Archaeology of Island Colonization* (ed Matthew F. Napolitano, Robert J. DiNapoli), 293-326 (University Press of Florida, 2021).

18 Bulbeck, D. in *The Spice Islands in Prehistory Archaeology in the Northern Moluccas, Indonesia* (JSTOR, 2019).
19 Morwood, M. J. *et al.* Archaeology and age of a new hominin from Flores in eastern Indonesia. *Nature* **431**, 1087-1091 (2004).
20 Sutikna, T. *et al.* The spatio-temporal distribution of archaeological and faunal finds at Liang Bua (Flores, Indonesia) in light of the revised chronology for Homo floresiensis. *J Hum Evol* **124**, 52-74 (2018).
21 Koesbardiati, T., Murti, D. B., Herina, D. A. & Sari, A. A. The occurrence of enamel hypoplasia, porotic hyperostosis and cribra orbitalia in three prehistoric skeletal assemblages from Indonesia. *Bull Int Assoc Paleodont* **12**, 33-40 (2018).
22 Soejono, R. Prehistoric development in Indonesia. In *Research Conference on Early Southeast Asia*, 8-13 (1985)
23 Koesbardiati, T. & Suriyanto, R. Dental modification in Flores: a biocultural perspective. *Recent advances on Southeast Asian paleoanthropology and archaeology. Yogyakarta: Yogyakarta: Laboratory of Bioanthropology and Paleoanthropology Faculty of Medicine Gadjah Mada University*, 259-268 (2007).
24 Morwood, M. J. Time, space and prehistoric art: a principal components analysis. *Archaeology & Physical Anthropology in Oceania* **15**, 98-109 (1980).
25 Kinaston, R. L. *et al.* Ritual tooth ablation and the Austronesian expansion: Evidence from eastern Indonesia and the Pacific Islands. *The J Island Coast Archaeol*, 1-32 (2020).
26 Koesbardiati, T., Murti, D. B. & Suriyanto, R. A. Cultural dental modification in prehistoric population in Indonesia. *Bull Int Assoc Paleodont* **9**, 52-60 (2015).
27 Suriyanto, R., Koesbardiati, T., Murti, D. & Indriati, E. In *Proceeding Book the 2nd International Joint Symposium on Oral and Dental Sciences*, 213-224 (2012).
28 Hooijer, D. Three new giant prehistoric rats from Flores, Lesser Sunda Islands. *Zoologische Mededelingen* **35**, 299-314 (1957).
29 Sukadana, A. Tengkorak-tengkorak purba dari P. Komodo. *Kumpulan Naskah Pertemuan Nasional Ahli Anatomi Indonesia III* (1973).
30 Sukadana, A. Studi Politipisme dan Polimorfisme populasi pada beberapa peninggalan di Nusa Tenggara Timur. *Disertasi. Surabaya: Universitas Airlangga. Tidak dipublikasikan* (1984).
31 Ricklefs, M. C. *A History of Modern Indonesia since c. 1200* (Macmillan International Higher Education, 2008).
32 Hawkins, S. *et al.* Metal-Age maritime culture at Jareng Bori rockshelter, Pantar Island, Eastern Indonesia. *Rec Aust Mus* **72**, 237-262 (2020).
33 Kasnowihardjo, G., Suriyanto, R. A., Koesbardiati, T. & Murti, D. B. Human Teeth Modification in Binangun and Leran: New findings in the Northern Coast of Rembang District, Central Java. *Berkala Arkeologi* **33** (2013).

Supplementary Figure 1 - DyStruct results. A-B Conditional log likelihood (CLL) values across K = 2 to K = 15 obtained for 25 independent runs (grey circles) using dataset 1 (A) and dataset 2 (B). Black dots show the average CLL per K. The yellow bar indicates the value of K at which the CLL starts to plateau. C-D ADMIXTURE-like barplots for K = 2 to K = 15 for dataset 1 (C) and dataset 2 (D). E-F Expanded barplots for the highlighted K value of each respective dataset: K= 13 for dataset 1 (E) and K = 9 for dataset 2 (F).

A)

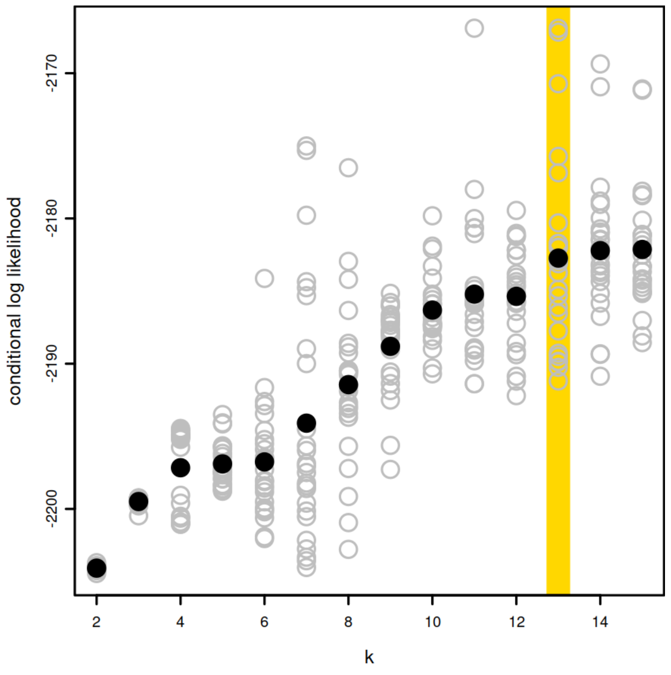

B)

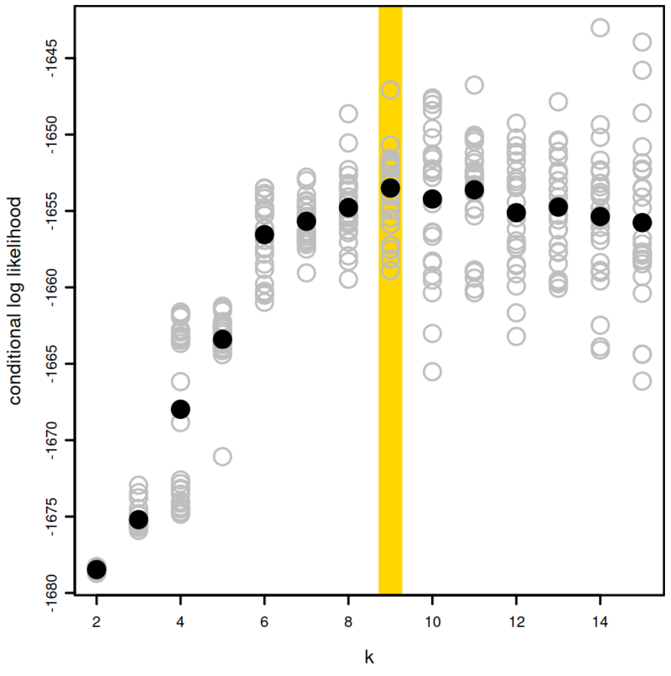

C)

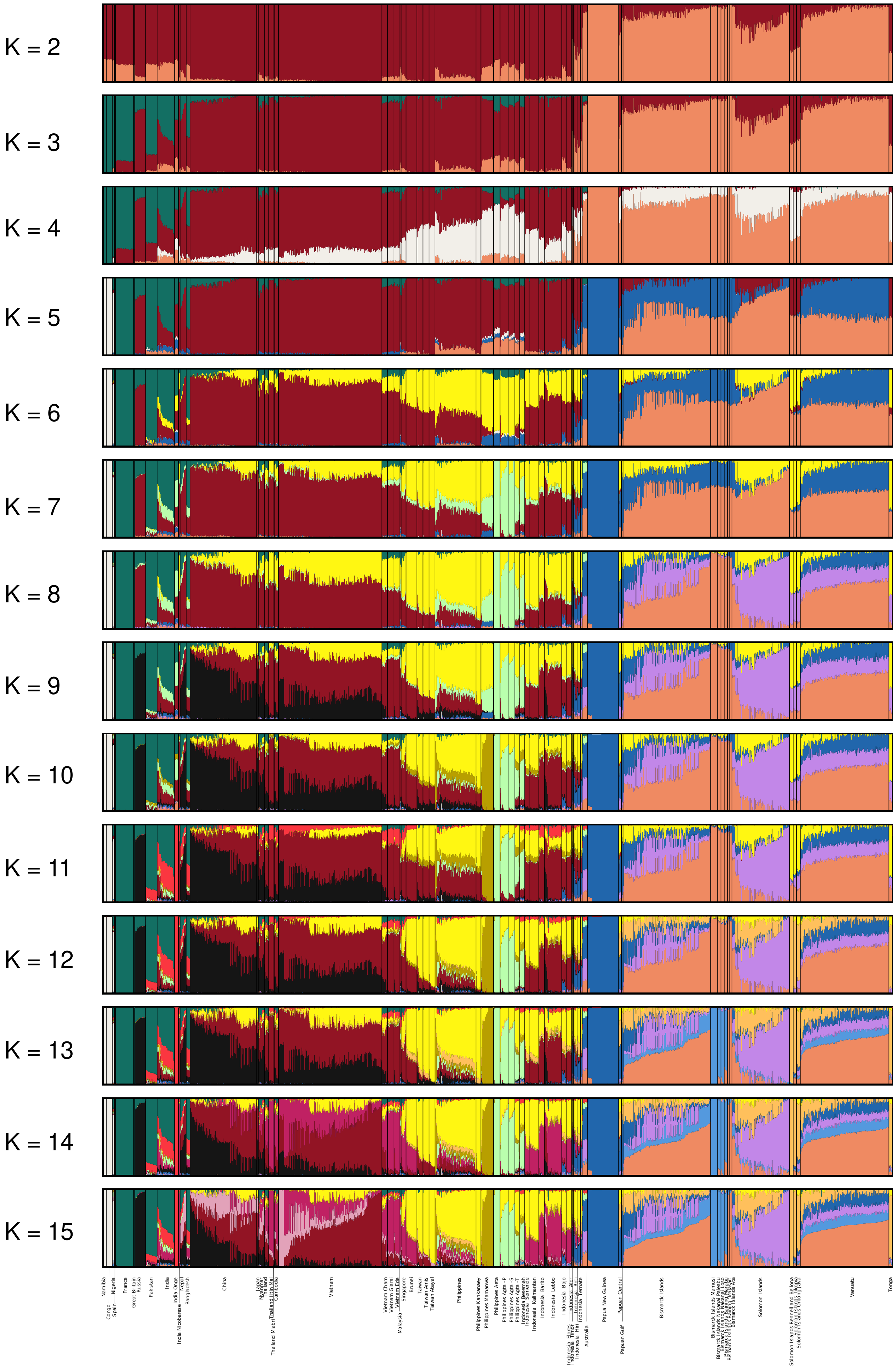
K = 2
K = 3
K = 4
K = 5
K = 6
K = 7
K = 8
K = 9
K = 10
K = 11
K = 12
K = 13
K = 14
K = 15
Namibia
Congo
Nigeria
Spain
France
Great Britain
Russia
Pakistan
India
India Onge
India Nicobarese
Nepal
Bangladesh
China
Japan
Thailand
Cambodia
Vietnam
Vietnam Cham
Vietnam Giarai
Vietnam Ede
Malaysia
Singapore
Brunei
Taiwan
Taiwan Amis
Taiwan Atayal
Philippines
Philippines Kankanaey
Philippines Mamanwa
Philippines Aeta
Indonesia Kalimantan
Indonesia Barito
Indonesia Lebbo
Indonesia Bajo
Australia
Papua New Guinea
Papuan Central
Papuan Gulf
Bismarck Islands
Bismarck Islands Mamusi
Solomon Islands
Vanuatu
Tonga

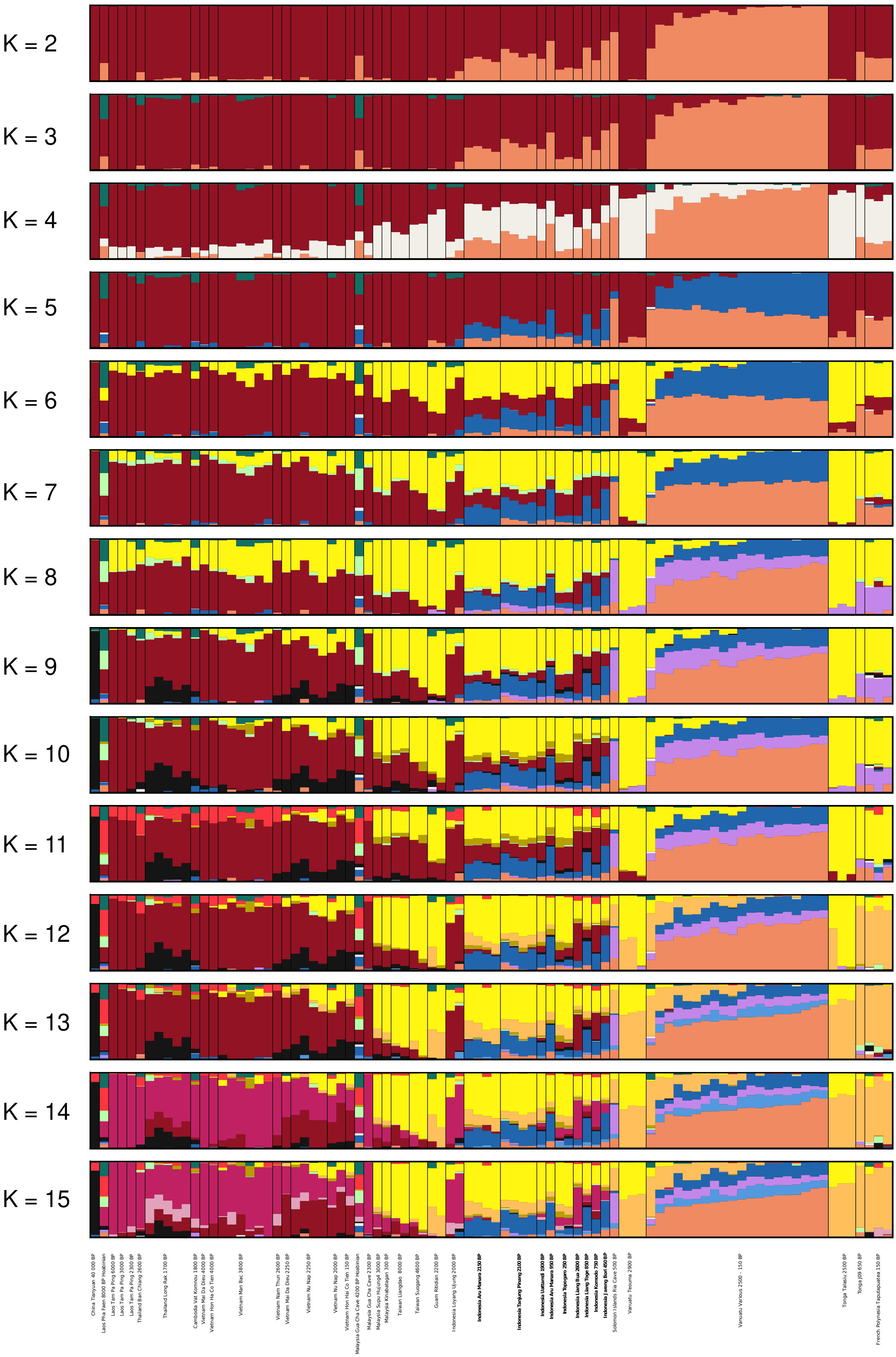
K = 2
K = 3
K = 4
K = 5
K = 6
K = 7
K = 8
K = 9
K = 10
K = 11
K = 12
K = 13
K = 14
K = 15
China Tianyuan 40 000 BP
Laos Pha Faen 8000 BP Hoabinian
Laos Tam Pa Ping 6000 BP
Laos Tam Pa Ping 3000 BP
Laos Tam Pa Ping 2300 BP
Thailand Ban Chiang 2600 BP
Thailand Long Rak 1700 BP
Cambodia Vat Komnou 1800 BP
Vietnam Mai Da Dieu 4000 BP
Vietnam Hon Ha Co Tien 4000 BP
Vietnam Man Bac 3800 BP
Vietnam Nam Thun 2600 BP
Vietnam Mai Da Dieu 2250 BP
Vietnam Nu Nap 2250 BP
Vietnam Nu Nap 2000 BP
Vietnam Hon Hai Co Tien 150 BP
Malaysia Gua Cha Cave 4200 BP Hoabinian
Malaysia Gua Cha Cave 2300 BP
Malaysia Supu Hujung4 3000 BP
Malaysia Kinabatagan 300 BP
Taiwan Liangdao 8000 BP
Taiwan Suogang 4600 BP
Guam Ritidian 2200 BP
Indonesia Loyang Ujung 2000 BP
Indonesia Aru Manara 2150 BP
Indonesia Tanjung Pinang 2100 BP
Indonesia Uattamdi 1900 BP
Indonesia Aru Manara 950 BP
Indonesia Topogaro 250 BP
Indonesia Liang Bua 2600 BP
Indonesia Liang Toge 850 BP
Indonesia Komodo 750 BP
Indonesia Jareng Bori 450 BP
Solomon Islands Ria Cave 500 BP
Vanuatu Teouma 2900 BP
Vanuatu Various 2500 - 150 BP
Tonga Talasiu 2500 BP
Tonga J09 650 BP
French Polynesia Taputapuatea 150 BP

D)

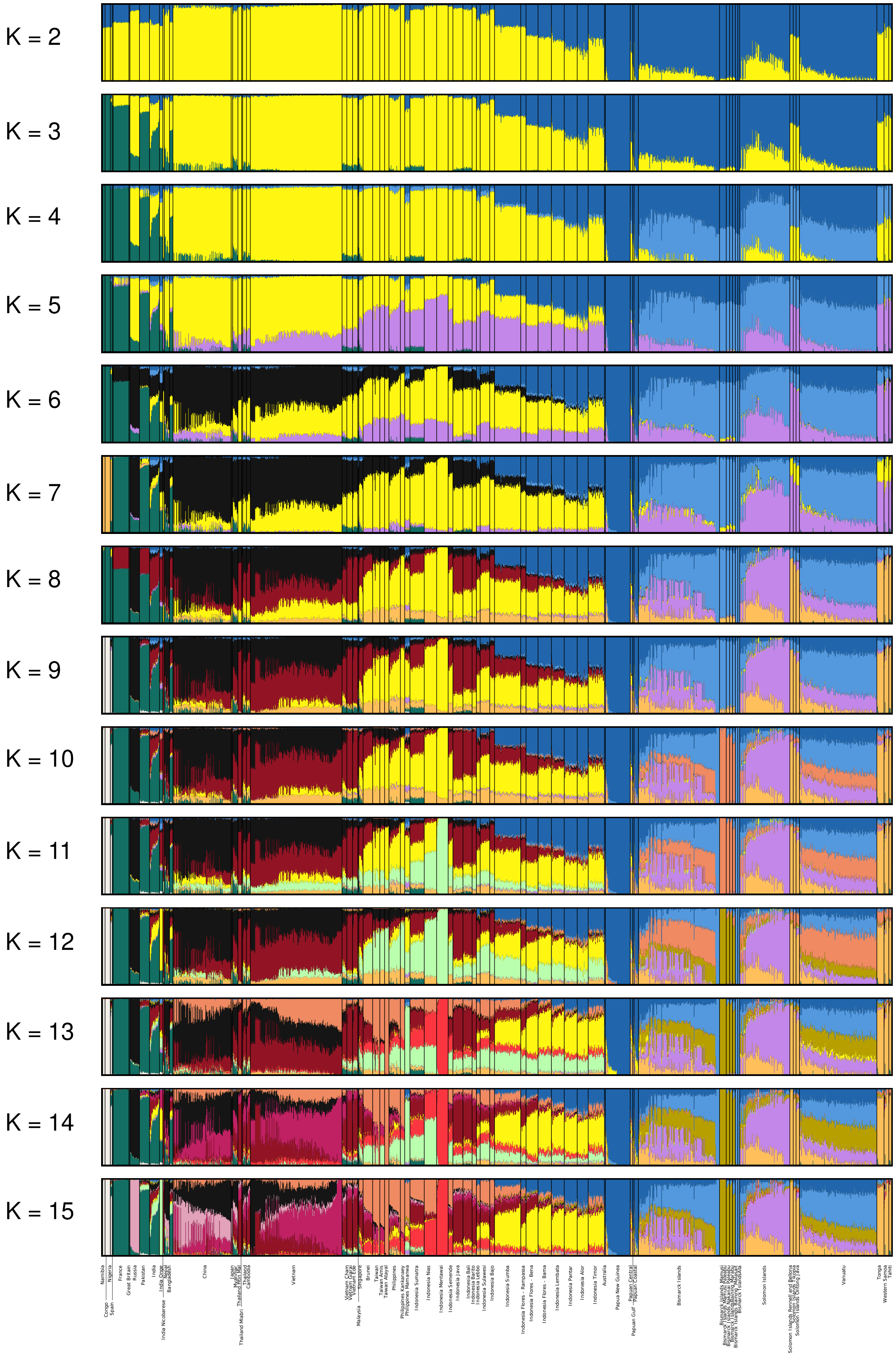
K = 2
K = 3
K = 4
K = 5
K = 6
K = 7
K = 8
K = 9
K = 10
K = 11
K = 12
K = 13
K = 14
K = 15
Namibia
Congo
Nigeria
Spain
France
Great Britain
Russia
Pakistan
India
India Nicobarese
India Onge
Nepal
Bangladesh
China
Japan
Myanmar
Thailand Mlabri
Thailand Htin Mal
Thailand
Cambodia
Vietnam
Vietnam Cham
Vietnam Ede
Malaysia
Singapore
Brunei
Taiwan
Taiwan Amis
Taiwan Atayal
Philippines
Philippines Kankanaey
Philippines Mamanwa
Indonesia Sumatra
Indonesia Nias
Indonesia Mentawai
Indonesia Semende
Indonesia Java
Indonesia Bali
Indonesia Barito
Indonesia Jabbo
Indonesia Sulawesi
Indonesia Bajo
Indonesia Sumba
Indonesia Flores - Rampasasa
Indonesia Flores - Bena
Indonesia Flores - Bama
Indonesia Lembata
Indonesia Pantar
Indonesia Alor
Indonesia Timor
Australia
Papua New Guinea
Papuan Gulf
Papuan Central
Papuan Coastal
Bismarck Islands
Solomon Islands
Solomon Islands Rennell and Bellona
Solomon Islands Tikopia
Solomon Islands Ontong Java
Vanuatu
Tonga
Western Samoa
Tahiti

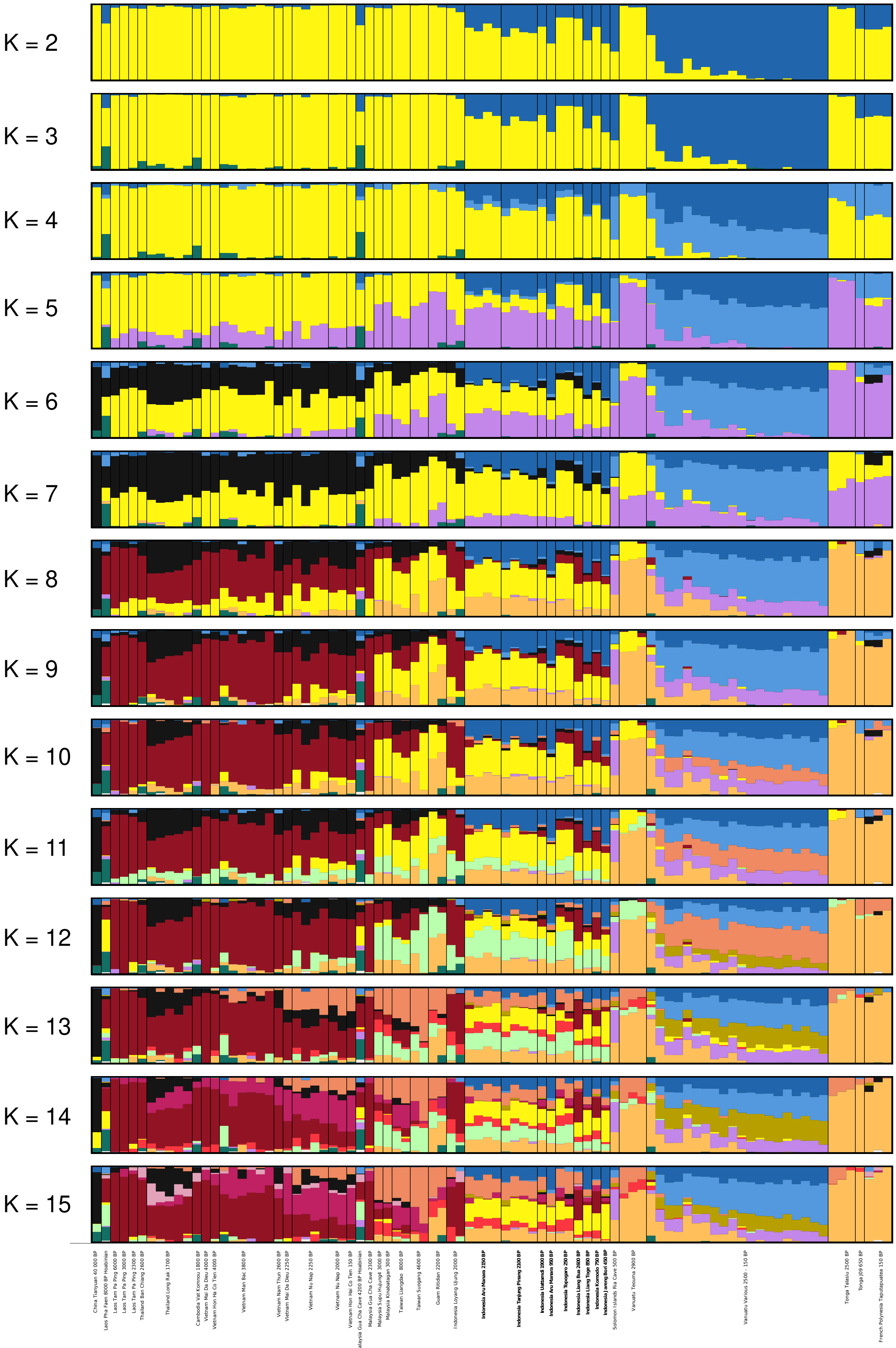
K = 2
K = 3
K = 4
K = 5
K = 6
K = 7
K = 8
K = 9
K = 10
K = 11
K = 12
K = 13
K = 14
K = 15
China Tianyuan 40 000 BP
Laos Pha Faen 8000 BP Hoabinian
Laos Tam Pa Ping 6000 BP
Laos Tam Pa Ping 3000 BP
Laos Tam Pa Ping 2300 BP
Thailand Ban Chiang 2600 BP
Thailand Long Rak 1700 BP
Cambodia Vat Komnou 1800 BP
Vietnam Mai Da Dieu 4000 BP
Vietnam Hon Ha Co Tien 4000 BP
Vietnam Man Bac 3800 BP
Vietnam Nam Thun 2600 BP
Vietnam Mai Da Dieu 2250 BP
Vietnam Nu Nap 2250 BP
Vietnam Nu Nap 2000 BP
Vietnam Hon Hai Co Tien 150 BP
Malaysia Gua Cha Cave 4200 BP Hoabinian
Malaysia Gua Cha Cave 2300 BP
Malaysia Supu Hujung4 3000 BP
Malaysia Kinabatagan 300 BP
Taiwan Liangdao 8000 BP
Taiwan Suogang 4600 BP
Guam Ritidian 2200 BP
Indonesia Loyang Ujung 2000 BP
Indonesia Aru Manara 2150 BP
Indonesia Tanjung Pinang 2100 BP
Indonesia Uattamdi 1900 BP
Indonesia Aru Manara 950 BP
Indonesia Topogaro 250 BP
Indonesia Liang Bua 2600 BP
Indonesia Liang Toge 850 BP
Indonesia Komodo 750 BP
Indonesia Jareng Bori 450 BP
Solomon Islands Ria Cave 500 BP
Vanuatu Teouma 2900 BP
Vanuatu Various 2500 - 150 BP
Tonga Talasiu 2500 BP
Tonga J09 650 BP
French Polynesia Taputapuatea 150 BP

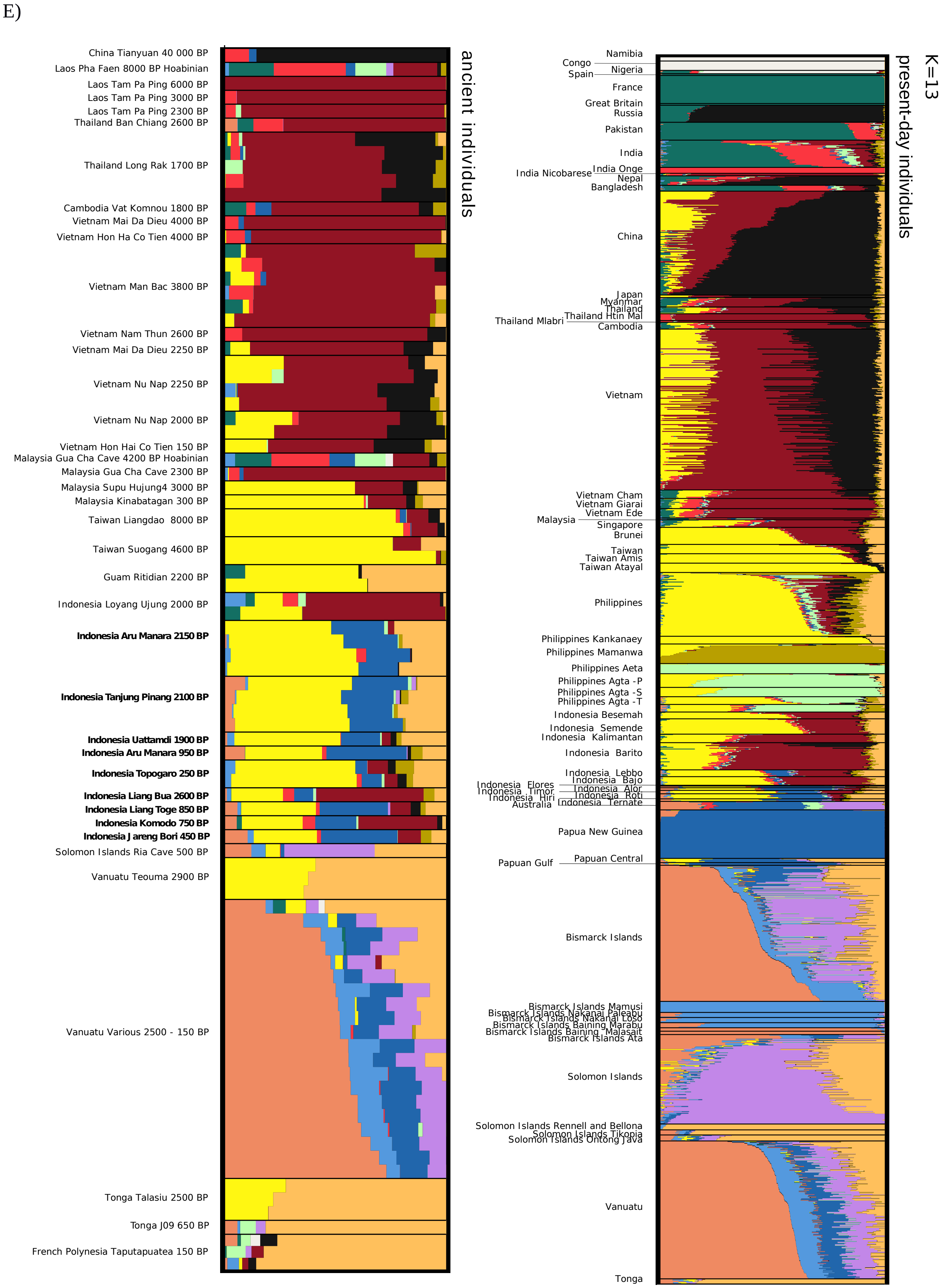
E)
China Tianyuan 40 000 BP
Laos Pha Faen 8000 BP Hoabinian
Laos Tam Pa Ping 6000 BP
Laos Tam Pa Ping 3000 BP
Laos Tam Pa Ping 2300 BP
Thailand Ban Chiang 2600 BP
Thailand Long Rak 1700 BP
Cambodia Vat Komnou 1800 BP
Vietnam Mai Da Dieu 4000 BP
Vietnam Hon Ha Co Tien 4000 BP
Vietnam Man Bac 3800 BP
Vietnam Nam Thun 2600 BP
Vietnam Mai Da Dieu 2250 BP
Vietnam Nu Nap 2250 BP
Vietnam Nu Nap 2000 BP
Vietnam Hon Hai Co Tien 150 BP
Malaysia Gua Cha Cave 4200 BP Hoabinian
Malaysia Gua Cha Cave 2300 BP
Malaysia Supu Hujung4 3000 BP
Malaysia Kinabatagan 300 BP
Taiwan Liangdao 8000 BP
Taiwan Suogang 4600 BP
Guam Ritidian 2200 BP
Indonesia Loyang Ujung 2000 BP
Indonesia Aru Manara 2150 BP
Indonesia Tanjung Pinang 2100 BP
Indonesia Uattamdi 1900 BP
Indonesia Aru Manara 950 BP
Indonesia Topogaro 250 BP
Indonesia Liang Bua 2600 BP
Indonesia Liang Toge 850 BP
Indonesia Komodo 750 BP
Indonesia J areng Bori 450 BP
Solomon Islands Ria Cave 500 BP
Vanuatu Teouma 2900 BP
Vanuatu Various 2500 - 150 BP
Tonga Talasiu 2500 BP
Tonga J09 650 BP
French Polynesia Taputapuatea 150 BP
ancient individuals
Namibia
Congo
Nigeria
Spain
France
Great Britain
Russia
Pakistan
India
India Nicobarese
India Onge
Nepal
Bangladesh
China
Japan
Myanmar
Thailand
Thailand Mlabri
Thailand Htin Mal
Cambodia
Vietnam
Vietnam Cham
Vietnam Giarai
Vietnam Ede
Malaysia
Singapore
Brunei
Taiwan
Taiwan Amis
Taiwan Atayal
Philippines
Philippines Kankanaey
Philippines Mamanwa
Philippines Aeta
Philippines Agta -P
Philippines Agta -S
Philippines Agta -T
Indonesia Besemah
Indonesia Semende
Indonesia Kalimantan
Indonesia Barito
Indonesia Lebbo
Indonesia Bajo
Indonesia Flores
Indonesia Timor
Indonesia Alor
Indonesia Hiri
Indonesia Roti
Australia
Indonesia Ternate
Papua New Guinea
Papuan Gulf
Papuan Central
Bismarck Islands
Bismarck Islands Mamusi
Bismarck Islands Nakanai Paleabu
Bismarck Islands Nakanai Loso
Bismarck Islands Baining Marabu
Bismarck Islands Baining Malasait
Bismarck Islands Ata
Solomon Islands
Solomon Islands Rennell and Bellona
Solomon Islands Tikopia
Solomon Islands Ontong Java
Vanuatu
Tonga
present-day individuals
K=13

F)

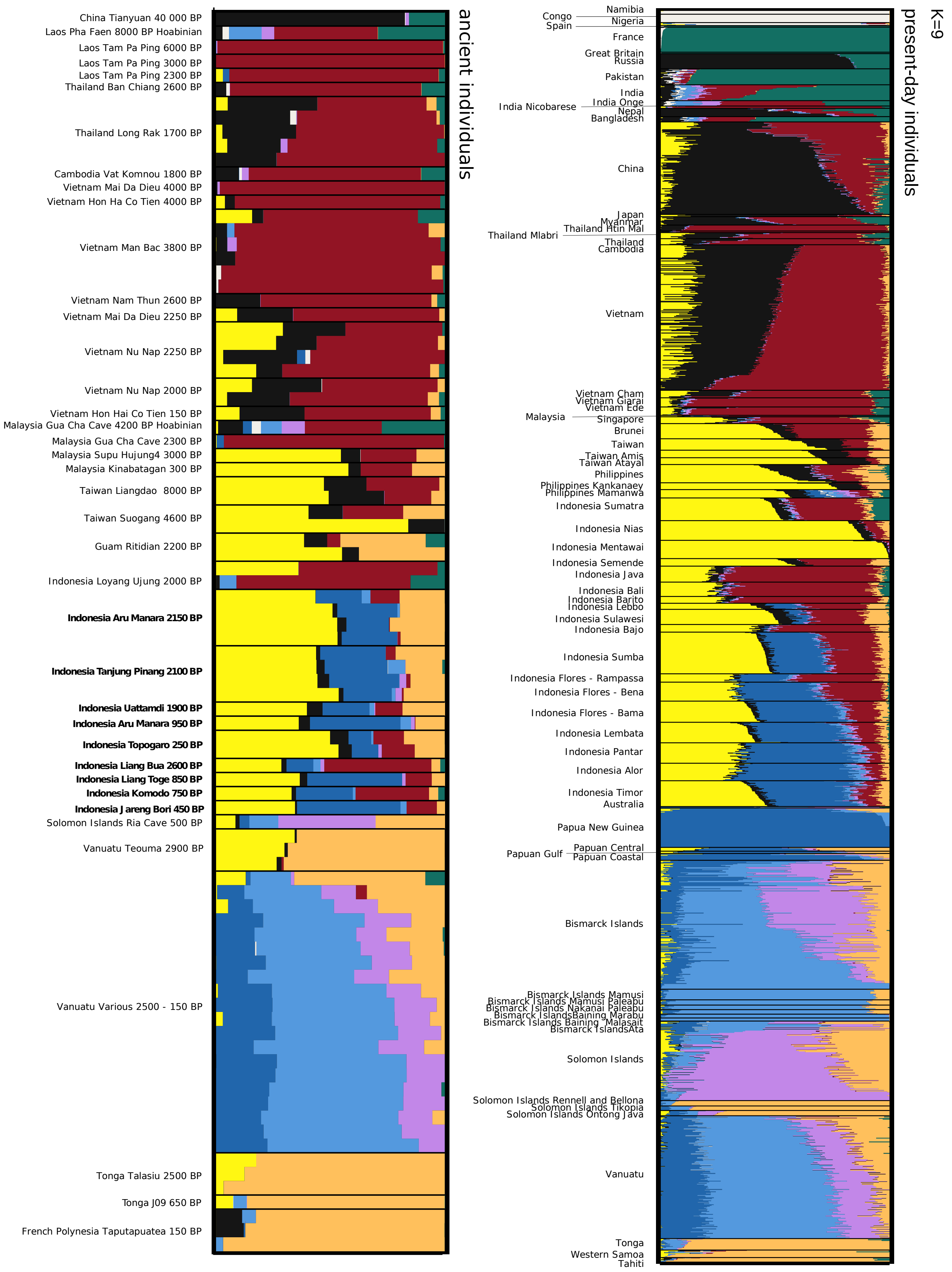
China Tianyuan 40 000 BP
Laos Pha Faen 8000 BP Hoabinian
Laos Tam Pa Ping 6000 BP
Laos Tam Pa Ping 3000 BP
Laos Tam Pa Ping 2300 BP
Thailand Ban Chiang 2600 BP
Thailand Long Rak 1700 BP
Cambodia Vat Komnou 1800 BP
Vietnam Mai Da Dieu 4000 BP
Vietnam Hon Ha Co Tien 4000 BP
Vietnam Man Bac 3800 BP
Vietnam Nam Thun 2600 BP
Vietnam Mai Da Dieu 2250 BP
Vietnam Nu Nap 2250 BP
Vietnam Nu Nap 2000 BP
Vietnam Hon Hai Co Tien 150 BP
Malaysia Gua Cha Cave 4200 BP Hoabinian
Malaysia Gua Cha Cave 2300 BP
Malaysia Supu Hujung4 3000 BP
Malaysia Kinabatagan 300 BP
Taiwan Liangdao 8000 BP
Taiwan Suogang 4600 BP
Guam Ritidian 2200 BP
Indonesia Loyang Ujung 2000 BP
Indonesia Aru Manara 2150 BP
Indonesia Tanjung Pinang 2100 BP
Indonesia Uattamdi 1900 BP
Indonesia Aru Manara 950 BP
Indonesia Topogaro 250 BP
Indonesia Liang Bua 2600 BP
Indonesia Liang Toge 850 BP
Indonesia Komodo 750 BP
Indonesia Jareng Bori 450 BP
Solomon Islands Ria Cave 500 BP
Vanuatu Teouma 2900 BP
Vanuatu Various 2500 - 150 BP
Tonga Talasiu 2500 BP
Tonga J09 650 BP
French Polynesia Taputapuatea 150 BP
ancient individuals
Namibia
Congo
Nigeria
Spain
France
Great Britain
Russia
Pakistan
India
India Onge
India Nicobarese
Nepal
Bangladesh
China
Japan
Myanmar
Thailand Htin Mal
Thailand Mlabri
Thailand
Cambodia
Vietnam
Vietnam Cham
Vietnam Giarai
Vietnam Ede
Malaysia
Singapore
Brunei
Taiwan
Taiwan Amis
Taiwan Atayal
Philippines
Philippines Kankanaey
Philippines Mamanwa
Indonesia Sumatra
Indonesia Nias
Indonesia Mentawai
Indonesia Semende
Indonesia Java
Indonesia Bali
Indonesia Barito
Indonesia Lebbo
Indonesia Sulawesi
Indonesia Bajo
Indonesia Sumba
Indonesia Flores - Rampassa
Indonesia Flores - Bena
Indonesia Flores - Bama
Indonesia Lembata
Indonesia Pantar
Indonesia Alor
Indonesia Timor
Australia
Papua New Guinea
Papuan Central
Papuan Gulf
Papuan Coastal
Bismarck Islands
Bismarck Islands Mamusi
Bismarck Islands Mamusi Paleabu
Bismarck Islands Nakanai Paleabu
Bismarck IslandsBaining Marabu
Bismarck Islands Baining Malasait
Bismarck IslandsAta
Solomon Islands
Solomon Islands Rennell and Bellona
Solomon Islands Tikopia
Solomon Islands Ontong Java
Vanuatu
Tonga
Western Samoa
Tahiti
present-day individuals
K=9

Supplementary Figure 2 - *f_4*-statistic of the form *f_4(Mbuti, ancient Wallacean; Amis, test)* computed for each ancient group from Wallacea separately. The test groups are shown in the y-axis and include ancient and present-day groups from mainland Asia, Island southeast Asia, and the Pacific that have no discernible Papuan-related ancestry. Data are presented as exact f_4-values ± 2 SE. Values in green are not significantly different from zero (|Z| < 2), whereas values in red are significantly different from zero (|Z| > 2).

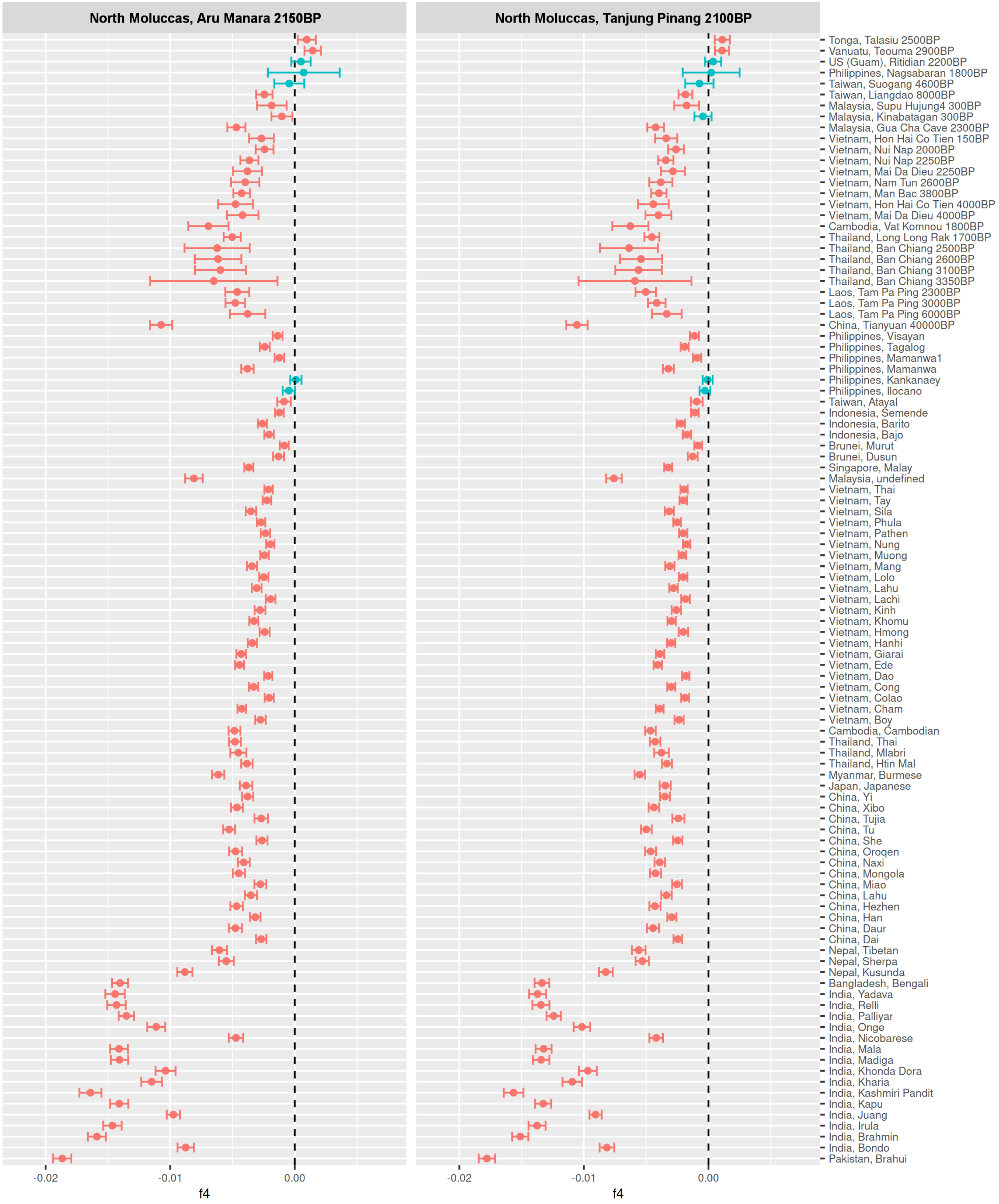

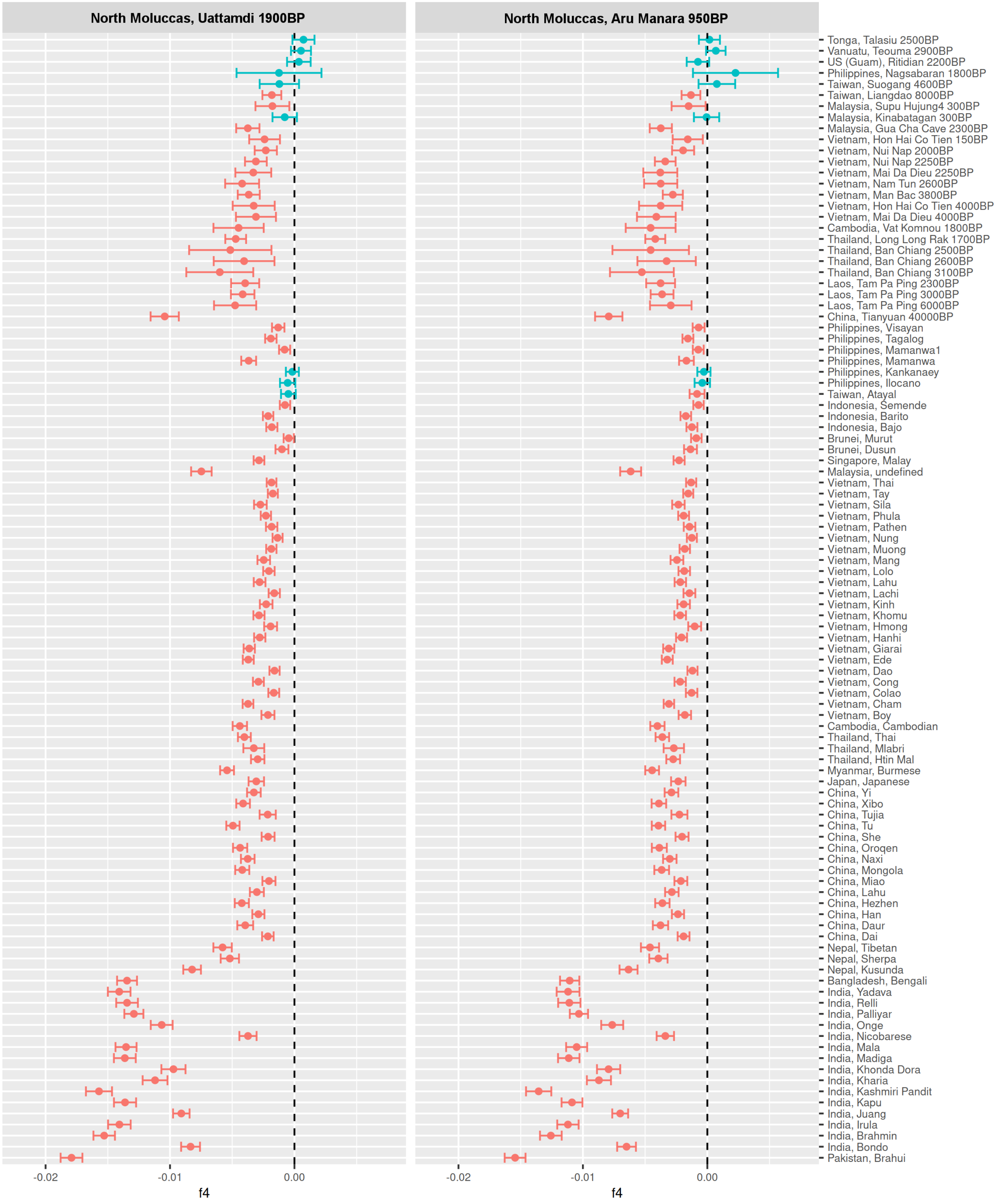

North Moluccas, Uattamdi 1900BP
North Moluccas, Aru Manara 950BP
Tonga, Talasiu 2500BP
Vanuatu, Teouma 2900BP
US (Guam), Ritidian 2200BP
Philippines, Nagsabaran 1800BP
Taiwan, Suogang 4600BP
Taiwan, Liangdao 8000BP
Malaysia, Supu Hujung4 300BP
Malaysia, Kinabatagan 300BP
Malaysia, Gua Cha Cave 2300BP
Vietnam, Hon Hai Co Tien 150BP
Vietnam, Nui Nap 2000BP
Vietnam, Nui Nap 2250BP
Vietnam, Mai Da Dieu 2250BP
Vietnam, Nam Tun 2600BP
Vietnam, Man Bac 3800BP
Vietnam, Hon Hai Co Tien 4000BP
Vietnam, Mai Da Dieu 4000BP
Cambodia, Vat Komnou 1800BP
Thailand, Long Long Rak 1700BP
Thailand, Ban Chiang 2500BP
Thailand, Ban Chiang 2600BP
Thailand, Ban Chiang 3100BP
Laos, Tam Pa Ping 2300BP
Laos, Tam Pa Ping 3000BP
Laos, Tam Pa Ping 6000BP
China, Tianyuan 40000BP
Philippines, Visayan
Philippines, Tagalog
Philippines, Mamanwa1
Philippines, Mamanwa
Philippines, Kankanaey
Philippines, Ilocano
Taiwan, Atayal
Indonesia, Semende
Indonesia, Barito
Indonesia, Bajo
Brunei, Murut
Brunei, Dusun
Singapore, Malay
Malaysia, undefined
Vietnam, Thai
Vietnam, Tay
Vietnam, Sila
Vietnam, Phula
Vietnam, Pathen
Vietnam, Nung
Vietnam, Muong
Vietnam, Mang
Vietnam, Lolo
Vietnam, Lahu
Vietnam, Lachi
Vietnam, Kinh
Vietnam, Khomu
Vietnam, Hmong
Vietnam, Hanhi
Vietnam, Giarai
Vietnam, Ede
Vietnam, Dao
Vietnam, Cong
Vietnam, Colao
Vietnam, Cham
Vietnam, Boy
Cambodia, Cambodian
Thailand, Thai
Thailand, Mlabri
Thailand, Htin Mal
Myanmar, Burmese
Japan, Japanese
China, Yi
China, Xibo
China, Tujia
China, Tu
China, She
China, Oroqen
China, Naxi
China, Mongola
China, Miao
China, Lahu
China, Hezhen
China, Han
China, Daur
China, Dai
Nepal, Tibetan
Nepal, Sherpa
Nepal, Kusunda
Bangladesh, Bengali
India, Yadava
India, Relli
India, Palliyar
India, Onge
India, Nicobarese
India, Mala
India, Madiga
India, Khonda Dora
India, Kharia
India, Kashmiri Pandit
India, Kapu
India, Juang
India, Irula
India, Brahmin
India, Bondo
Pakistan, Brahui
-0.02
-0.01
0.00
f4

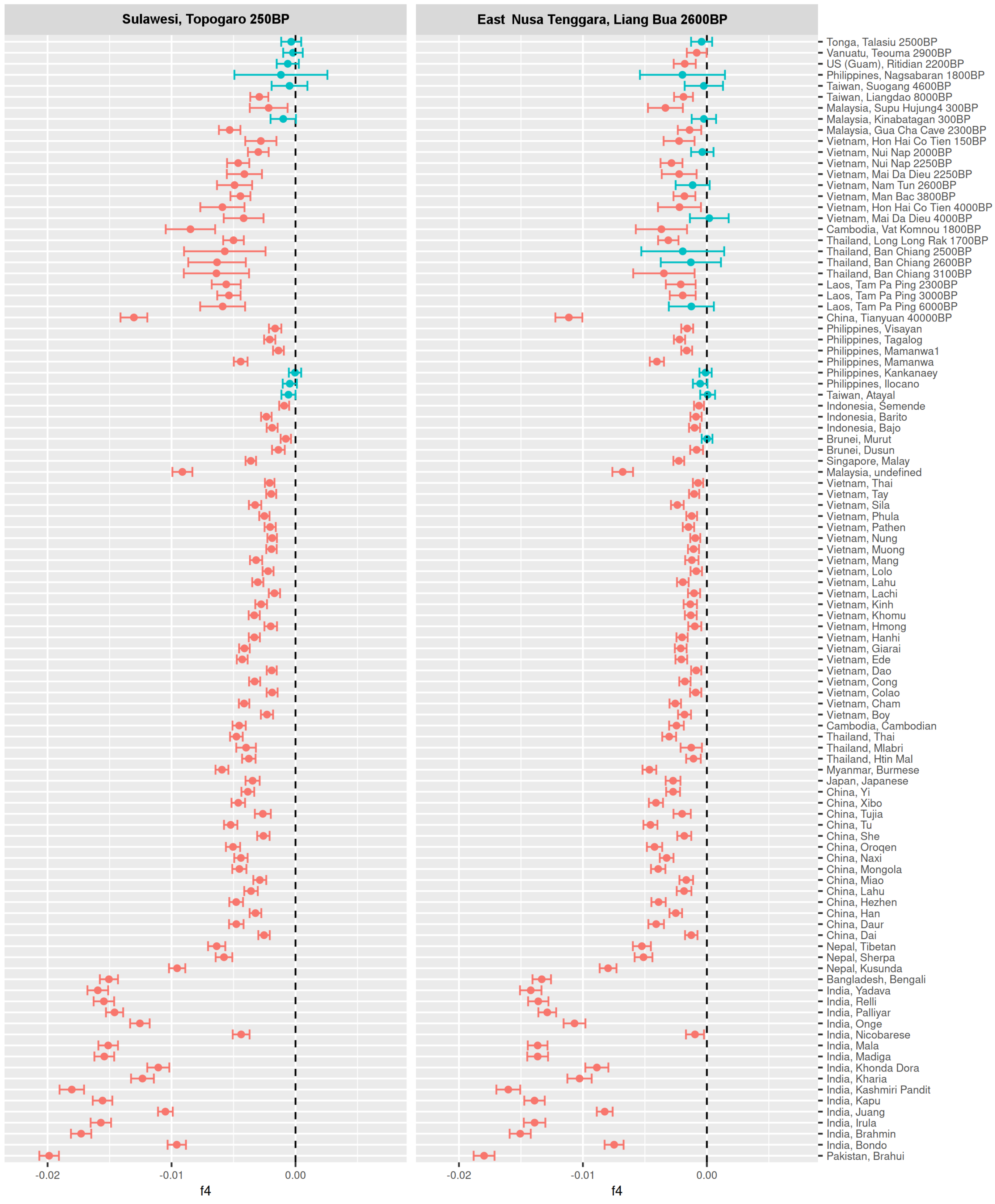

Sulawesi, Topogaro 250BP
East Nusa Tenggara, Liang Bua 2600BP
Tonga, Talasiu 2500BP
Vanuatu, Teouma 2900BP
US (Guam), Ritidian 2200BP
Philippines, Nagsabaran 1800BP
Taiwan, Suogang 4600BP
Taiwan, Liangdao 8000BP
Malaysia, Supu Hujung4 300BP
Malaysia, Kinabatagan 300BP
Malaysia, Gua Cha Cave 2300BP
Vietnam, Hon Hai Co Tien 150BP
Vietnam, Nui Nap 2000BP
Vietnam, Nui Nap 2250BP
Vietnam, Mai Da Dieu 2250BP
Vietnam, Nam Tun 2600BP
Vietnam, Man Bac 3800BP
Vietnam, Hon Hai Co Tien 4000BP
Vietnam, Mai Da Dieu 4000BP
Cambodia, Vat Komnou 1800BP
Thailand, Long Long Rak 1700BP
Thailand, Ban Chiang 2500BP
Thailand, Ban Chiang 2600BP
Thailand, Ban Chiang 3100BP
Laos, Tam Pa Ping 2300BP
Laos, Tam Pa Ping 3000BP
Laos, Tam Pa Ping 6000BP
China, Tianyuan 40000BP
Philippines, Visayan
Philippines, Tagalog
Philippines, Mamanwa1
Philippines, Mamanwa
Philippines, Kankanaey
Philippines, Ilocano
Taiwan, Atayal
Indonesia, Semende
Indonesia, Barito
Indonesia, Bajo
Brunei, Murut
Brunei, Dusun
Singapore, Malay
Malaysia, undefined
Vietnam, Thai
Vietnam, Tay
Vietnam, Sila
Vietnam, Phula
Vietnam, Pathen
Vietnam, Nung
Vietnam, Muong
Vietnam, Mang
Vietnam, Lolo
Vietnam, Lahu
Vietnam, Lachi
Vietnam, Kinh
Vietnam, Khomu
Vietnam, Hmong
Vietnam, Hanhi
Vietnam, Giarai
Vietnam, Ede
Vietnam, Dao
Vietnam, Cong
Vietnam, Colao
Vietnam, Cham
Vietnam, Boy
Cambodia, Cambodian
Thailand, Thai
Thailand, Mlabri
Thailand, Htin Mal
Myanmar, Burmese
Japan, Japanese
China, Yi
China, Xibo
China, Tujia
China, Tu
China, She
China, Oroqen
China, Naxi
China, Mongola
China, Miao
China, Lahu
China, Hezhen
China, Han
China, Daur
China, Dai
Nepal, Tibetan
Nepal, Sherpa
Nepal, Kusunda
Bangladesh, Bengali
India, Yadava
India, Relli
India, Palliyar
India, Onge
India, Nicobarese
India, Mala
India, Madiga
India, Khonda Dora
India, Kharia
India, Kashmiri Pandit
India, Kapu
India, Juang
India, Irula
India, Brahmin
India, Bondo
Pakistan, Brahui
-0.02
-0.01
0.00
f4

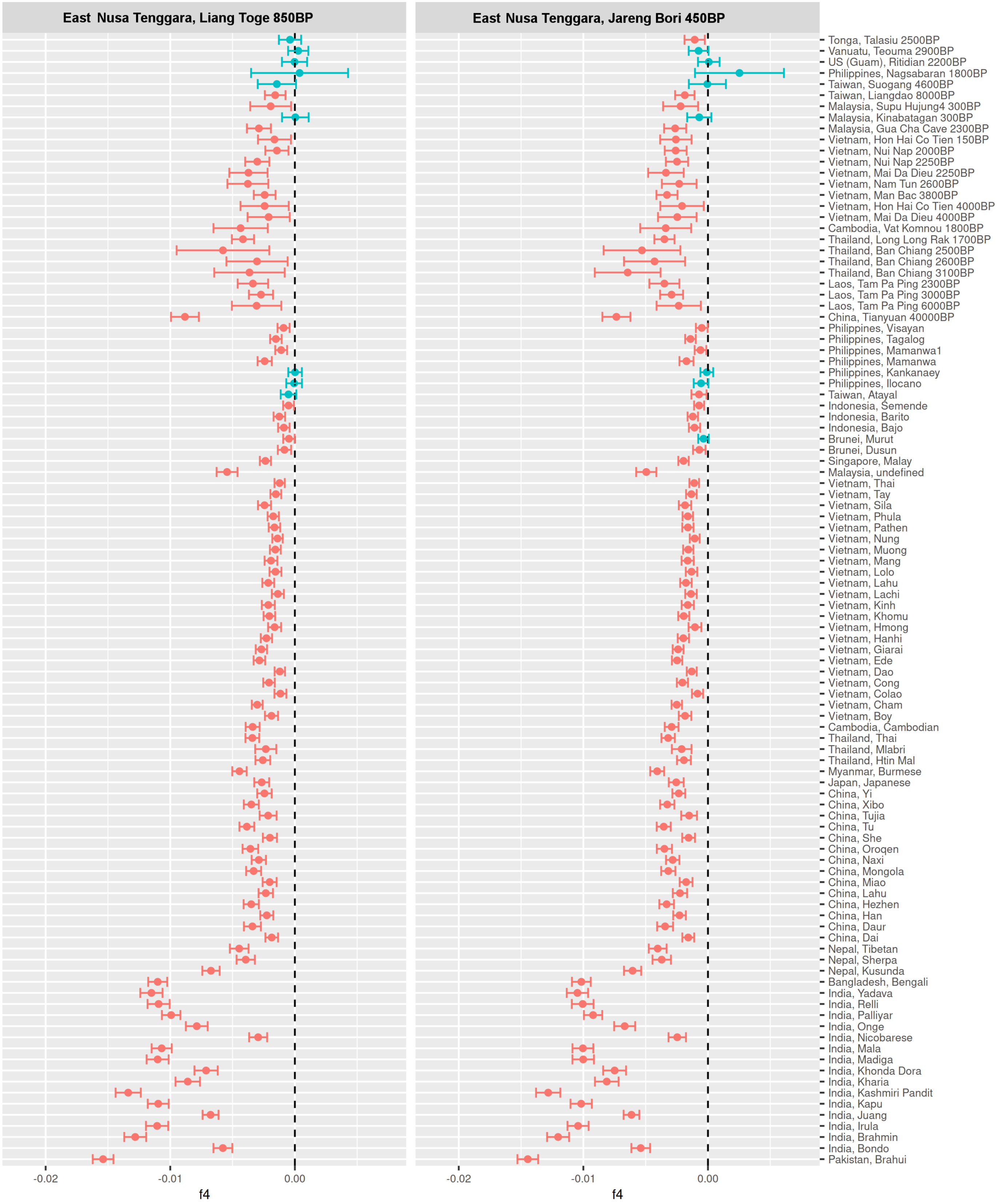
East Nusa Tenggara, Liang Toge 850BP
East Nusa Tenggara, Jareng Bori 450BP
Tonga, Talasiu 2500BP
Vanuatu, Teouma 2900BP
US (Guam), Ritidian 2200BP
Philippines, Nagsabaran 1800BP
Taiwan, Suogang 4600BP
Taiwan, Liangdao 8000BP
Malaysia, Supu Hujung4 300BP
Malaysia, Kinabatagan 300BP
Malaysia, Gua Cha Cave 2300BP
Vietnam, Hon Hai Co Tien 150BP
Vietnam, Nui Nap 2000BP
Vietnam, Nui Nap 2250BP
Vietnam, Mai Da Dieu 2250BP
Vietnam, Nam Tun 2600BP
Vietnam, Man Bac 3800BP
Vietnam, Hon Hai Co Tien 4000BP
Vietnam, Mai Da Dieu 4000BP
Cambodia, Vat Komnou 1800BP
Thailand, Long Long Rak 1700BP
Thailand, Ban Chiang 2500BP
Thailand, Ban Chiang 2600BP
Thailand, Ban Chiang 3100BP
Laos, Tam Pa Ping 2300BP
Laos, Tam Pa Ping 3000BP
Laos, Tam Pa Ping 6000BP
China, Tianyuan 40000BP
Philippines, Visayan
Philippines, Tagalog
Philippines, Mamanwa1
Philippines, Mamanwa
Philippines, Kankanaey
Philippines, Ilocano
Taiwan, Atayal
Indonesia, Semende
Indonesia, Barito
Indonesia, Bajo
Brunei, Murut
Brunei, Dusun
Singapore, Malay
Malaysia, undefined
Vietnam, Thai
Vietnam, Tay
Vietnam, Sila
Vietnam, Phula
Vietnam, Pathen
Vietnam, Nung
Vietnam, Muong
Vietnam, Mang
Vietnam, Lolo
Vietnam, Lahu
Vietnam, Lachi
Vietnam, Kinh
Vietnam, Khomu
Vietnam, Hmong
Vietnam, Hanhi
Vietnam, Giarai
Vietnam, Ede
Vietnam, Dao
Vietnam, Cong
Vietnam, Colao
Vietnam, Cham
Vietnam, Boy
Cambodia, Cambodian
Thailand, Thai
Thailand, Mlabri
Thailand, Htin Mal
Myanmar, Burmese
Japan, Japanese
China, Yi
China, Xibo
China, Tujia
China, Tu
China, She
China, Oroqen
China, Naxi
China, Mongola
China, Miao
China, Lahu
China, Hezhen
China, Han
China, Daur
China, Dai
Nepal, Tibetan
Nepal, Sherpa
Nepal, Kusunda
Bangladesh, Bengali
India, Yadava
India, Relli
India, Palliyar
India, Onge
India, Nicobarese
India, Mala
India, Madiga
India, Khonda Dora
India, Kharia
India, Kashmiri Pandit
India, Kapu
India, Juang
India, Irula
India, Brahmin
India, Bondo
Pakistan, Brahui
-0.02
-0.01
0.00
f4

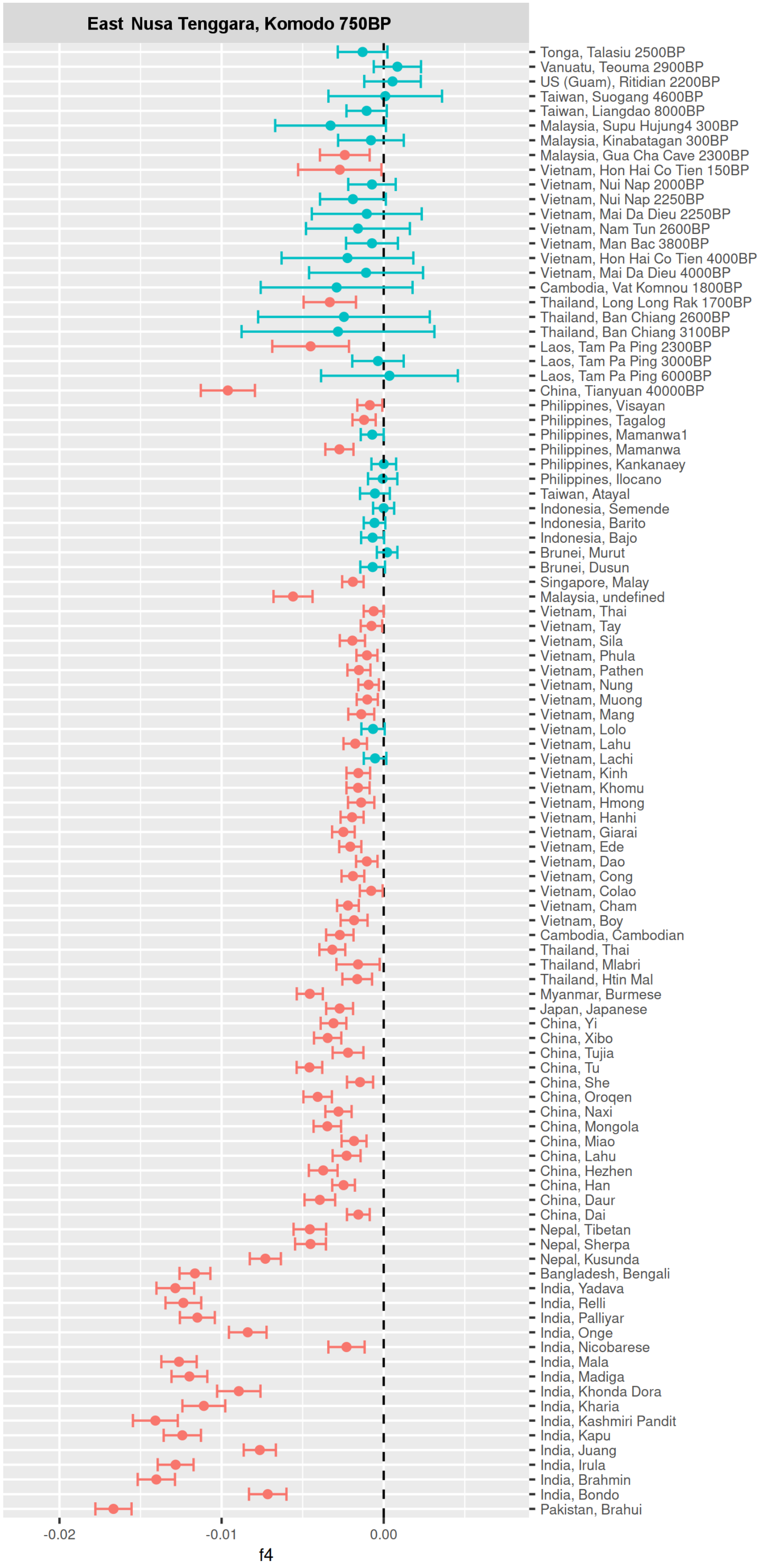
East Nusa Tenggara, Komodo 750BP
Tonga, Talasiu 2500BP
Vanuatu, Teouma 2900BP
US (Guam), Ritidian 2200BP
Taiwan, Suogang 4600BP
Taiwan, Liangdao 8000BP
Malaysia, Supu Hujung4 300BP
Malaysia, Kinabatagan 300BP
Malaysia, Gua Cha Cave 2300BP
Vietnam, Hon Hai Co Tien 150BP
Vietnam, Nui Nap 2000BP
Vietnam, Nui Nap 2250BP
Vietnam, Mai Da Dieu 2250BP
Vietnam, Nam Tun 2600BP
Vietnam, Man Bac 3800BP
Vietnam, Hon Hai Co Tien 4000BP
Vietnam, Mai Da Dieu 4000BP
Cambodia, Vat Komnou 1800BP
Thailand, Long Long Rak 1700BP
Thailand, Ban Chiang 2600BP
Thailand, Ban Chiang 3100BP
Laos, Tam Pa Ping 2300BP
Laos, Tam Pa Ping 3000BP
Laos, Tam Pa Ping 6000BP
China, Tianyuan 40000BP
Philippines, Visayan
Philippines, Tagalog
Philippines, Mamanwa1
Philippines, Mamanwa
Philippines, Kankanaey
Philippines, Ilocano
Taiwan, Atayal
Indonesia, Semende
Indonesia, Barito
Indonesia, Bajo
Brunei, Murut
Brunei, Dusun
Singapore, Malay
Malaysia, undefined
Vietnam, Thai
Vietnam, Tay
Vietnam, Sila
Vietnam, Phula
Vietnam, Pathen
Vietnam, Nung
Vietnam, Muong
Vietnam, Mang
Vietnam, Lolo
Vietnam, Lahu
Vietnam, Lachi
Vietnam, Kinh
Vietnam, Khomu
Vietnam, Hmong
Vietnam, Hanhi
Vietnam, Giarai
Vietnam, Ede
Vietnam, Dao
Vietnam, Cong
Vietnam, Colao
Vietnam, Cham
Vietnam, Boy
Cambodia, Cambodian
Thailand, Thai
Thailand, Mlabri
Thailand, Htin Mal
Myanmar, Burmese
Japan, Japanese
China, Yi
China, Xibo
China, Tujia
China, Tu
China, She
China, Oroqen
China, Naxi
China, Mongola
China, Miao
China, Lahu
China, Hezhen
China, Han
China, Daur
China, Dai
Nepal, Tibetan
Nepal, Sherpa
Nepal, Kusunda
Bangladesh, Bengali
India, Yadava
India, Relli
India, Palliyar
India, Onge
India, Nicobarese
India, Mala
India, Madiga
India, Khonda Dora
India, Kharia
India, Kashmiri Pandit
India, Kapu
India, Juang
India, Irula
India, Brahmin
India, Bondo
Pakistan, Brahui
-0.02
-0.01
0.00
f4

Supplementary Figure 3 - Biplots showing the results of pairs of f_4-statistics of the form: f_4(Mbuti, test; New Guinea Highlanders, ancient Wallacea). The test groups, shown on the x-y axis label, include present-day groups from mainland Asia, Island southeast Asia, and Oceania that have no discernible Papuan-related ancestry based on the DyStruct analysis. Data are presented as exact f_4-values ± 2 SE indicated by grey lines. Linear regression lines for the North Moluccas and East Nusa Tenggara individuals are shown in green and red, respectively.

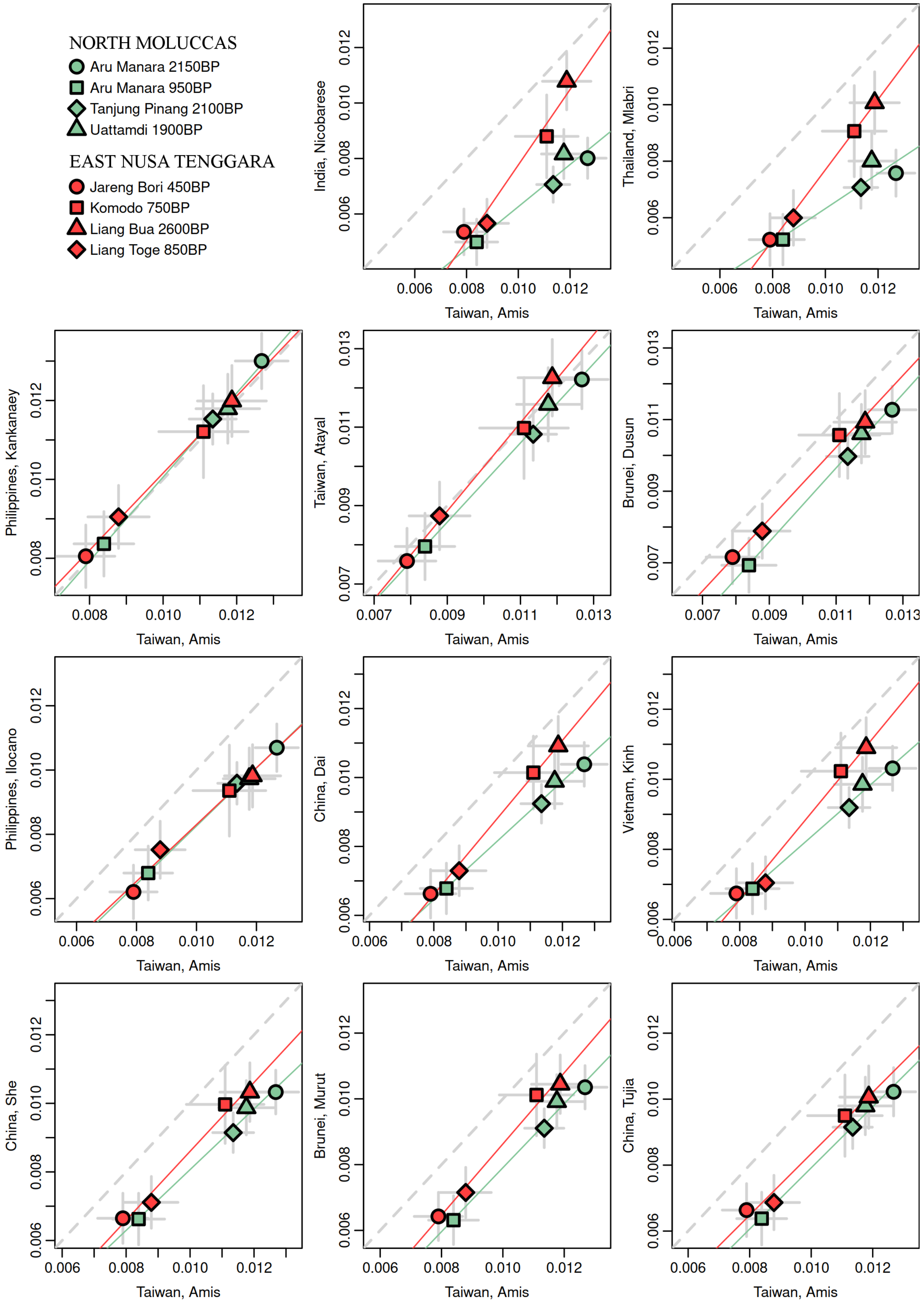

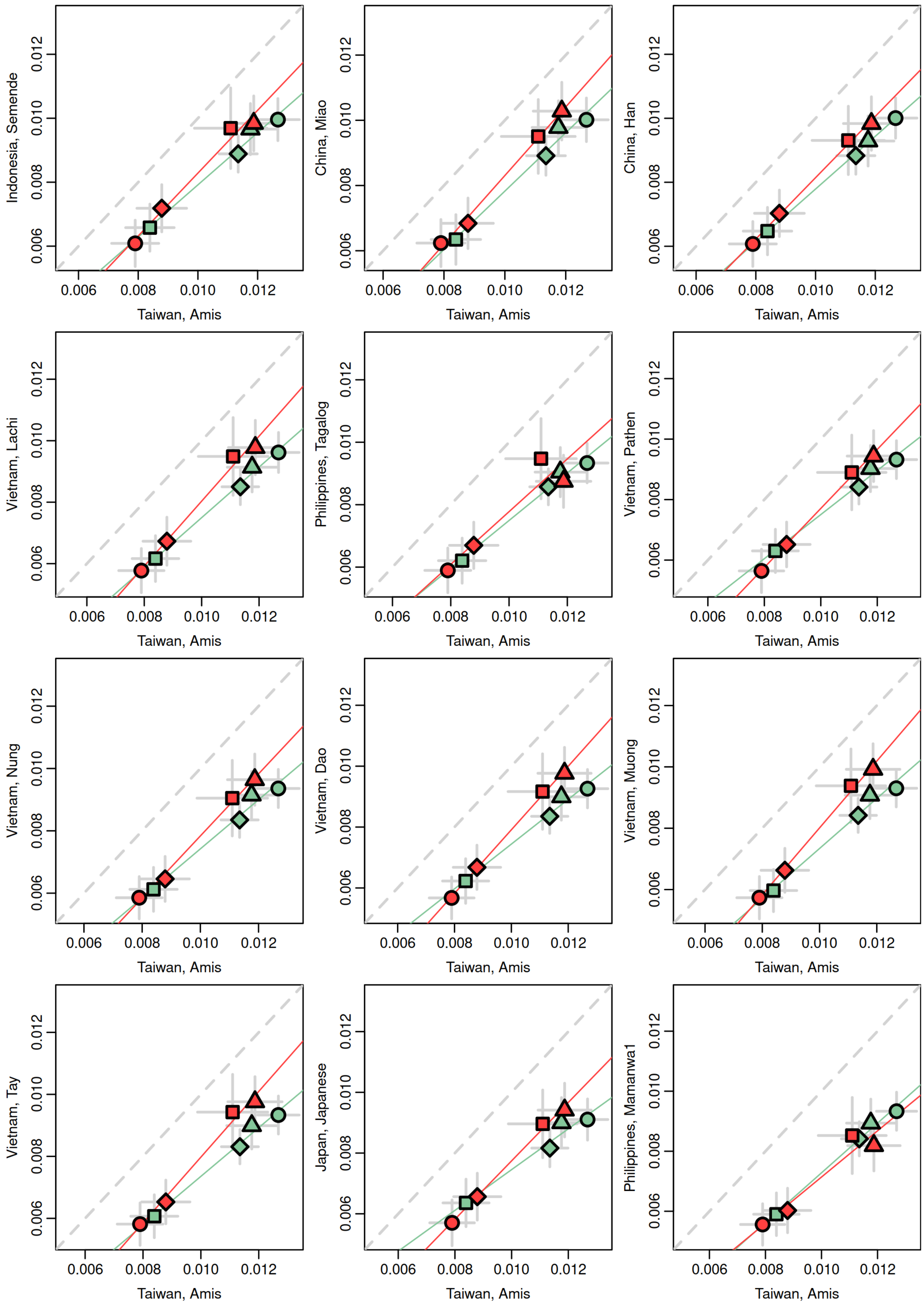

Indonesia, Semende
China, Miao
China, Han
Vietnam, Lachi
Philippines, Tagalog
Vietnam, Pathen
Vietnam, Nung
Vietnam, Dao
Vietnam, Muong
Vietnam, Tay
Japan, Japanese
Philippines, Mamanwa1
Taiwan, Amis
0.006
0.008
0.010
0.012

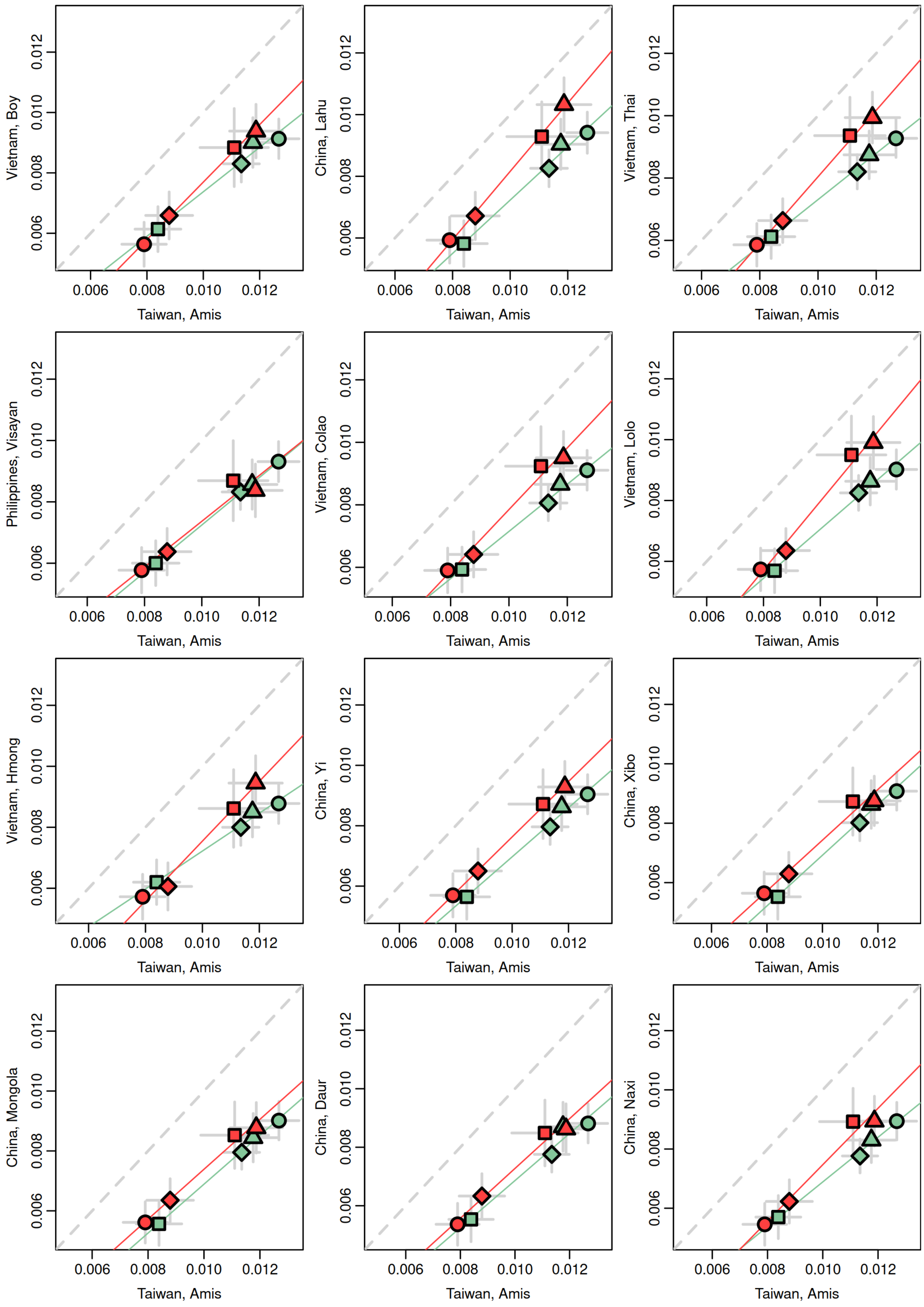
Vietnam, Boy
China, Lahu
Vietnam, Thai
Philippines, Visayan
Vietnam, Colao
Vietnam, Lolo
Vietnam, Hmong
China, Yi
China, Xibo
China, Mongola
China, Daur
China, Naxi
Taiwan, Amis
0.006
0.008
0.010
0.012

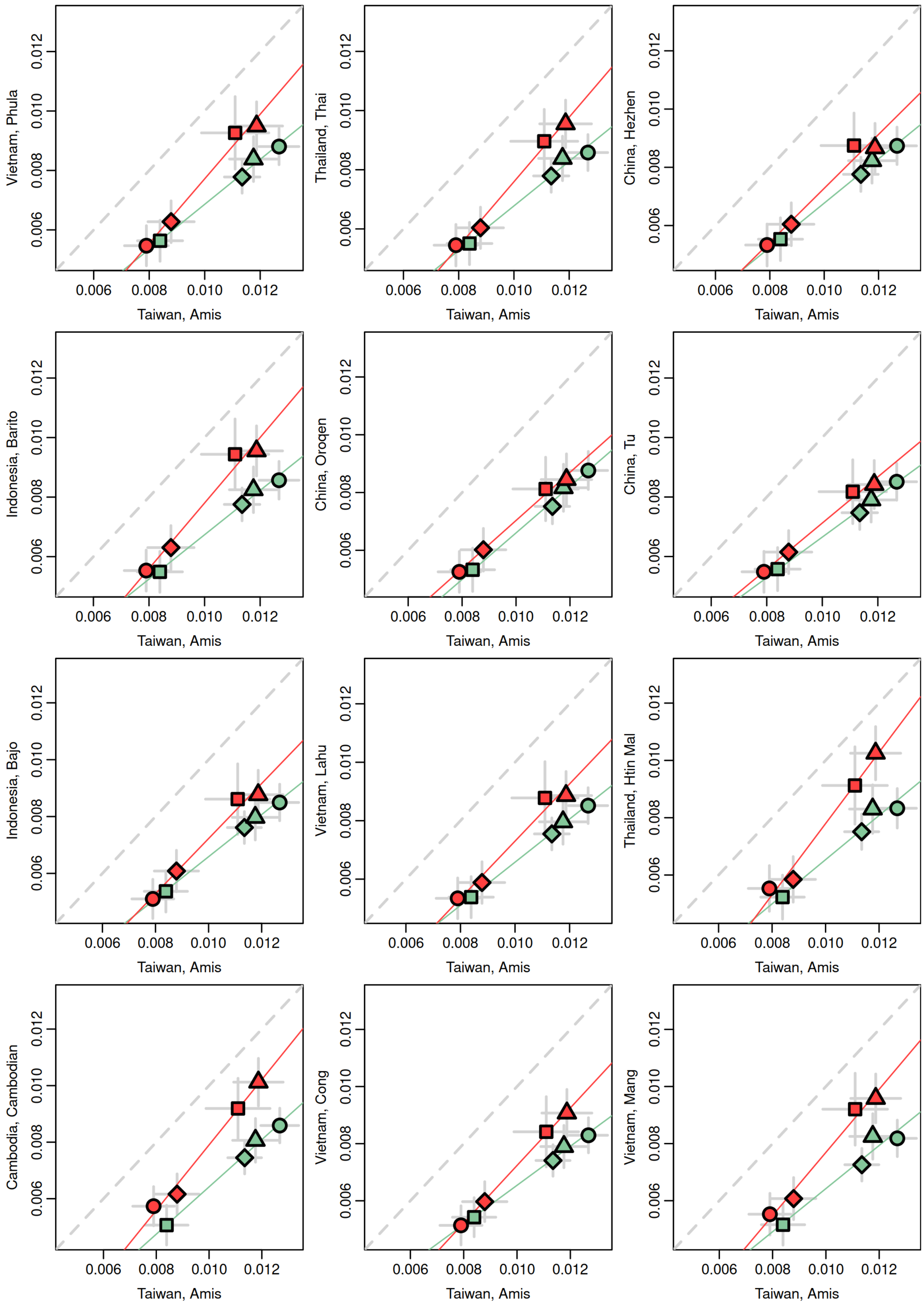
Vietnam, Phula
Thailand, Thai
China, Hezhen
Indonesia, Barito
China, Oroqen
China, Tu
Indonesia, Bajo
Vietnam, Lahu
Thailand, Htin Mal
Cambodia, Cambodian
Vietnam, Cong
Vietnam, Mang
Taiwan, Amis
0.006
0.008
0.010
0.012

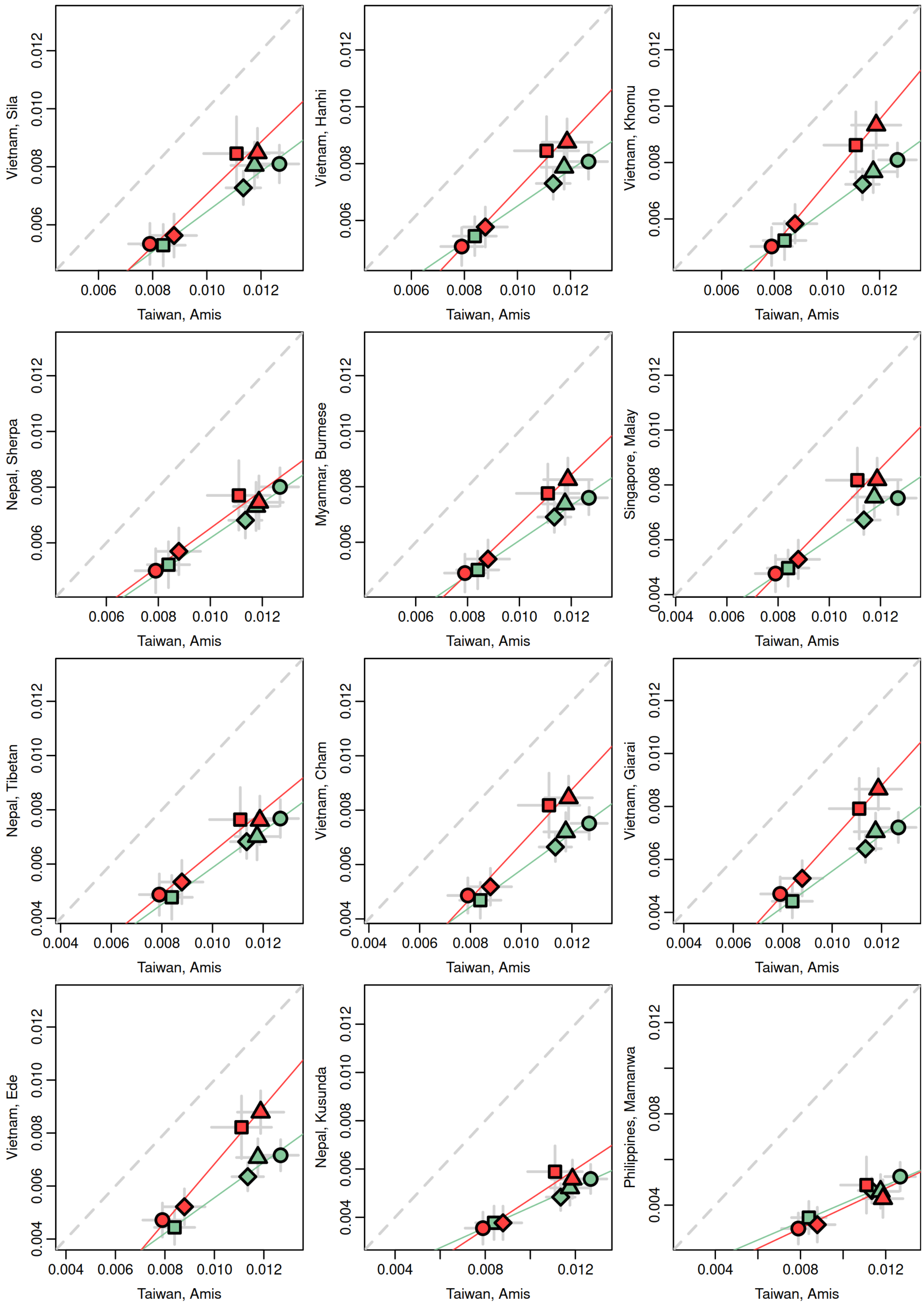
Vietnam, Sila
Taiwan, Amis
Vietnam, Hanhi
Taiwan, Amis
Vietnam, Khomu
Taiwan, Amis
Nepal, Sherpa
Taiwan, Amis
Myanmar, Burmese
Taiwan, Amis
Singapore, Malay
Taiwan, Amis
Nepal, Tibetan
Taiwan, Amis
Vietnam, Cham
Taiwan, Amis
Vietnam, Giarai
Taiwan, Amis
Vietnam, Ede
Taiwan, Amis
Nepal, Kusunda
Taiwan, Amis
Philippines, Mamanwa
Taiwan, Amis
0.004
0.006
0.008
0.010
0.012

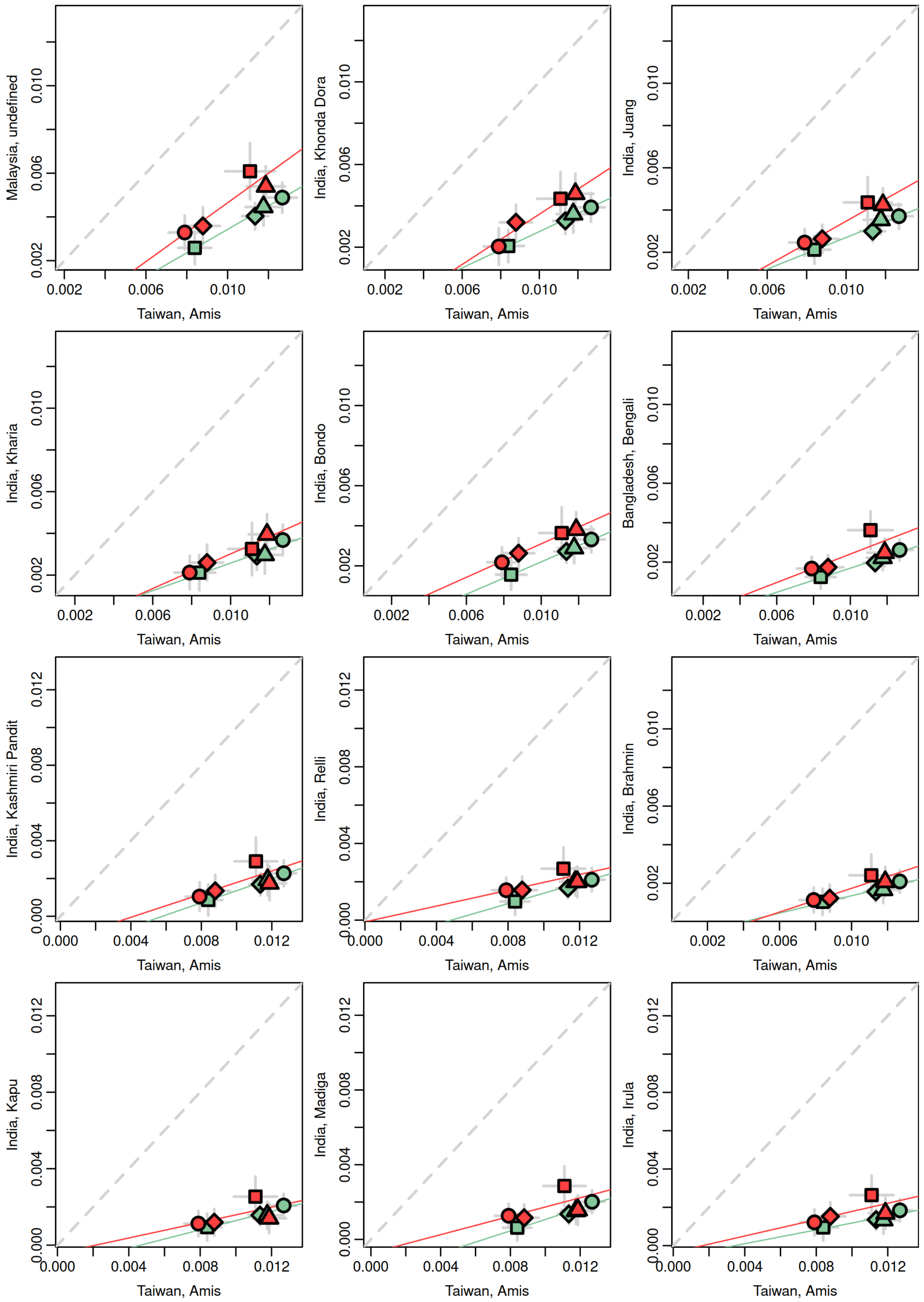
Malaysia, undefined
India, Khonda Dora
India, Juang
India, Kharia
India, Bondo
Bangladesh, Bengali
India, Kashmiri Pandit
India, Relli
India, Brahmin
India, Kapu
India, Madiga
India, Irula
Taiwan, Amis
0.000
0.002
0.004
0.006
0.008
0.010
0.012

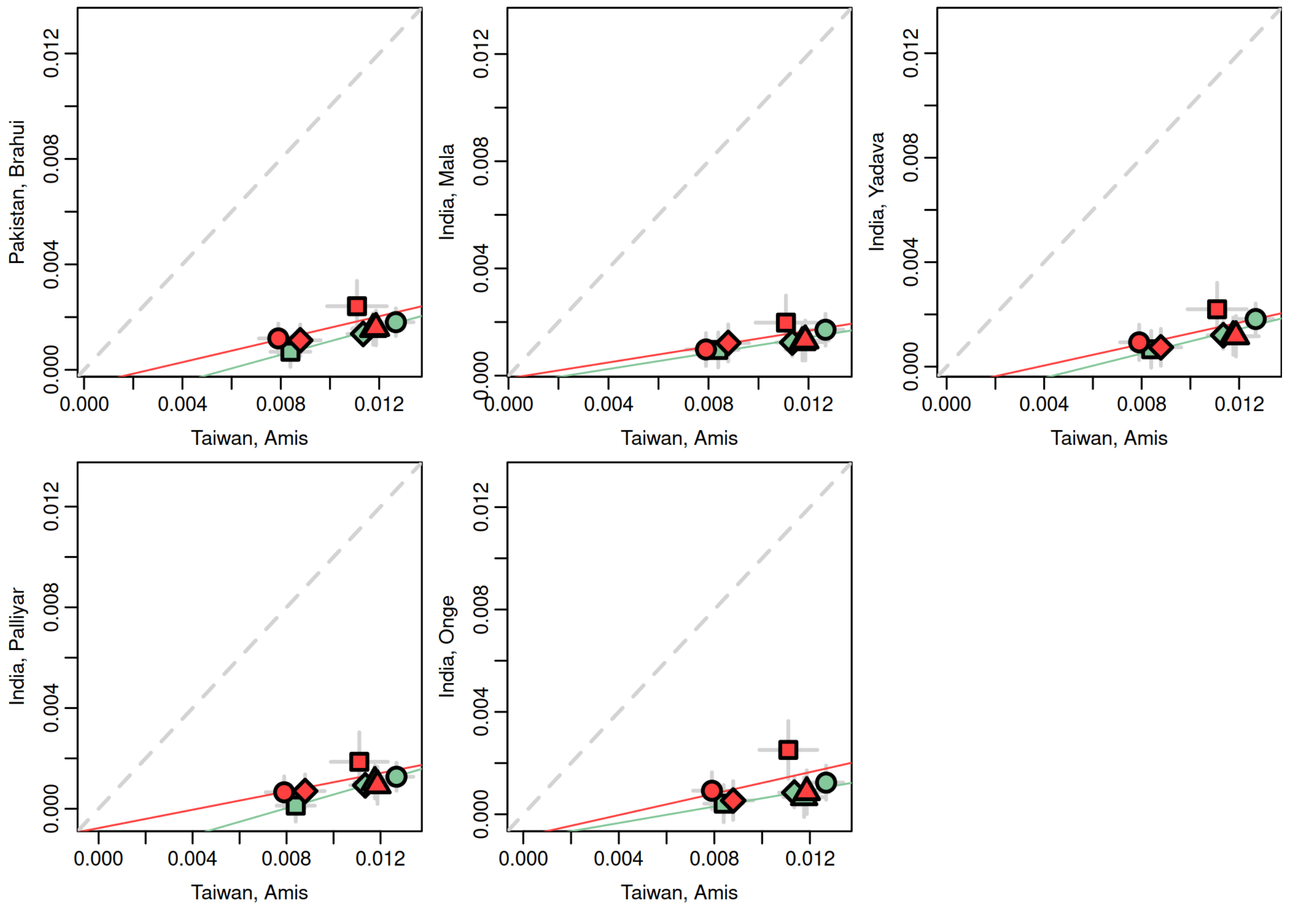
Pakistan, Brahui
India, Mala
India, Yadava
India, Palliyar
India, Onge
Taiwan, Amis
0.000
0.004
0.008
0.012

Supplementary Figure 4 - Biplots showing the results of pairs of f_4-statistics of the form: *f_4(Mbuti, test; New Guinea Highlanders, ancient Wallacea).* The test groups, shown on the x-y axis label, include ancient groups from mainland Asia, Island southeast Asia, and Oceania that have no discernible Papuan-related ancestry based on the DyStruct analysis. Data are presented as exact f_4-values ± 2 SE indicated by grey lines. Linear regression lines for the North Moluccas and East Nusa Tenggara individuals are shown in green and red, respectively.

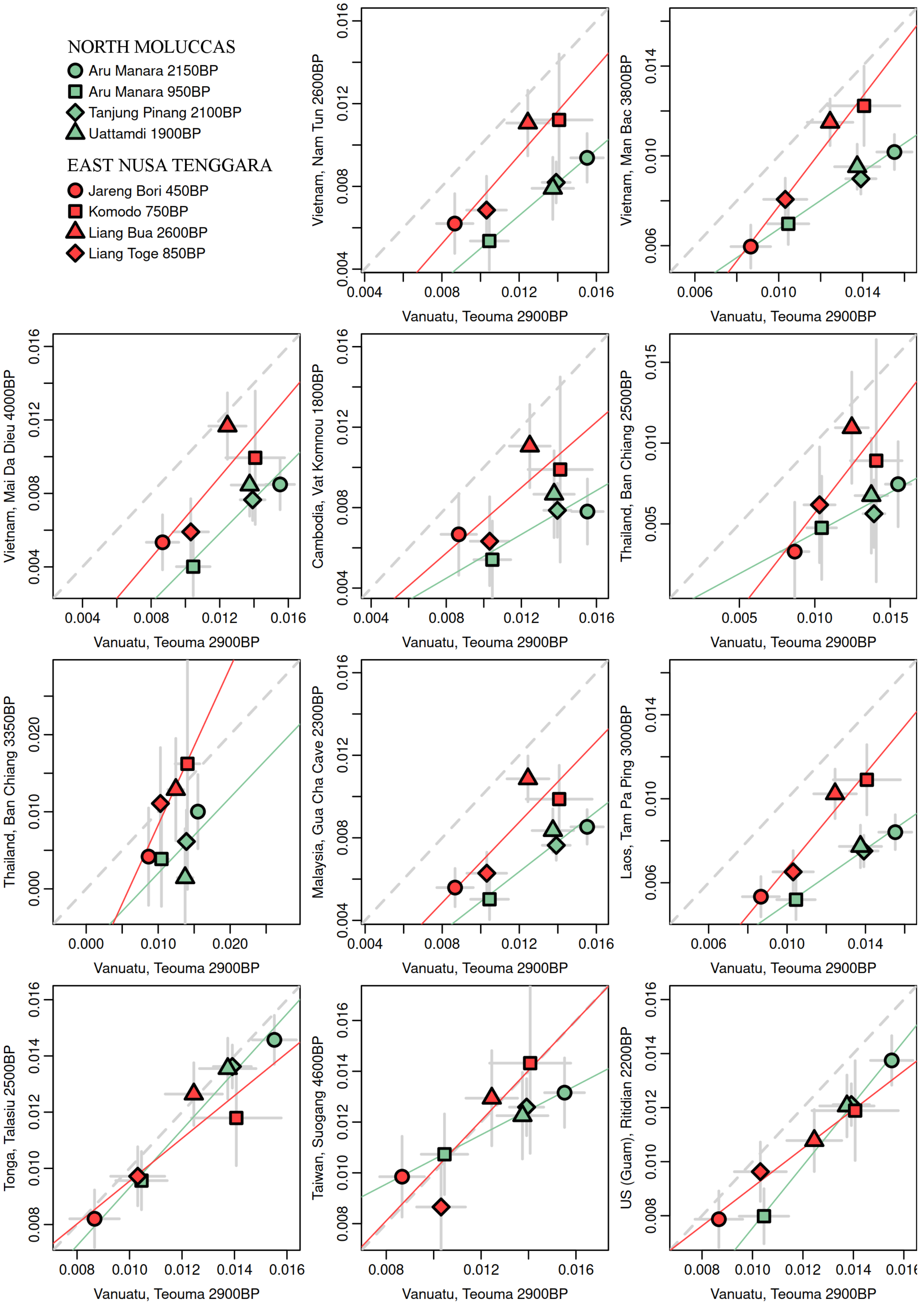

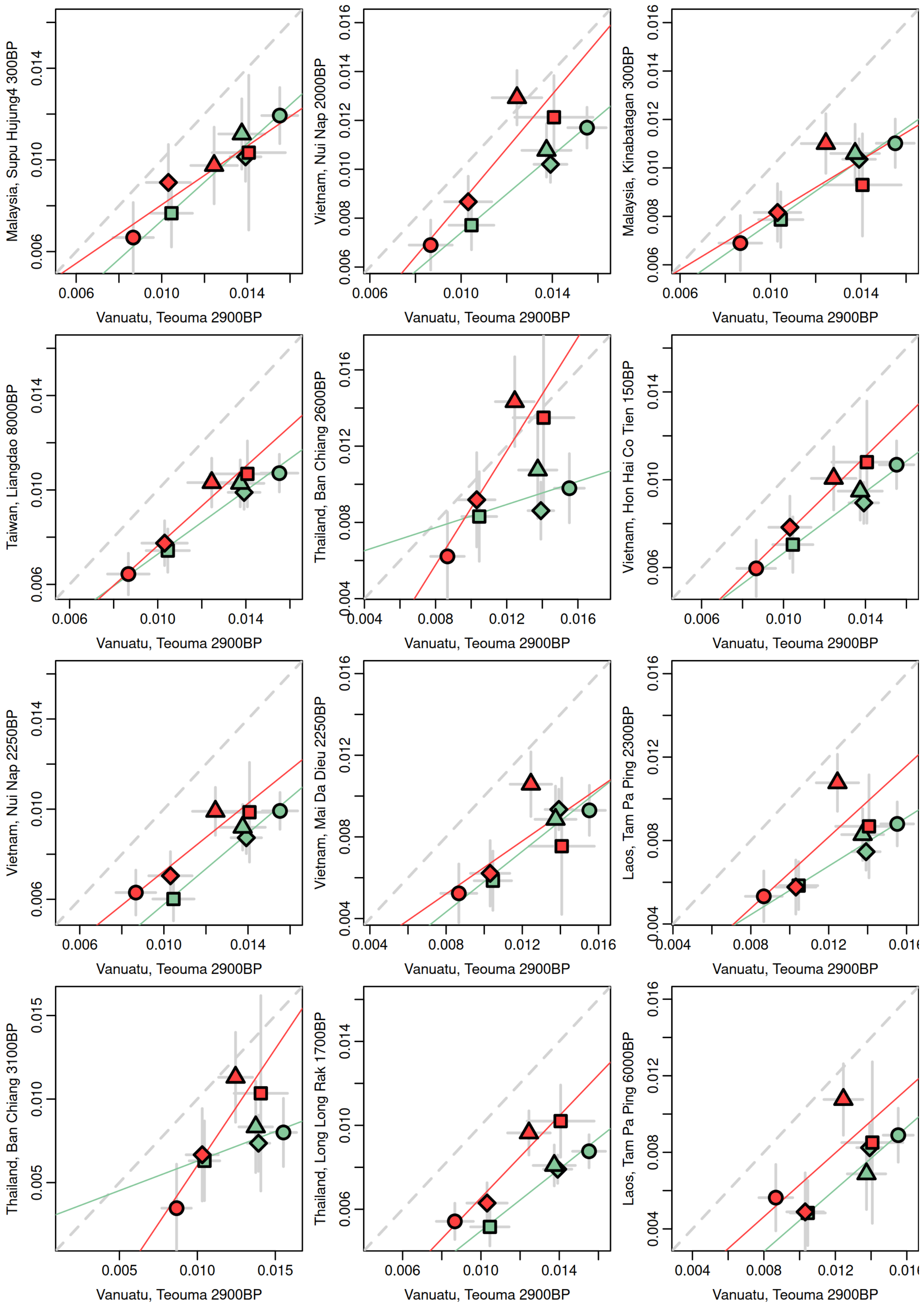

Malaysia, Supu Hujung4 300BP
0.006 0.010 0.014
Vanuatu, Teouma 2900BP
0.006 0.010 0.014
Vietnam, Nui Nap 2000BP
0.006 0.008 0.010 0.012 0.014 0.016
Vanuatu, Teouma 2900BP
0.006 0.010 0.014
Malaysia, Kinabatagan 300BP
0.006 0.008 0.010 0.012 0.014 0.016
Vanuatu, Teouma 2900BP
0.006 0.010 0.014
Taiwan, Liangdao 8000BP
0.006 0.010 0.014
Vanuatu, Teouma 2900BP
0.006 0.010 0.014
Thailand, Ban Chiang 2600BP
0.004 0.008 0.012 0.016
Vanuatu, Teouma 2900BP
0.004 0.008 0.012 0.016
Vietnam, Hon Hai Co Tien 150BP
0.006 0.010 0.014
Vanuatu, Teouma 2900BP
0.006 0.010 0.014
Vietnam, Nui Nap 2250BP
0.006 0.010 0.014
Vanuatu, Teouma 2900BP
0.006 0.010 0.014
Vietnam, Mai Da Dieu 2250BP
0.004 0.008 0.012 0.016
Vanuatu, Teouma 2900BP
0.004 0.008 0.012 0.016
Laos, Tam Pa Ping 2300BP
0.004 0.008 0.012 0.016
Vanuatu, Teouma 2900BP
0.004 0.008 0.012
Thailand, Ban Chiang 3100BP
0.005 0.010 0.015
Vanuatu, Teouma 2900BP
0.005 0.010 0.015
Thailand, Long Long Rak 1700BP
0.006 0.010 0.014
Vanuatu, Teouma 2900BP
0.006 0.010 0.014
Laos, Tam Pa Ping 6000BP
0.004 0.008 0.012 0.016
Vanuatu, Teouma 2900BP
0.004 0.008 0.012

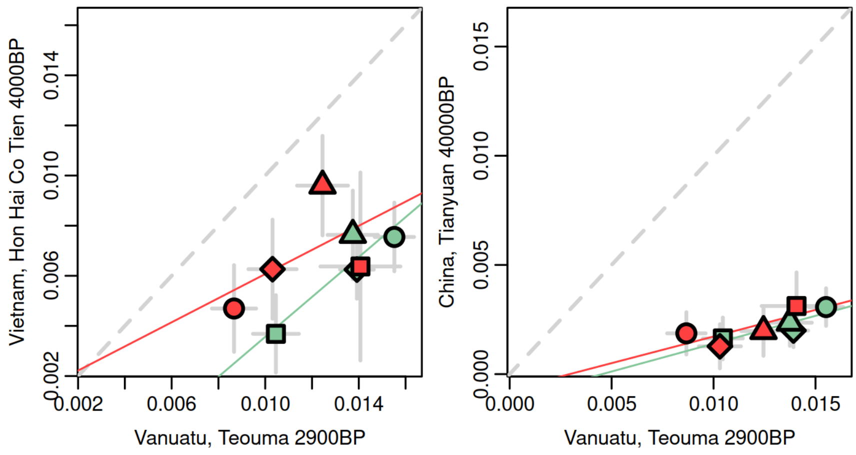

Vietnam, Hon Hai Co Tien 4000BP
0.002
0.006
0.010
0.014
0.002
0.006
0.010
0.014
Vanuatu, Teouma 2900BP
China, Tianyuan 40000BP
0.000
0.005
0.010
0.015
0.000
0.005
0.010
0.015
Vanuatu, Teouma 2900BP

Supplementary Figure 5 - Bayesian linear regression for pairs of f_4-statistics of the form: f_4*(Mbuti, test; New Guinea Highlanders, ancient Wallacea)*, where *test* corresponds to the present-day groups shown in the x-y axis labels. The lines show the posterior means for the North Moluccas (green) and East Nusa Tenggara (red) groups, while the shades show the 95% credible interval. Filled symbols show the observed values (standardized) and non-filled symbols show their posterior means, with the lines connecting pairs of points from the same ancient sample.

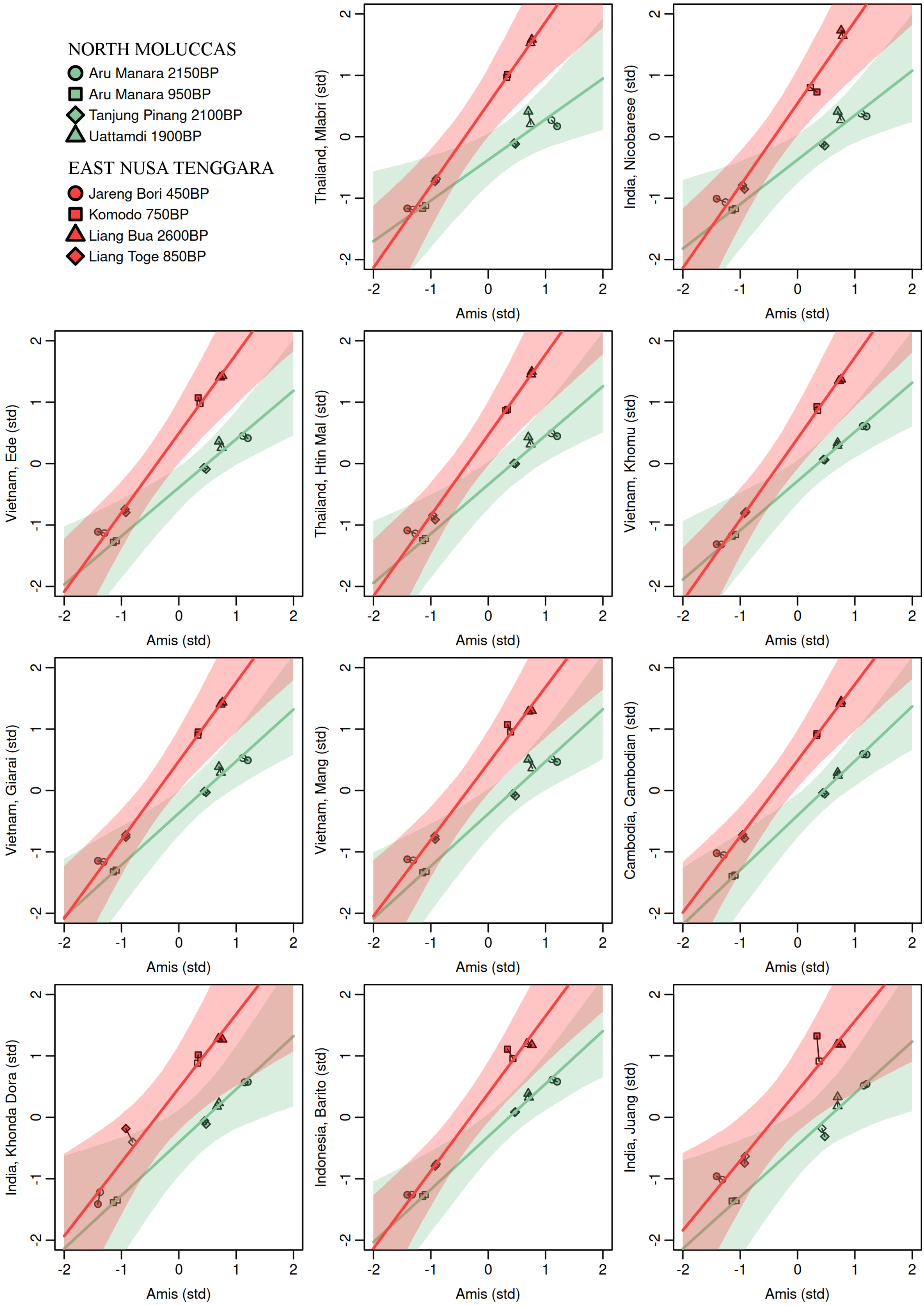

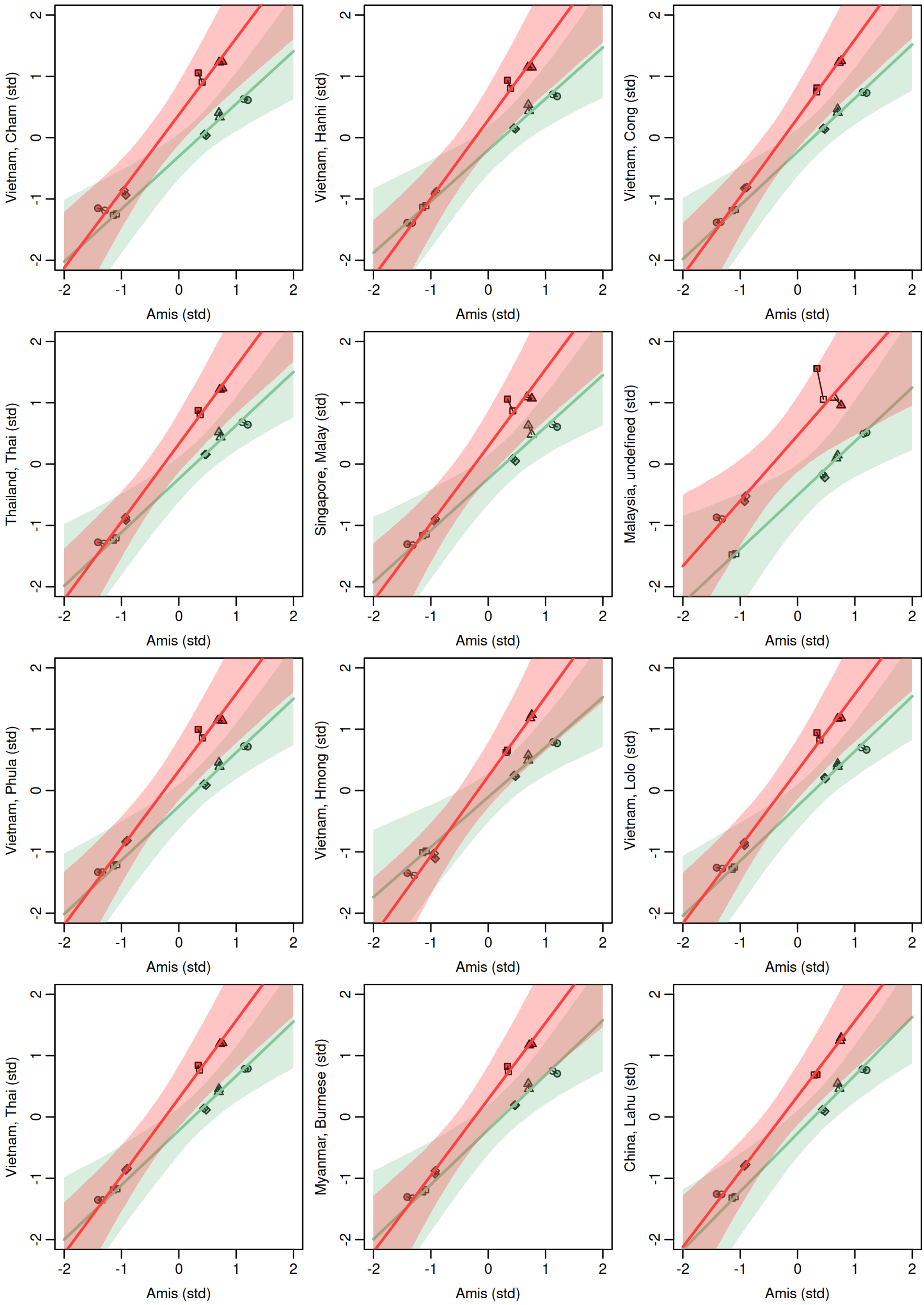
Vietnam, Cham (std)
Vietnam, Hanhi (std)
Vietnam, Cong (std)
Thailand, Thai (std)
Singapore, Malay (std)
Malaysia, undefined (std)
Vietnam, Phula (std)
Vietnam, Hmong (std)
Vietnam, Lolo (std)
Vietnam, Thai (std)
Myanmar, Burmese (std)
China, Lahu (std)
Amis (std)

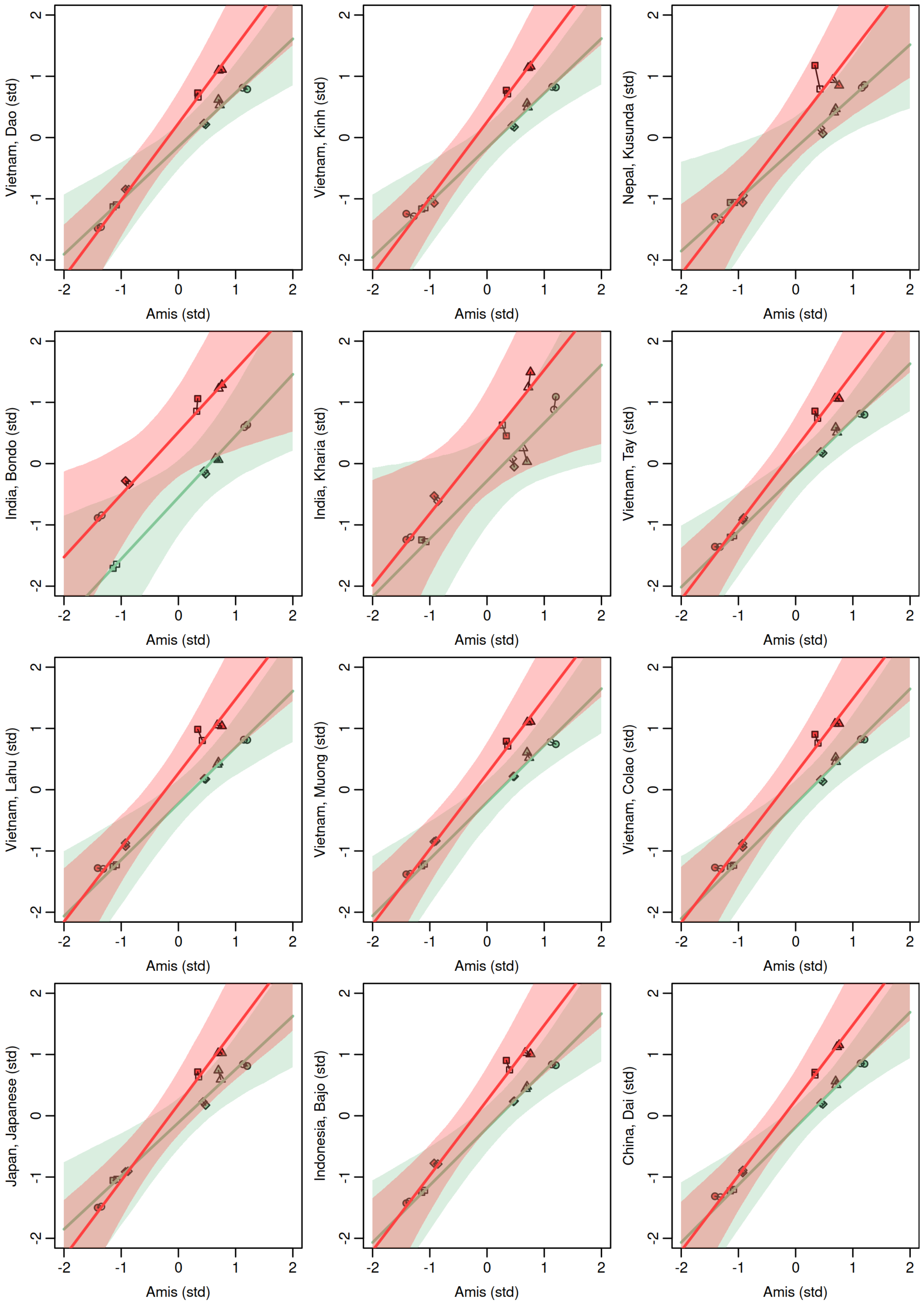

Vietnam, Dao (std)
Vietnam, Kinh (std)
Nepal, Kusunda (std)
India, Bondo (std)
India, Kharia (std)
Vietnam, Tay (std)
Vietnam, Lahu (std)
Vietnam, Muong (std)
Vietnam, Colao (std)
Japan, Japanese (std)
Indonesia, Bajo (std)
China, Dai (std)
Amis (std)
-2
-1
0
1
2

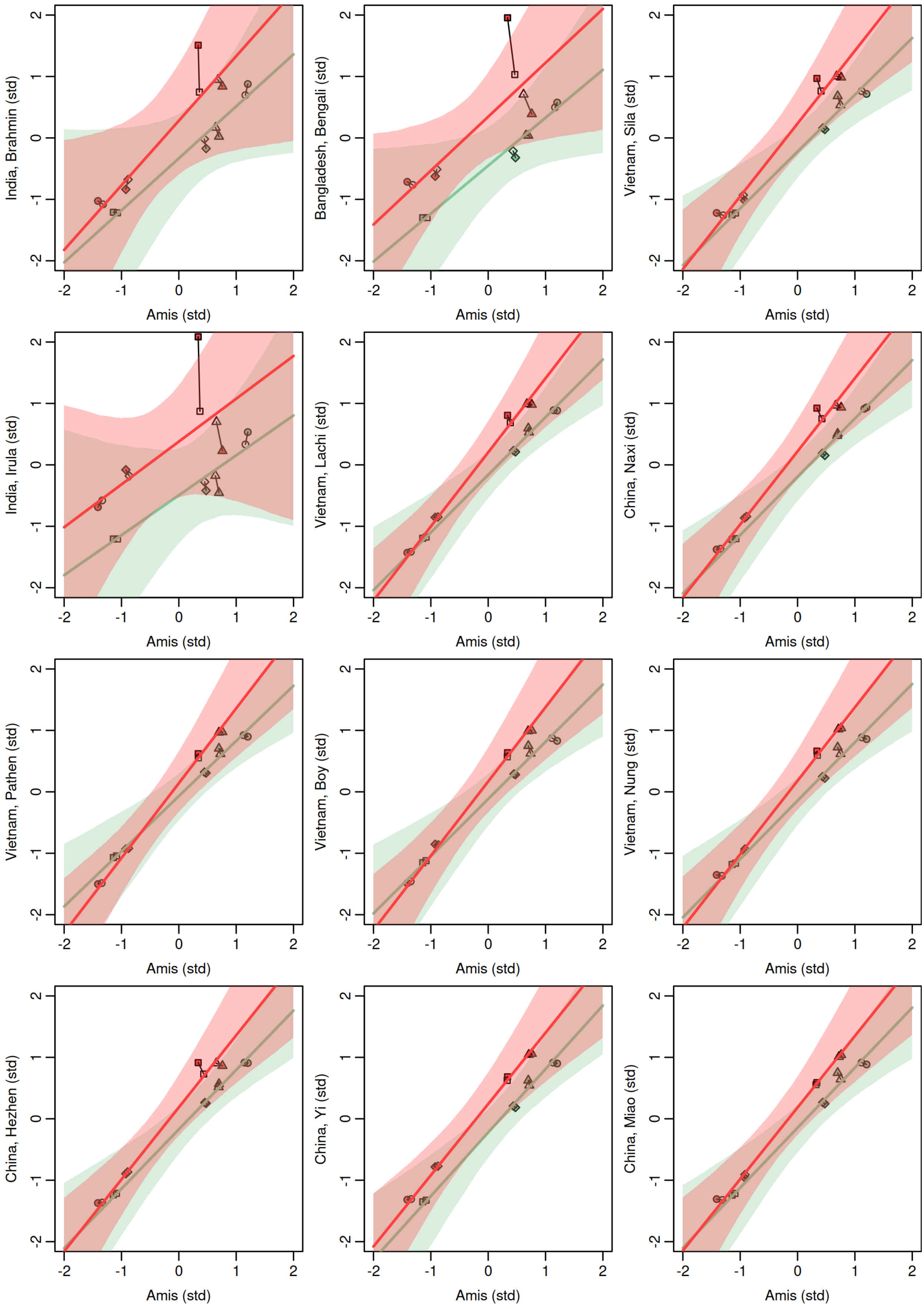
India, Brahmin (std)
Bangladesh, Bengali (std)
Vietnam, Sila (std)
India, Irula (std)
Vietnam, Lachi (std)
China, Naxi (std)
Vietnam, Pathen (std)
Vietnam, Boy (std)
Vietnam, Nung (std)
China, Hezhen (std)
China, Yi (std)
China, Miao (std)
Amis (std)

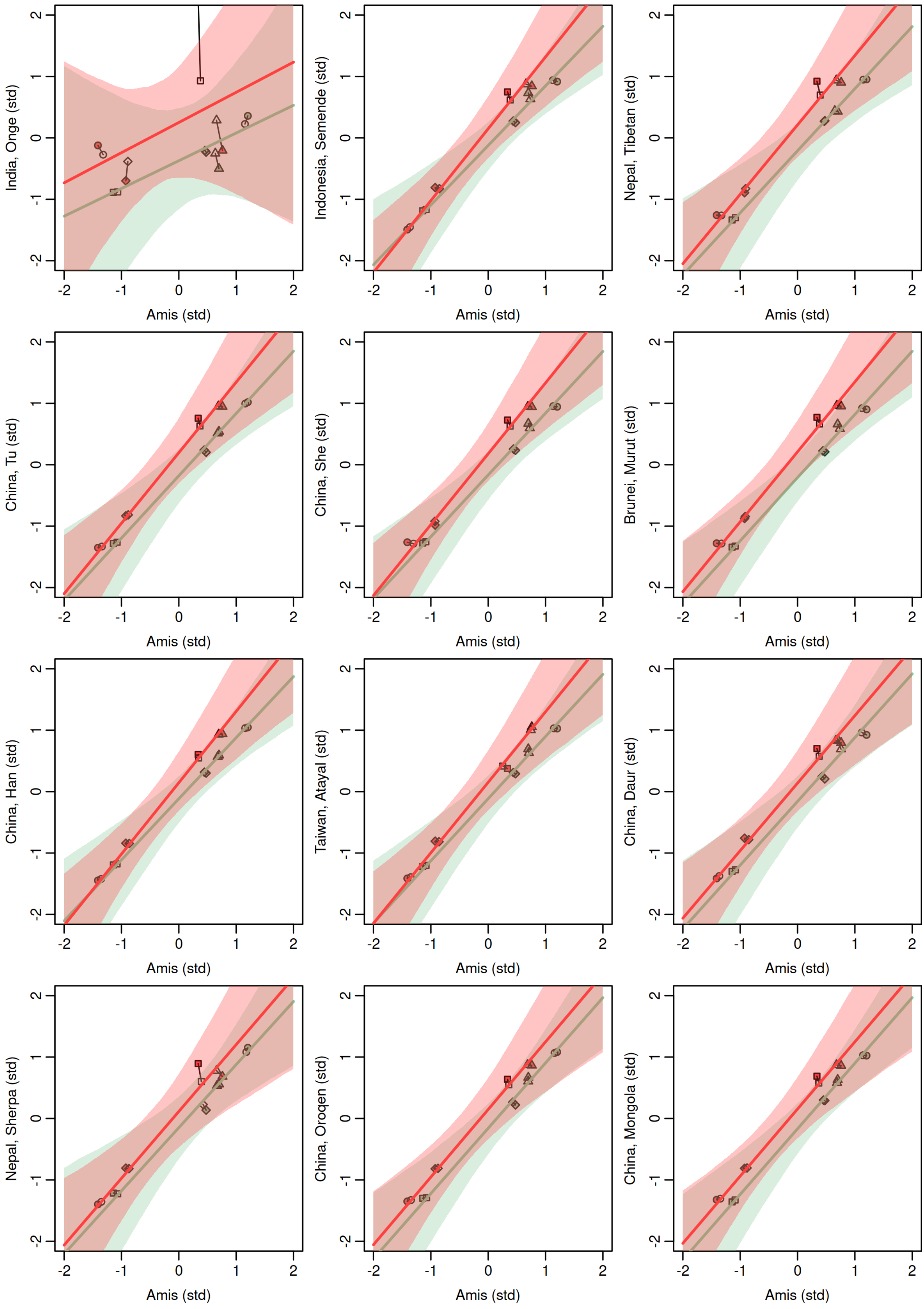
India, Onge (std)
Indonesia, Semende (std)
Nepal, Tibetan (std)
China, Tu (std)
China, She (std)
Brunei, Murut (std)
China, Han (std)
Taiwan, Atayal (std)
China, Daur (std)
Nepal, Sherpa (std)
China, Oroqen (std)
China, Mongola (std)
Amis (std)

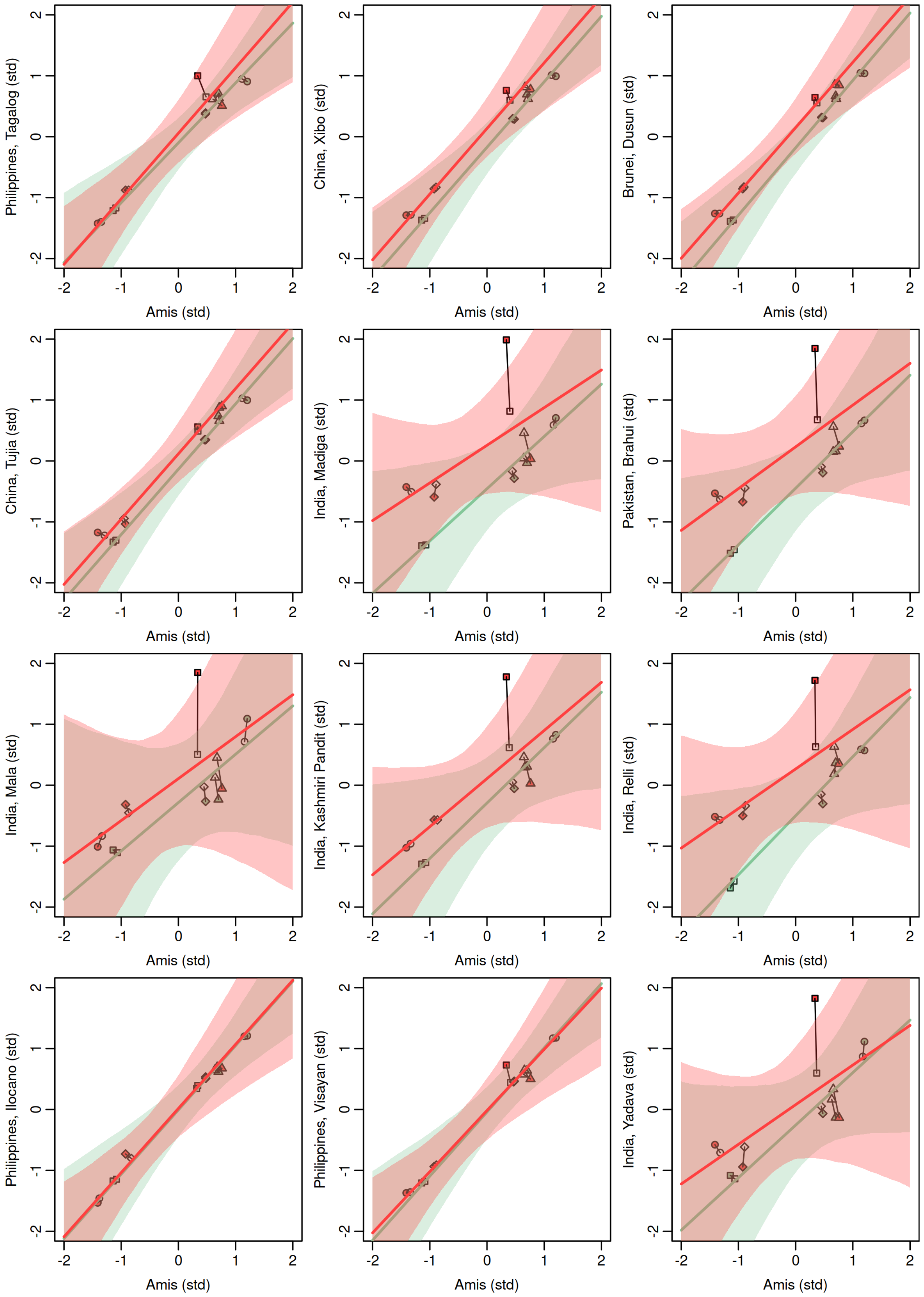
Philippines, Tagalog (std)
China, Xibo (std)
Brunei, Dusun (std)
China, Tujia (std)
India, Madiga (std)
Pakistan, Brahui (std)
India, Mala (std)
India, Kashmiri Pandit (std)
India, Relli (std)
Philippines, Ilocano (std)
Philippines, Visayan (std)
India, Yadava (std)
Amis (std)

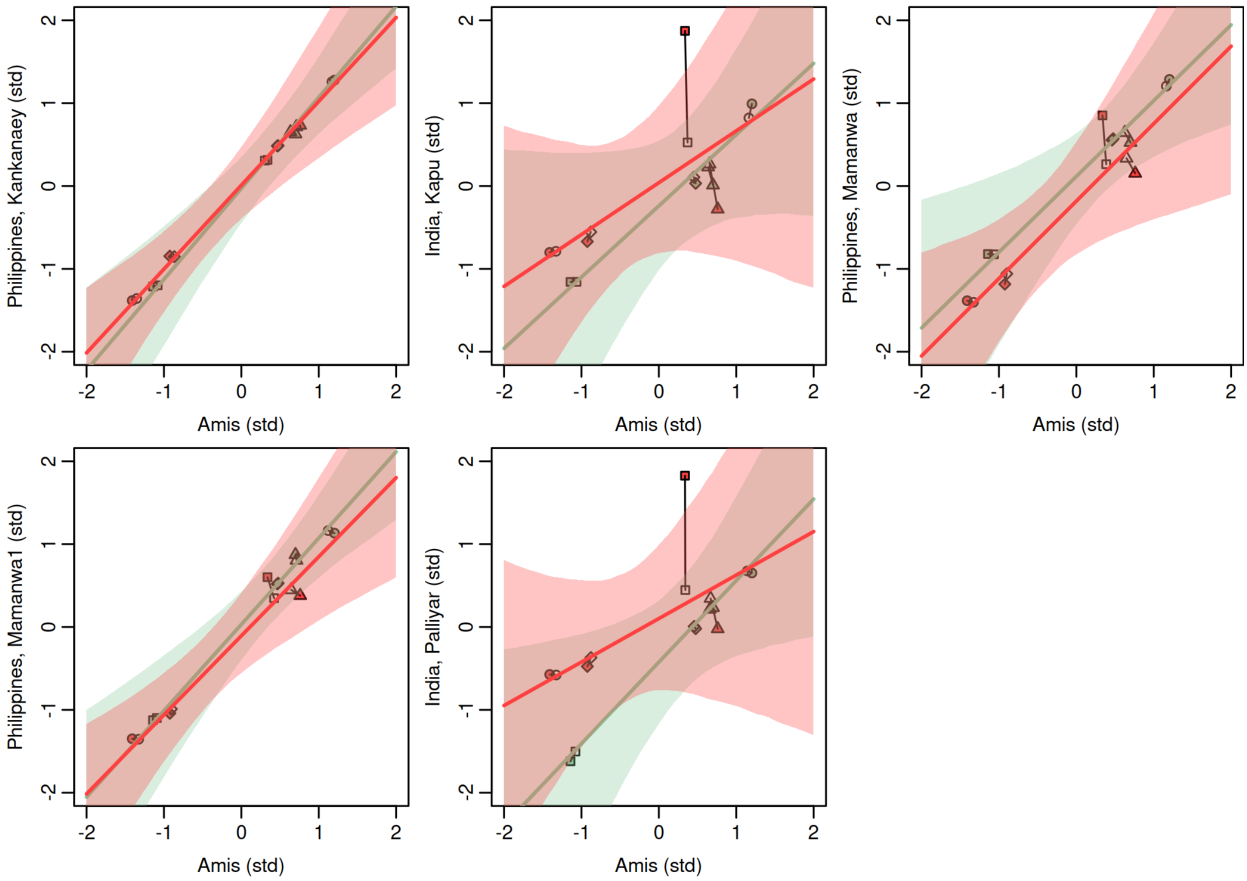
Philippines, Kankanaey (std)
Amis (std)
India, Kapu (std)
Amis (std)
Philippines, Mamanwa (std)
Amis (std)
Philippines, Mamanwa1 (std)
Amis (std)
India, Paliyar (std)
Amis (std)

Supplementary Figure 6 - Bayesian linear regression for pairs of f_4-statistics of the form: f_4*(Mbuti, test; New Guinea Highlanders, ancient Wallacea)*, where *test* corresponds to the ancient groups shown in the x-y axis labels. The lines show the posterior means for the North Moluccas (green) and East Nusa Tenggara (red) groups, while the shades show the 95% credible interval. Filled symbols show the observed values (standardized) and non-filled symbols show their posterior means, with the lines connecting pairs of points from the same ancient sample.

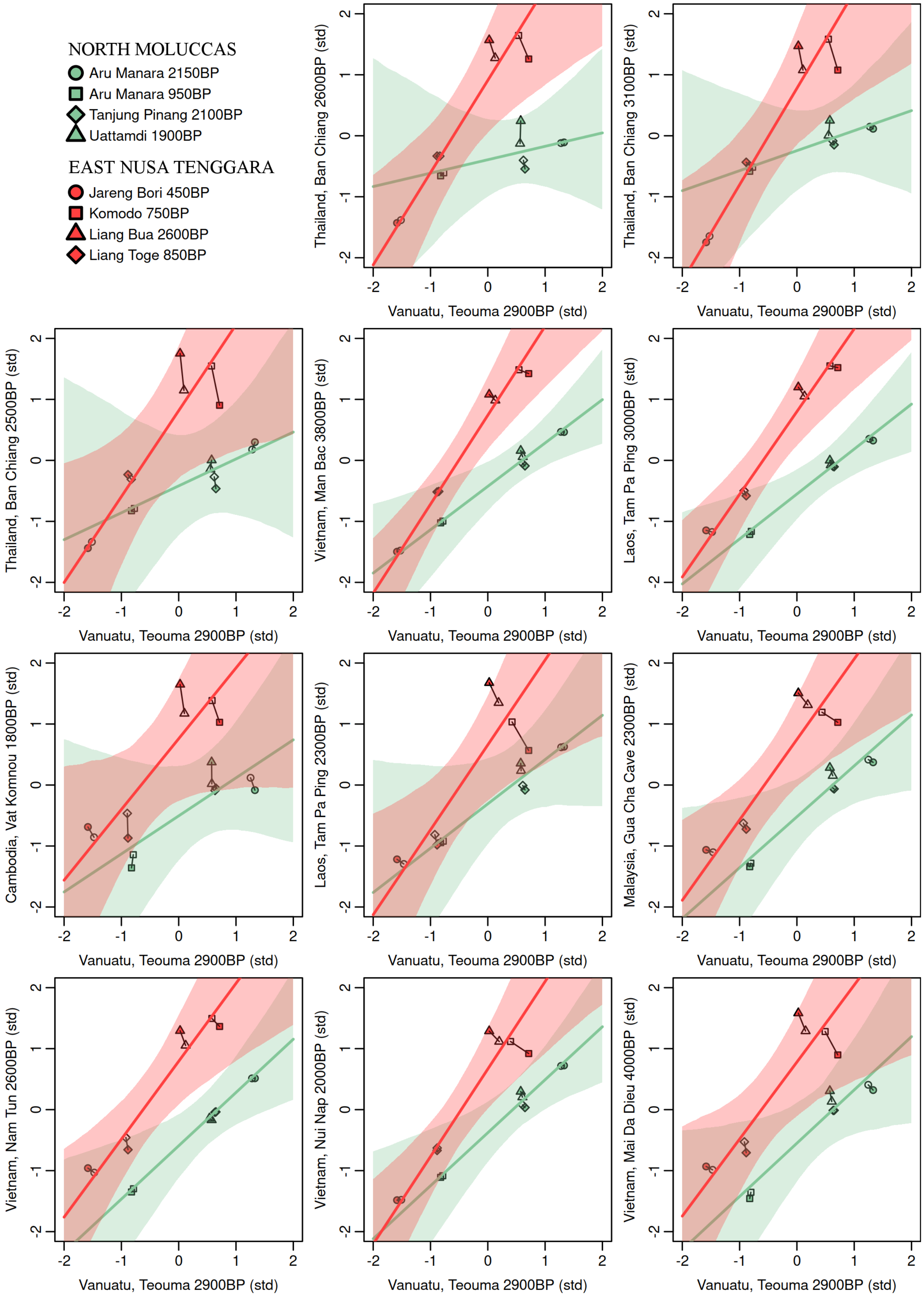

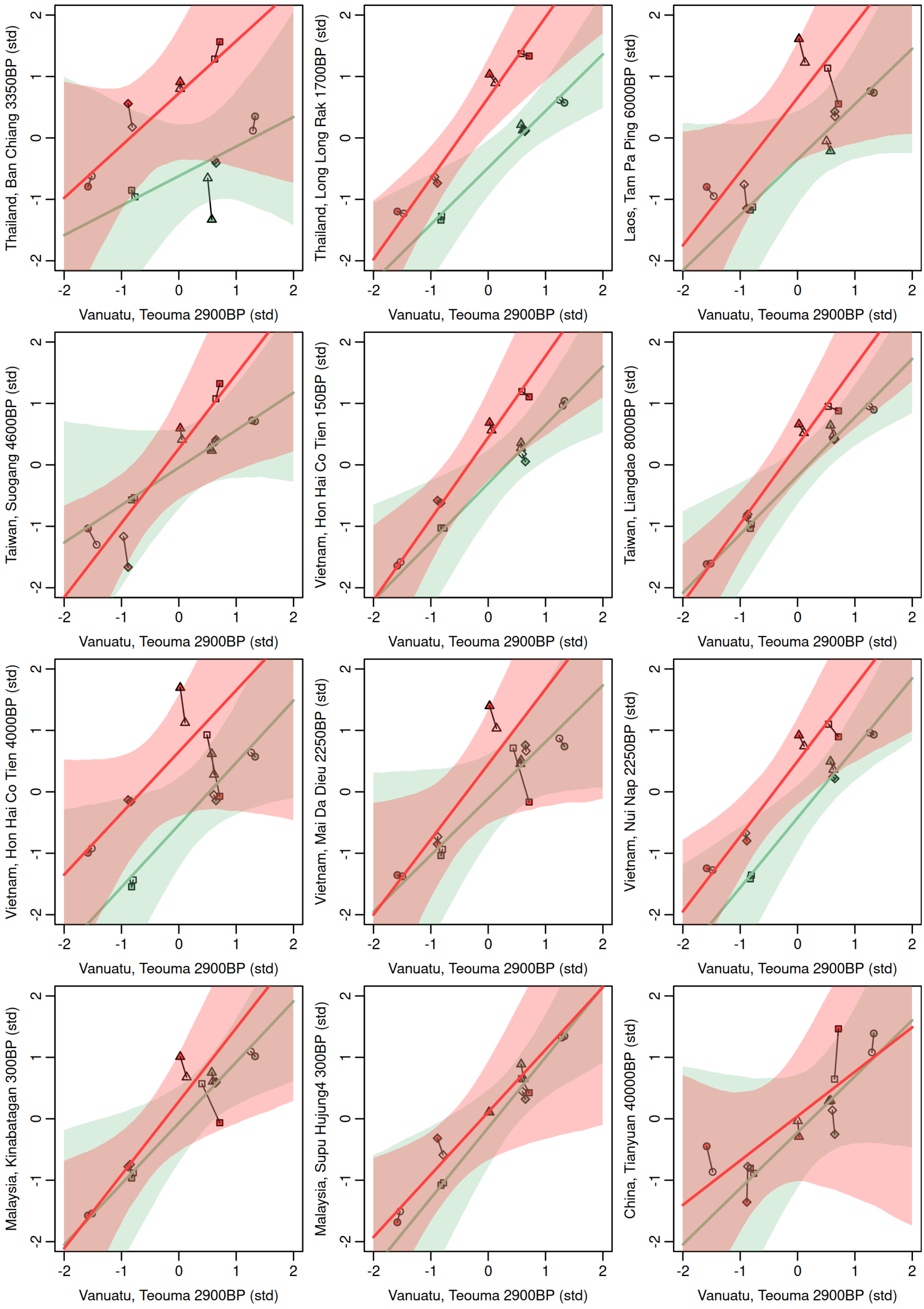
Thailand, Ban Chiang 3350BP (std)
Thailand, Long Long Rak 1700BP (std)
Laos, Tam Pa Ping 6000BP (std)
Taiwan, Suogang 4600BP (std)
Vietnam, Hon Hai Co Tien 150BP (std)
Taiwan, Liangdao 8000BP (std)
Vietnam, Hon Hai Co Tien 4000BP (std)
Vietnam, Mai Da Dieu 2250BP (std)
Vietnam, Nui Nap 2250BP (std)
Malaysia, Kinabatagan 300BP (std)
Malaysia, Supu Hujung4 300BP (std)
China, Tianyuan 40000BP (std)
Vanuatu, Teouma 2900BP (std)
-2
-1
0
1
2

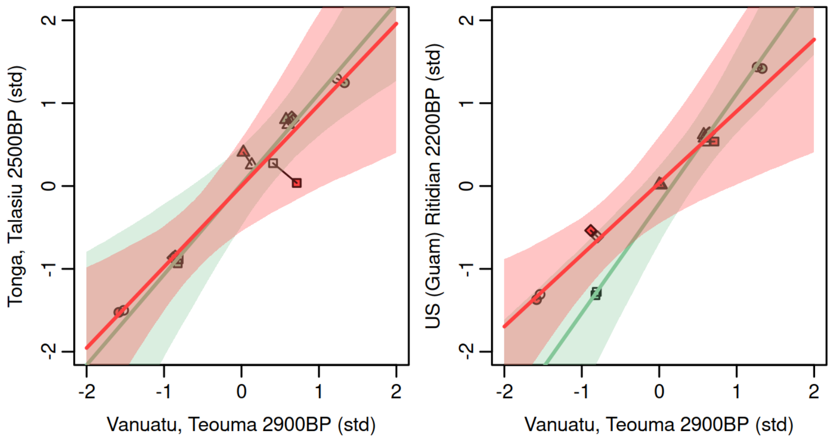

Tonga, Talasiu 2500BP (std)
Vanuatu, Teouma 2900BP (std)
US (Guam) Ritidian 2200BP (std)
Vanuatu, Teouma 2900BP (std)

Supplementary Figure 7 - Differences between the posterior distribution of the regression models for the East Nusa Tenggara and North Moluccas (contrasts). Each plot shows the contrasts for the pair of present-day groups shown in the x-y axis labels. The black line represents differences in the posterior means and the shades show the differences in the credible intervals.

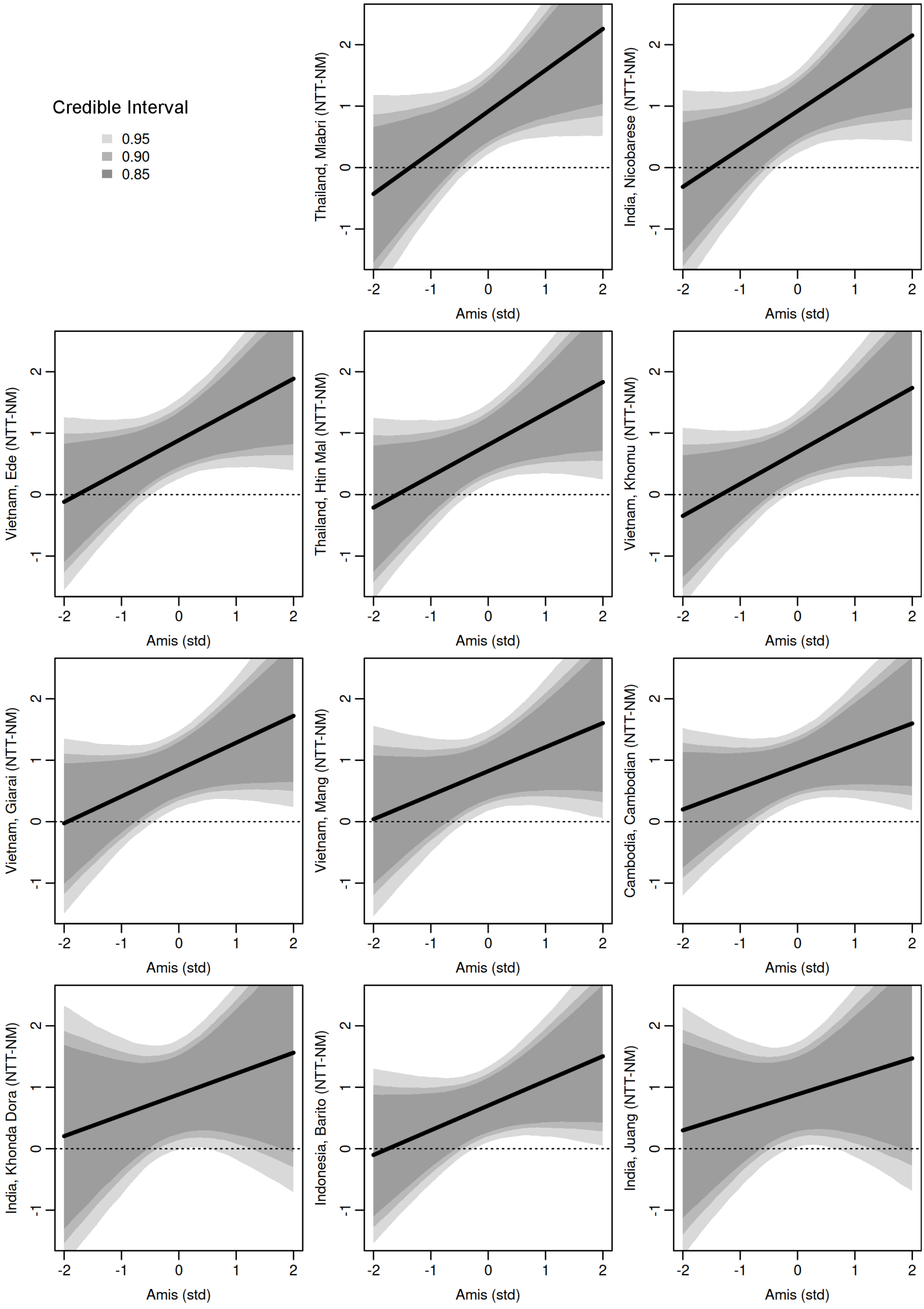

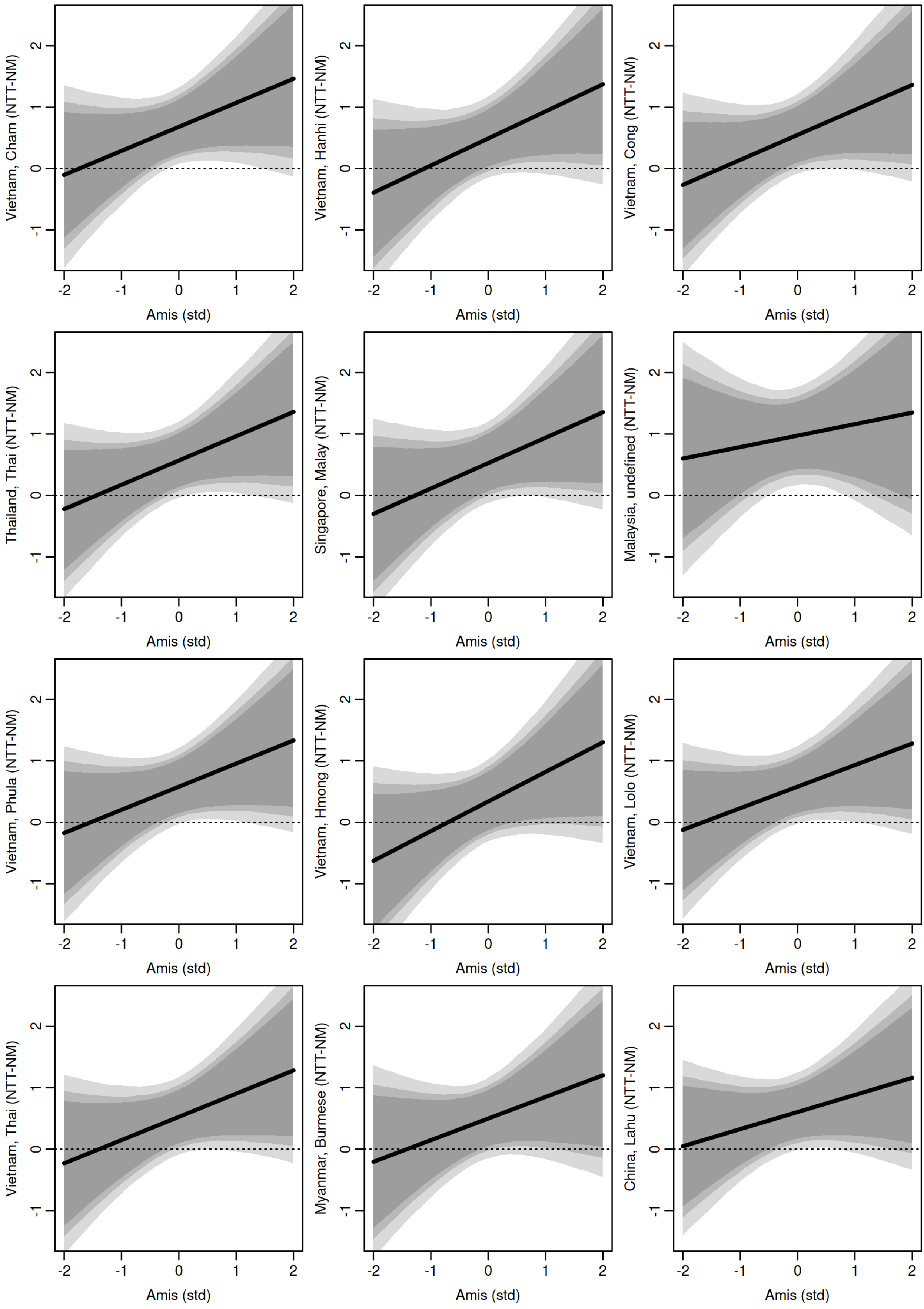
Vietnam, Cham (NTT-NM)
Vietnam, Hanhi (NTT-NM)
Vietnam, Cong (NTT-NM)
Thailand, Thai (NTT-NM)
Singapore, Malay (NTT-NM)
Malaysia, undefined (NTT-NM)
Vietnam, Phula (NTT-NM)
Vietnam, Hmong (NTT-NM)
Vietnam, Lolo (NTT-NM)
Vietnam, Thai (NTT-NM)
Myanmar, Burmese (NTT-NM)
China, Lahu (NTT-NM)
Amis (std)

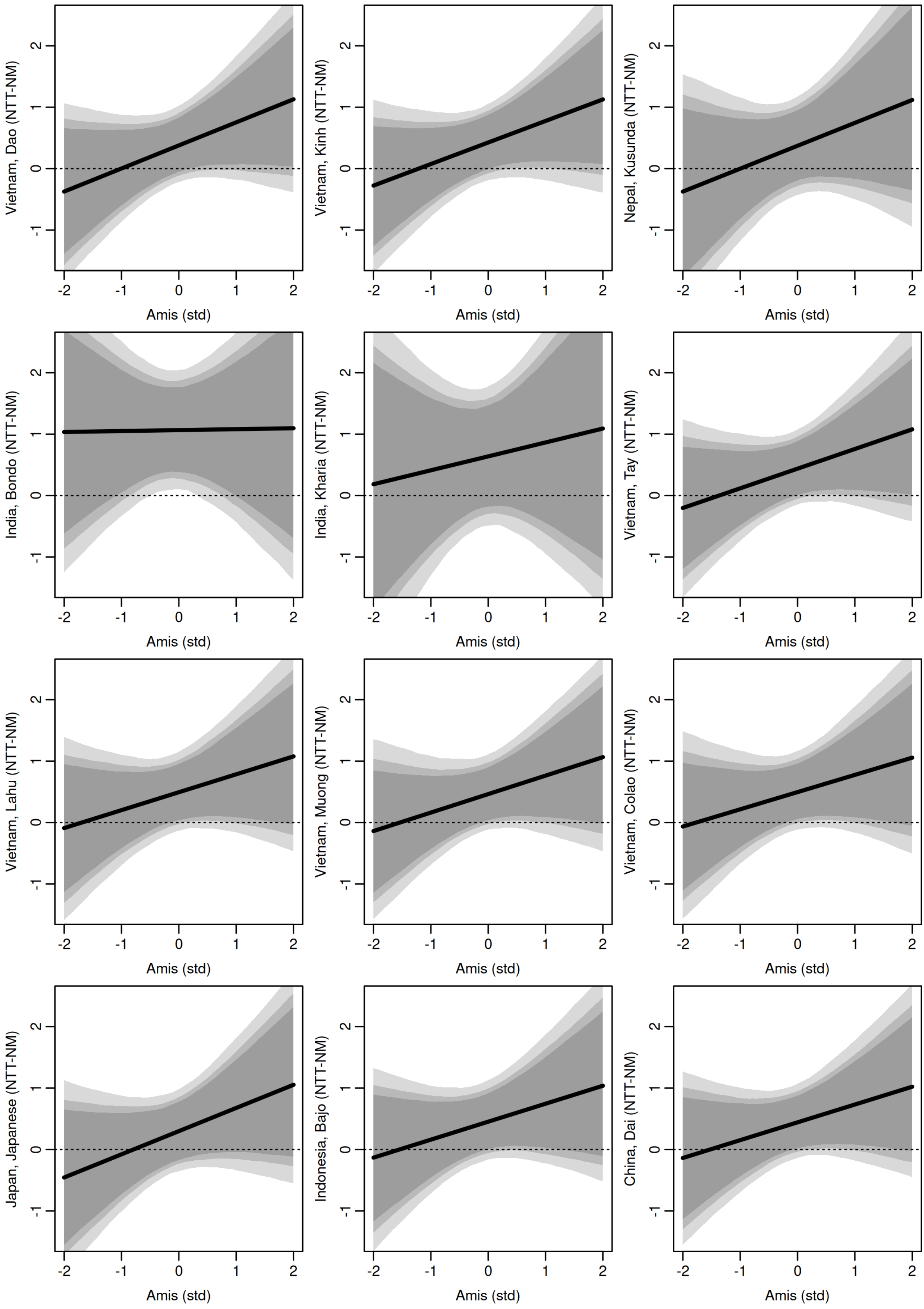
Vietnam, Dao (NTT-NM)
Vietnam, Kinh (NTT-NM)
Nepal, Kusunda (NTT-NM)
India, Bondo (NTT-NM)
India, Kharia (NTT-NM)
Vietnam, Tay (NTT-NM)
Vietnam, Lahu (NTT-NM)
Vietnam, Muong (NTT-NM)
Vietnam, Colao (NTT-NM)
Japan, Japanese (NTT-NM)
Indonesia, Bajo (NTT-NM)
China, Dai (NTT-NM)
Amis (std)
-2
-1
0
1
2

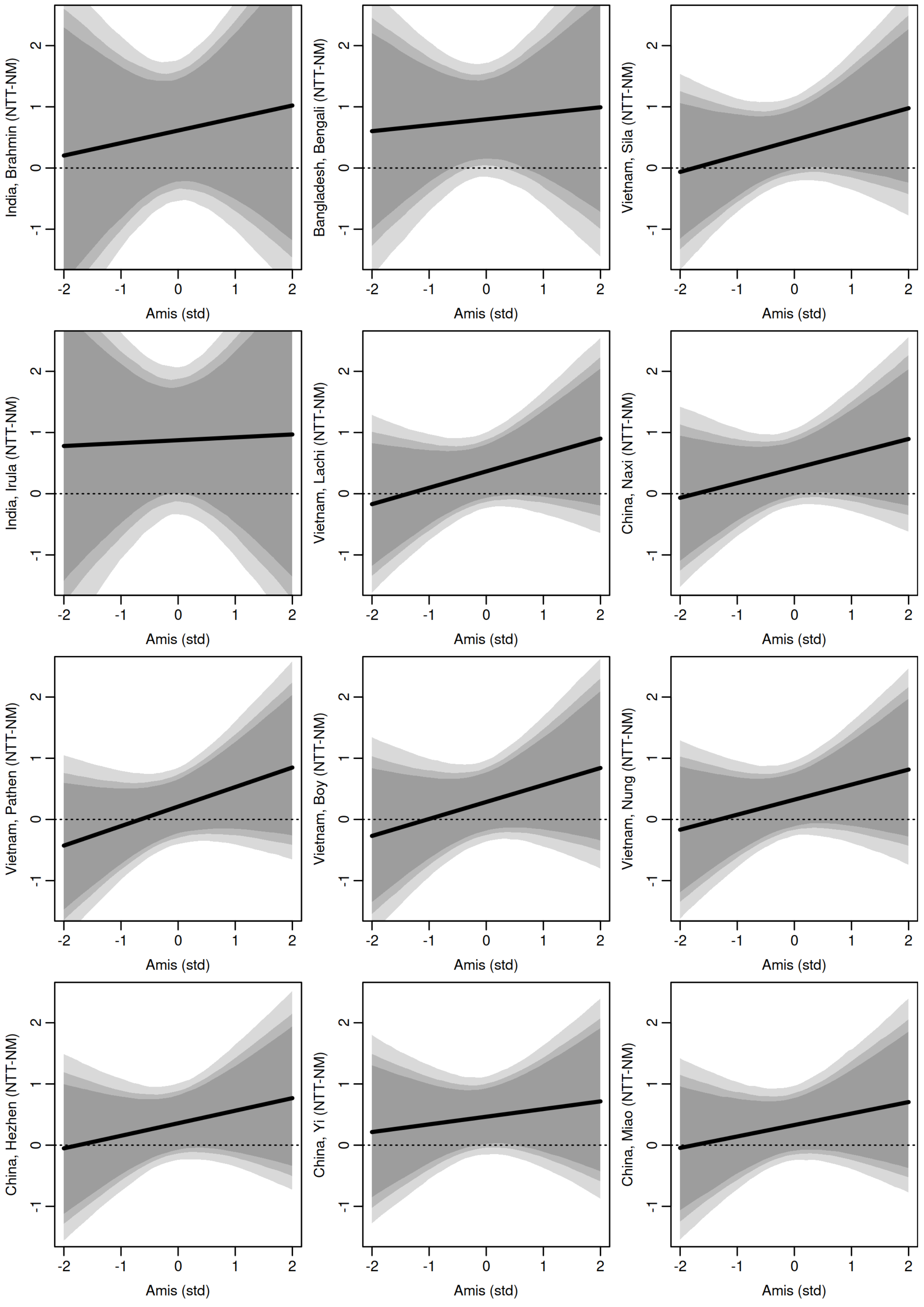
India, Brahmin (NTT-NM)
Bangladesh, Bengali (NTT-NM)
Vietnam, Sila (NTT-NM)
India, Irula (NTT-NM)
Vietnam, Lachi (NTT-NM)
China, Naxi (NTT-NM)
Vietnam, Pathen (NTT-NM)
Vietnam, Boy (NTT-NM)
Vietnam, Nung (NTT-NM)
China, Hezhen (NTT-NM)
China, Yi (NTT-NM)
China, Miao (NTT-NM)
Amis (std)
-2
-1
0
1
2

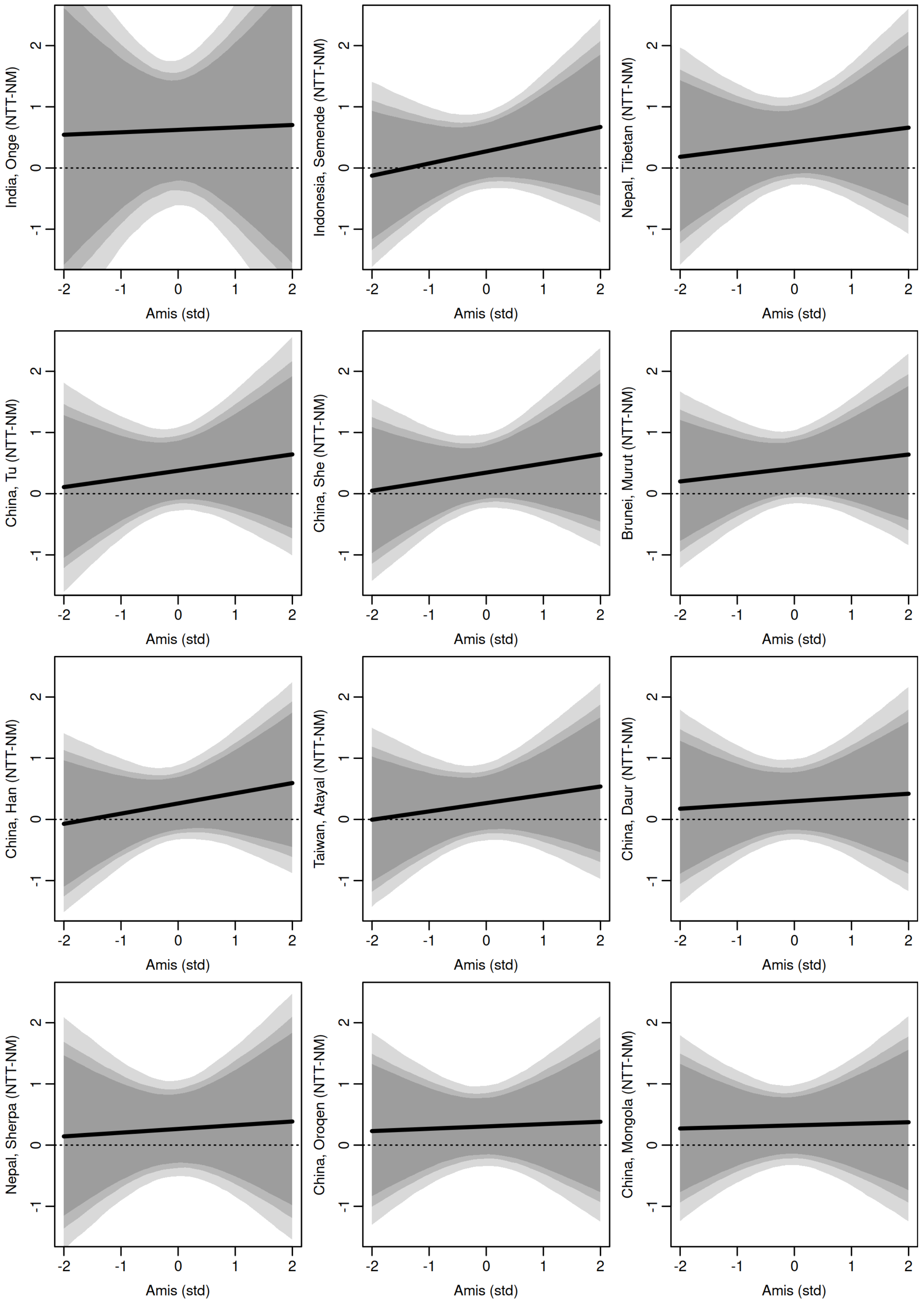
India, Onge (NTT-NM)
Indonesia, Semende (NTT-NM)
Nepal, Tibetan (NTT-NM)
China, Tu (NTT-NM)
China, She (NTT-NM)
Brunei, Murut (NTT-NM)
China, Han (NTT-NM)
Taiwan, Atayal (NTT-NM)
China, Daur (NTT-NM)
Nepal, Sherpa (NTT-NM)
China, Oroqen (NTT-NM)
China, Mongola (NTT-NM)
Amis (std)
-2
-1
0
1
2

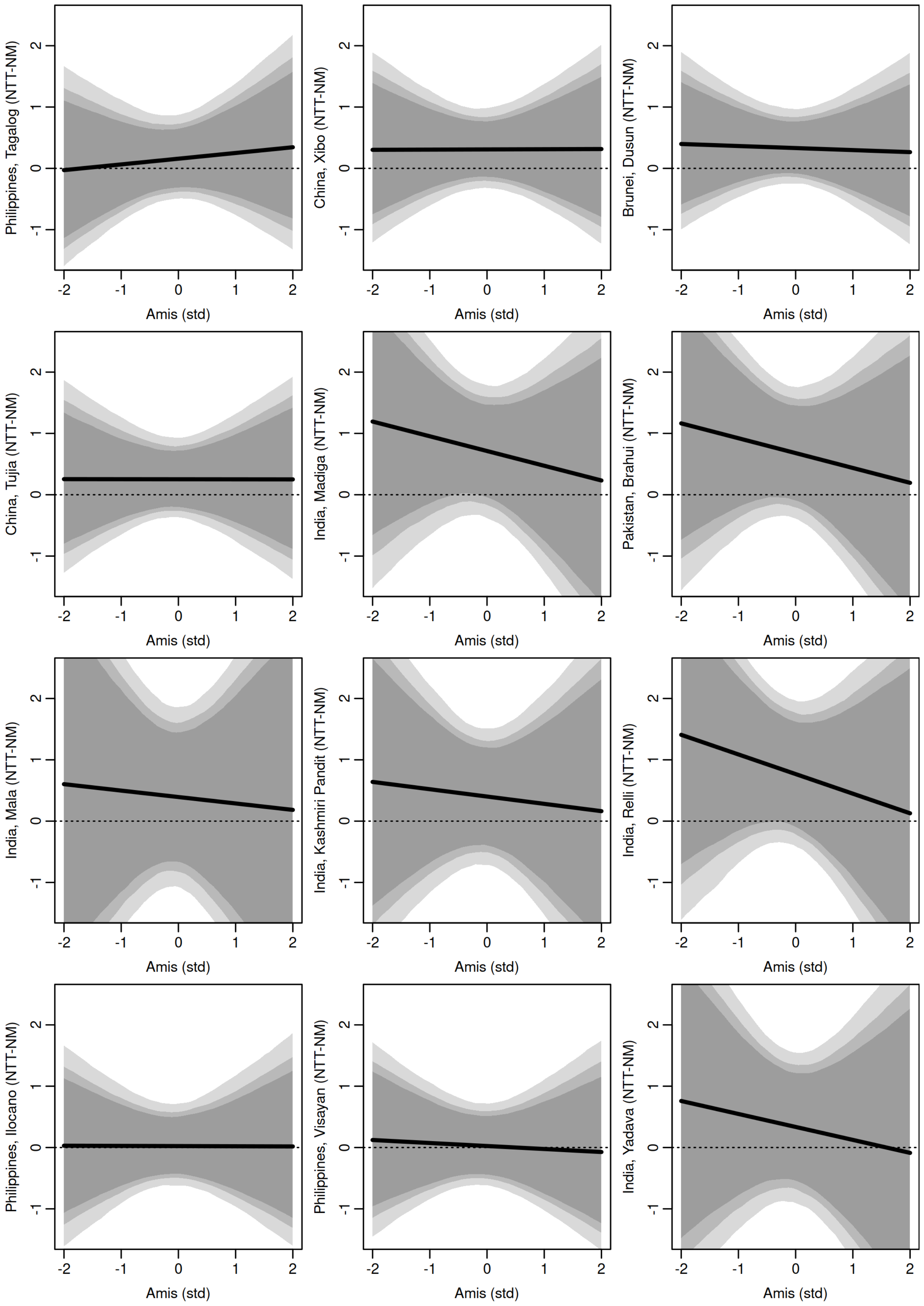
Philippines, Tagalog (NTT-NM)
China, Xibo (NTT-NM)
Brunei, Dusun (NTT-NM)
China, Tujia (NTT-NM)
India, Madiga (NTT-NM)
Pakistan, Brahui (NTT-NM)
India, Mala (NTT-NM)
India, Kashmiri Pandit (NTT-NM)
India, Relli (NTT-NM)
Philippines, Ilocano (NTT-NM)
Philippines, Visayan (NTT-NM)
India, Yadava (NTT-NM)
Amis (std)
-2
-1
0
1
2

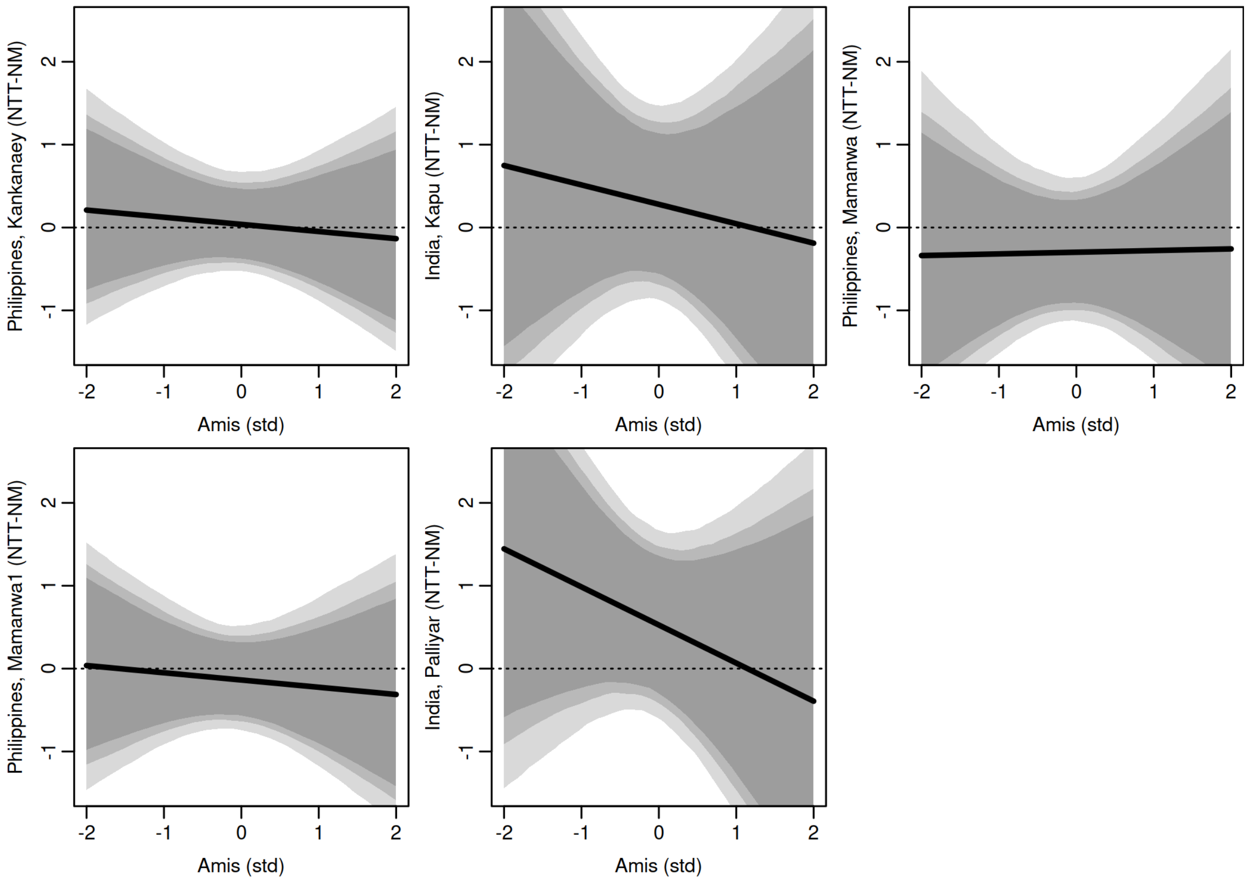

Philippines, Kankanaey (NTT-NM)
India, Kapu (NTT-NM)
Philippines, Mamanwa (NTT-NM)
Philippines, Mamanwa1 (NTT-NM)
India, Palliyar (NTT-NM)
Amis (std)

Supplementary Figure 8 - Differences between the posterior distribution of the regression models for the East Nusa Tenggara and North Moluccas (contrasts). Each plot shows the contrasts for the pair of ancient groups shown in the x-y axis labels. The black line represents differences in the posterior means and the shades show the differences in the credible intervals.

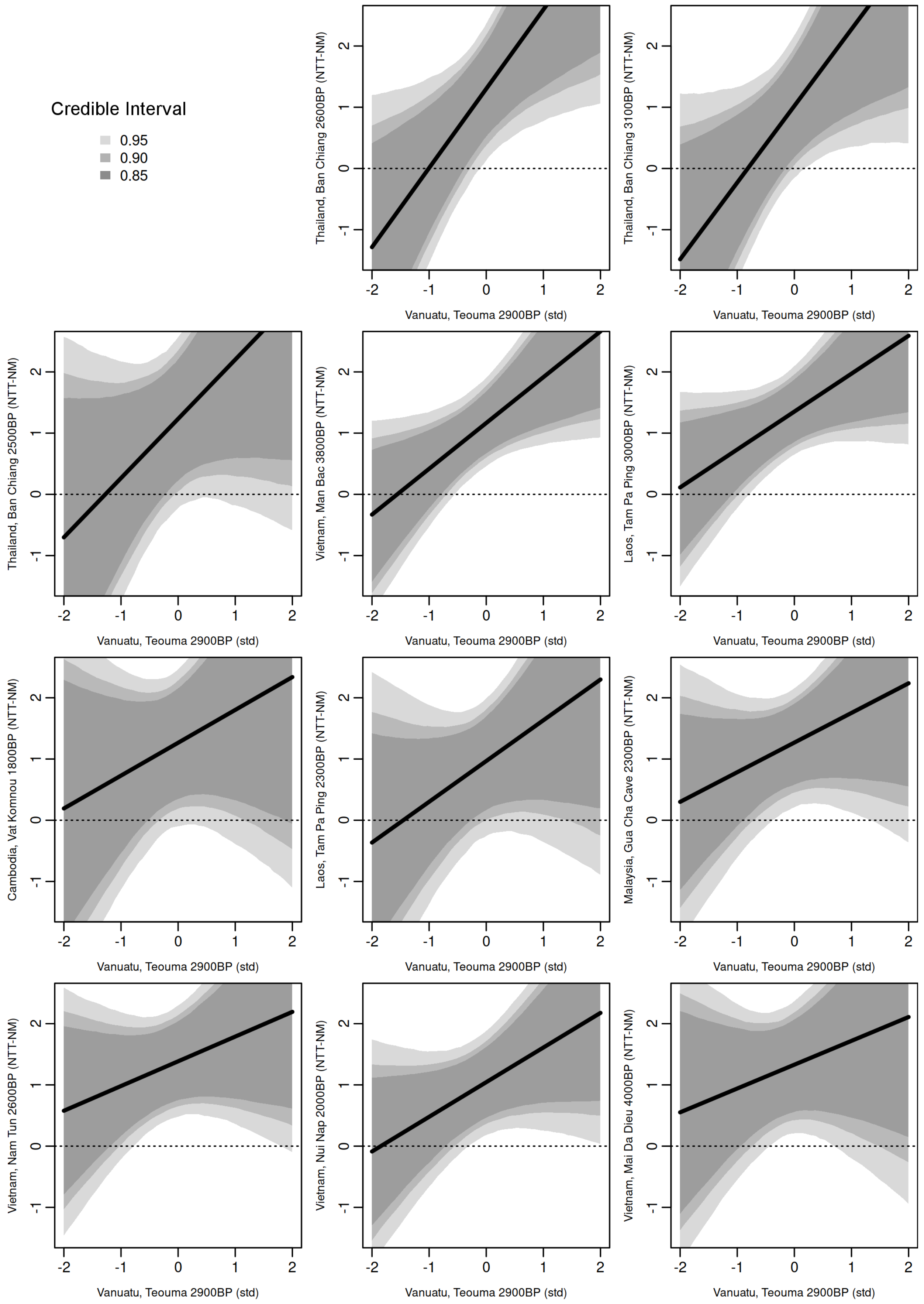

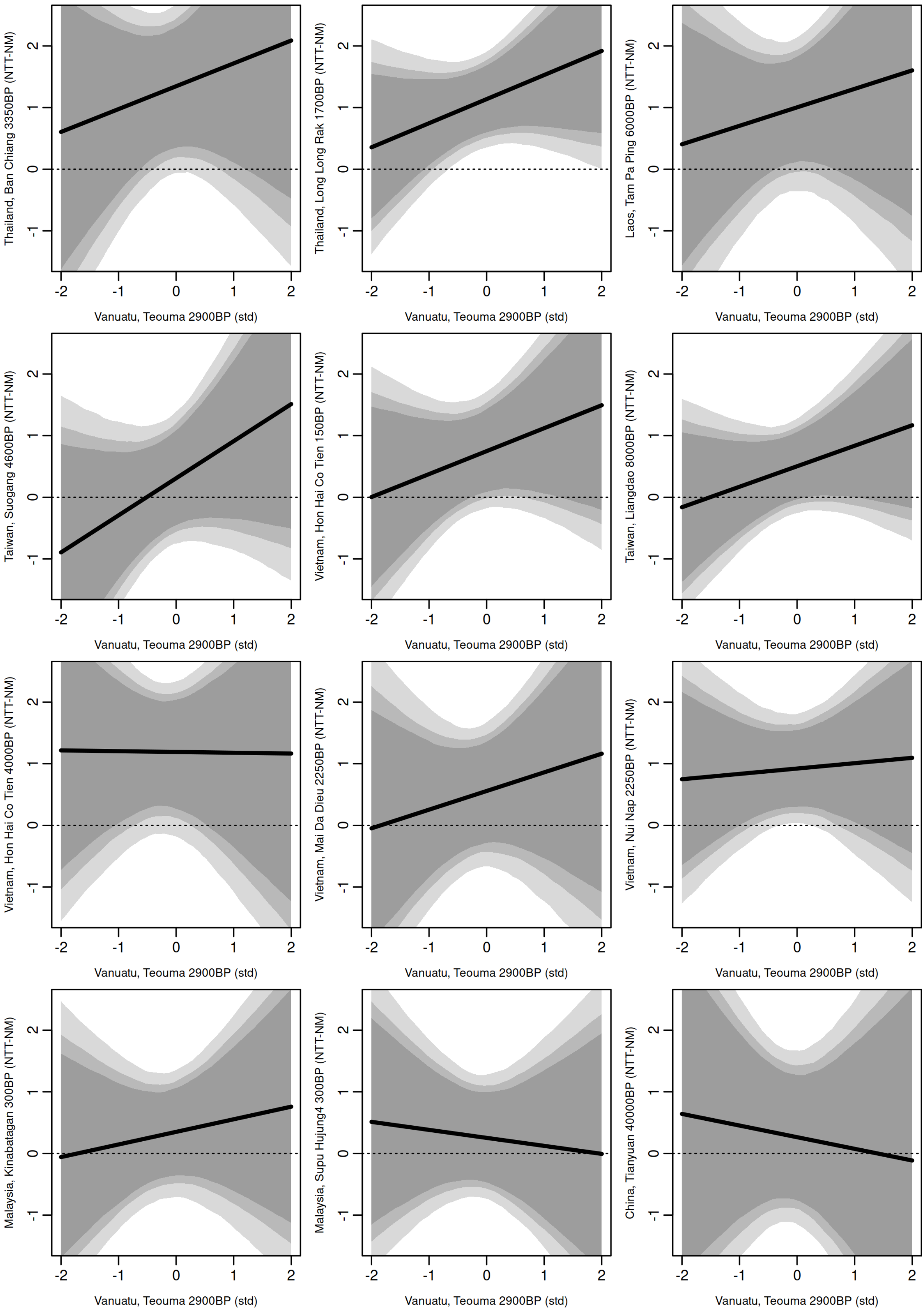
Thailand, Ban Chiang 3350BP (NTT-NM)
Vanuatu, Teouma 2900BP (std)
Thailand, Long Long Rak 1700BP (NTT-NM)
Vanuatu, Teouma 2900BP (std)
Laos, Tam Pa Ping 6000BP (NTT-NM)
Vanuatu, Teouma 2900BP (std)
Taiwan, Suogang 4600BP (NTT-NM)
Vanuatu, Teouma 2900BP (std)
Vietnam, Hon Hai Co Tien 150BP (NTT-NM)
Vanuatu, Teouma 2900BP (std)
Taiwan, Liangdao 8000BP (NTT-NM)
Vanuatu, Teouma 2900BP (std)
Vietnam, Hon Hai Co Tien 4000BP (NTT-NM)
Vanuatu, Teouma 2900BP (std)
Vietnam, Mai Da Dieu 2250BP (NTT-NM)
Vanuatu, Teouma 2900BP (std)
Vietnam, Nui Nap 2250BP (NTT-NM)
Vanuatu, Teouma 2900BP (std)
Malaysia, Kinabatagan 300BP (NTT-NM)
Vanuatu, Teouma 2900BP (std)
Malaysia, Supu Hujung4 300BP (NTT-NM)
Vanuatu, Teouma 2900BP (std)
China, Tianyuan 40000BP (NTT-NM)
Vanuatu, Teouma 2900BP (std)

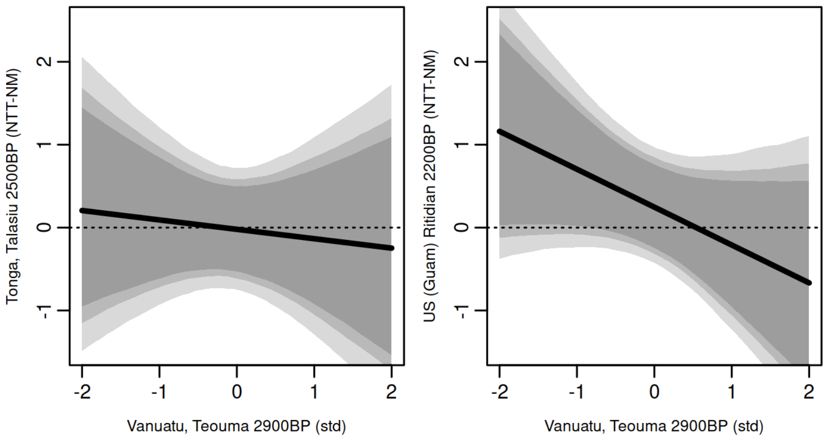
Tonga, Talasiu 2500BP (NTT-NM)
Vanuatu, Teouma 2900BP (std)
US (Guam) Ritidian 2200BP (NTT-NM)
Vanuatu, Teouma 2900BP (std)
-2
-1
0
1
2

Supplementary Figure 9 - f_4-statistic of the form f_4(Mbuti, new ancient Wallacean; New Guinea Highlanders, test) computed for each ancient group from Wallacea separately. The test groups (shown to the right of each value) consist of Australo-Papuans with no discernable Asian ancestry and a recently published pre-Neolithic individual from Sulawesi (Leang Panninge). Data are presented as exact f_4-values ± 2 SE. Values in green are not significantly different from zero (|Z| < 2), whereas values in red are significantly different from zero (|Z| > 2). Significant negative results indicate that the new ancient Wallacean shares additional drift with the New Guinea Highlands, while positive results indicate that the new ancient Wallacean shares additional drift with the test population.

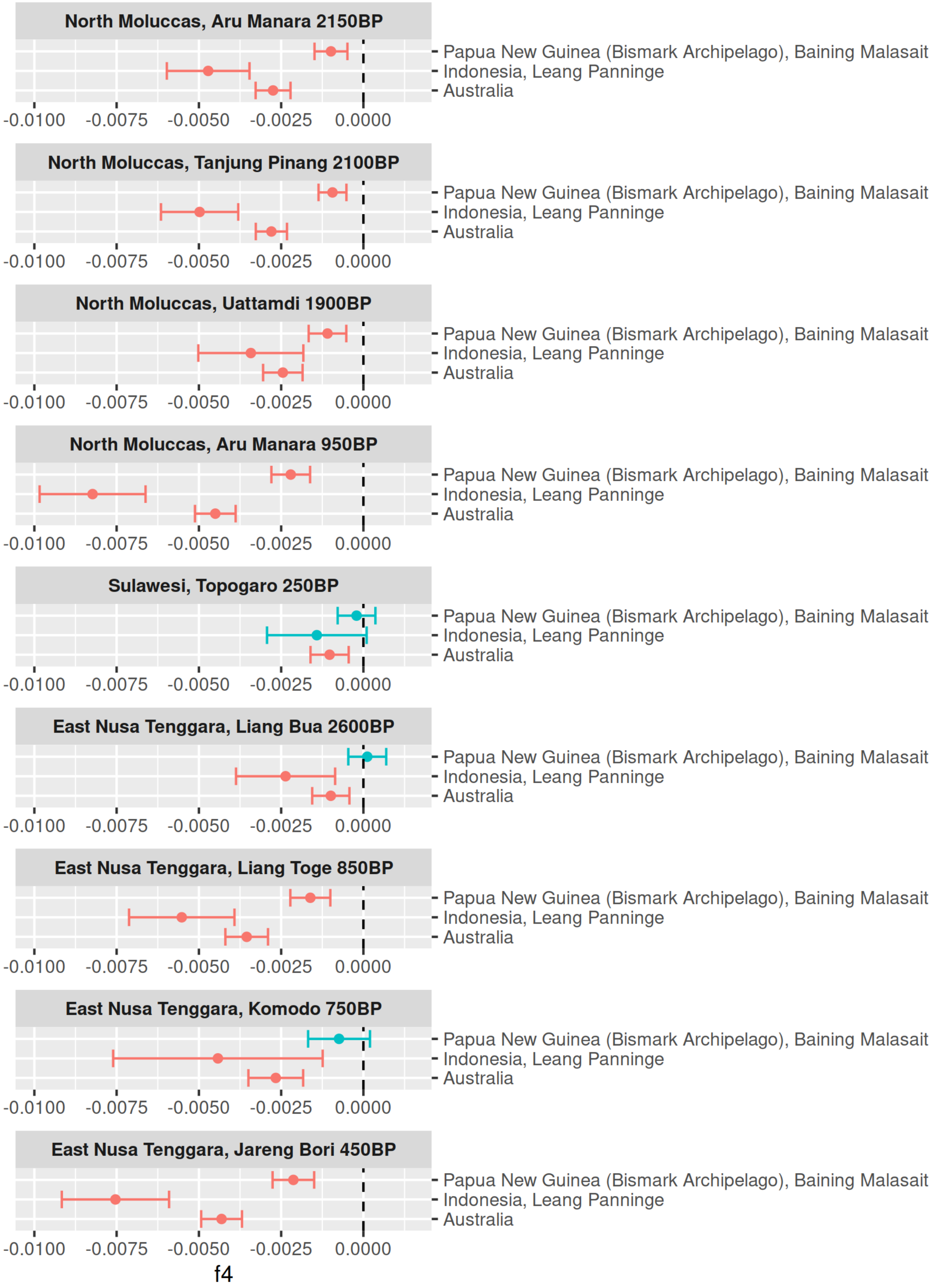

Supplementary Figure 10 – Correlation between the amount of Papuan-related ancestry in ancient Wallacean groups and their shared drift with different Australo-Papuan groups, as well as a pre-Neolithic individual from Sulawesi (Leang Panninge). The x-axis presents the Papuan-related ancestry proportion ± 1 SE calculated with block jackknife in the qpAdm software. The shared drift is presented as exact f_4-values ± 2 SE (y-axis).

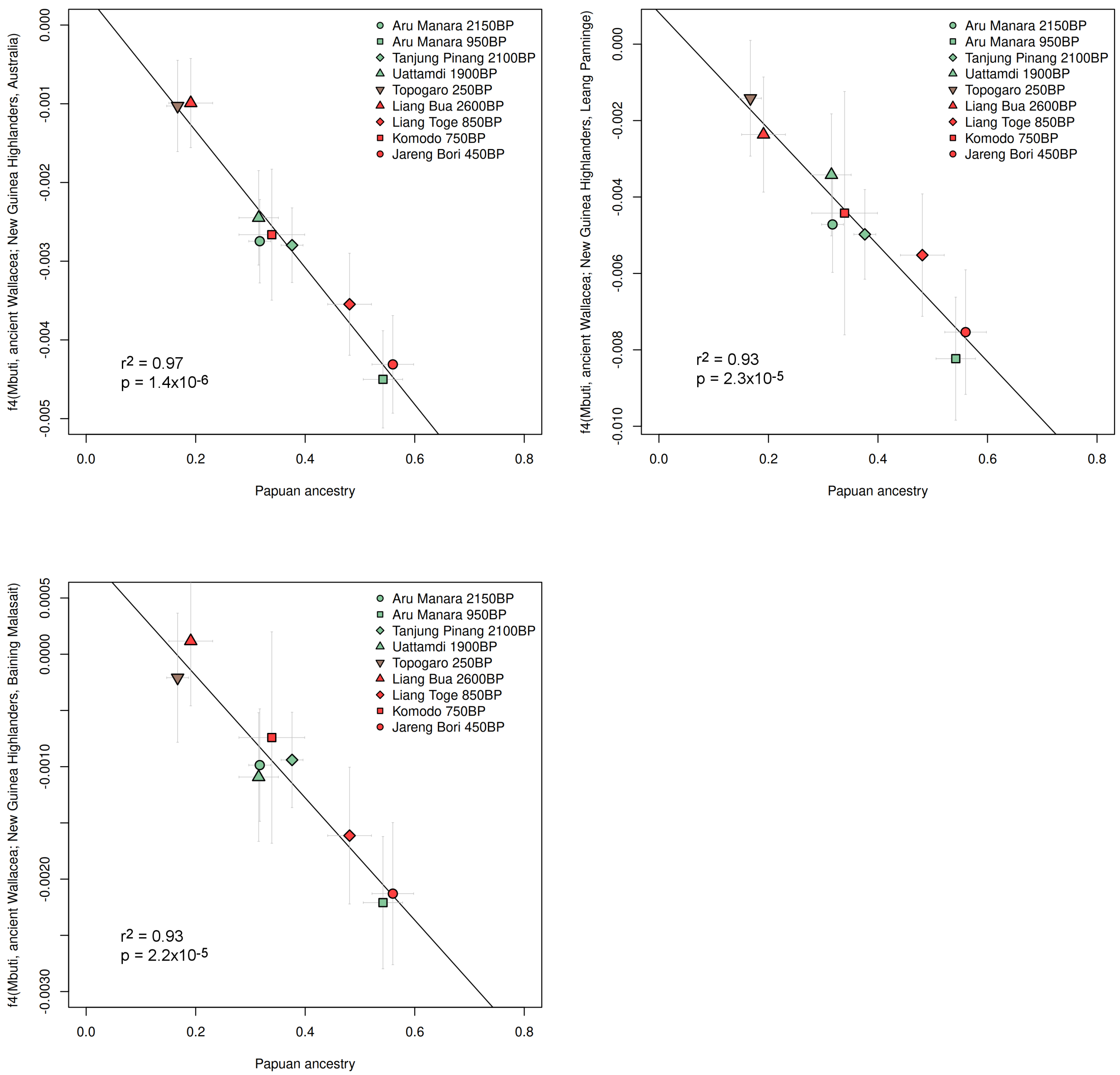

Supplementary Figure 11 – Ancestry covariance in ancient Wallaceans. The number of individuals included in each group is shown in parenthesis, next to the group label. The inferred admixture date (before adding the sample age) ± 2 SE are shown in generations.

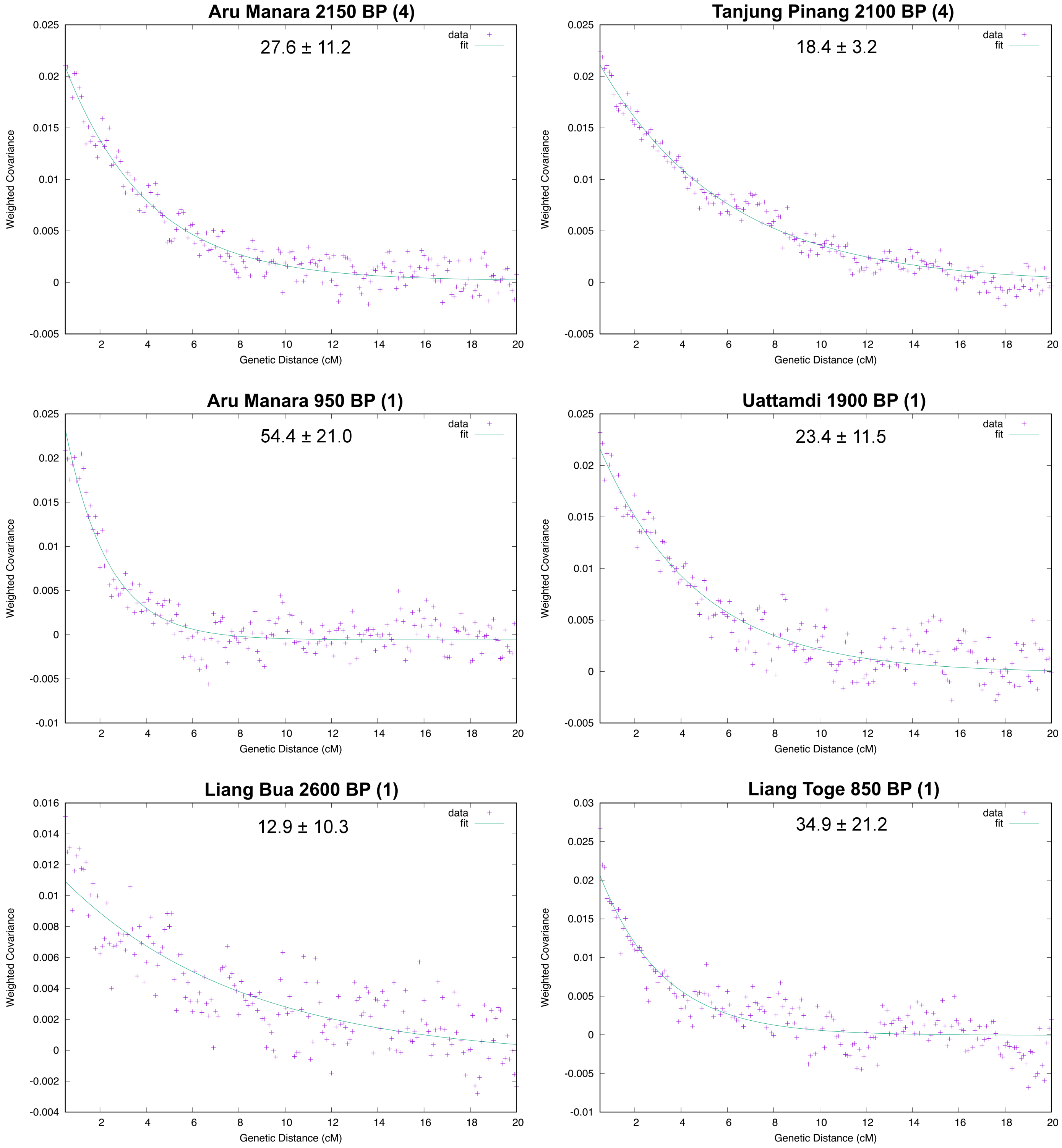

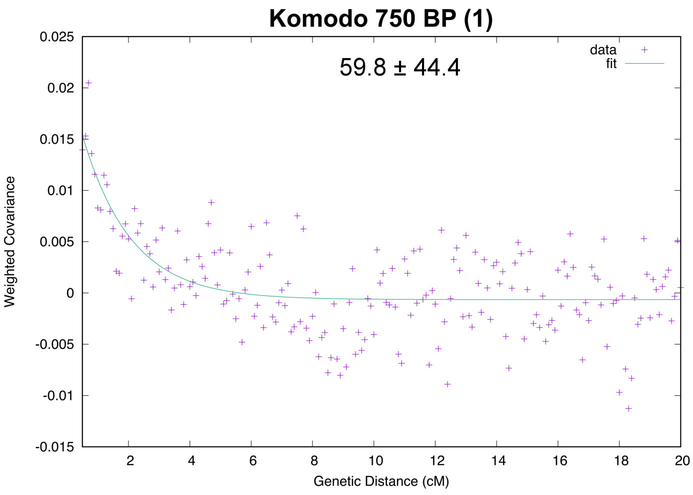
Komodo 750 BP (1)
59.8 ± 44.4
data
fit
Weighted Covariance
Genetic Distance (cM)
0.025
0.02
0.015
0.01
0.005
0
-0.005
-0.01
-0.015
2
4
6
8
10
12
14
16
18
20

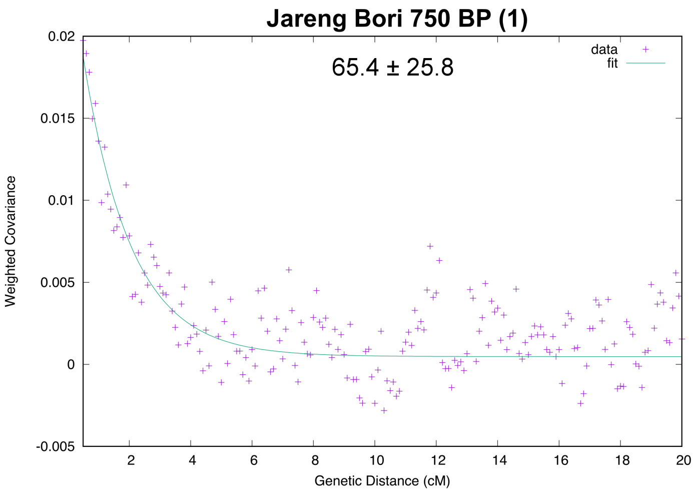
Jareng Bori 750 BP (1)
65.4 ± 25.8
data
fit
Weighted Covariance
Genetic Distance (cM)
0.02
0.015
0.01
0.005
0
-0.005
2
4
6
8
10
12
14
16
18
20

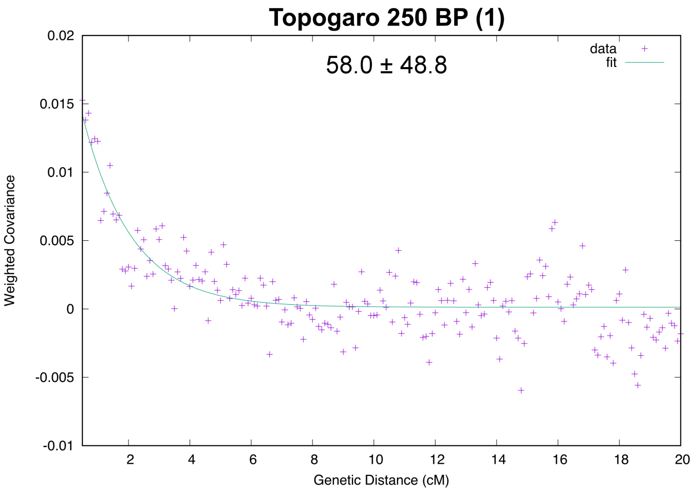
Topogaro 250 BP (1)
58.0 ± 48.8
data
fit
Weighted Covariance
Genetic Distance (cM)
0.02
0.015
0.01
0.005
0
-0.005
-0.01
2
4
6
8
10
12
14
16
18
20
