# Supplementary material for: Ancient genomes from the last three millennia support multiple human dispersals into Wallacea
Source: Nat Ecol Evol. 2022 Jun 9;6(7):1024–34. doi: 10.1038/s41559-022-01775-2 (PMC9262713; doi:10.1038/s41559-022-01775-2)
Supplement: Supplementary file 2 — Reporting Summary [file 41559_2022_1775_MOESM2_ESM.pdf]

nature portfolio

Corresponding author(s): Sandra Oliveira, Johannes Krause, Cosimo Posth, Mark Stoneking

Last updated by author(s): May 17, 2022

Reporting Summary

Nature Portfolio wishes to improve the reproducibility of the work that we publish. This form provides structure for consistency and transparency in reporting. For further information on Nature Portfolio policies, see our Editorial Policies and the Editorial Policy Checklist.

Statistics

For all statistical analyses, confirm that the following items are present in the figure legend, table legend, main text, or Methods section.

n/a	Confirmed	
☐	☒	The exact sample size (*n*) for each experimental group/condition, given as a discrete number and unit of measurement
☐	☒	A statement on whether measurements were taken from distinct samples or whether the same sample was measured repeatedly
☐	☒	The statistical test(s) used AND whether they are one- or two-sided<br>*Only common tests should be described solely by name; describe more complex techniques in the Methods section.*
☐	☒	A description of all covariates tested
☐	☒	A description of any assumptions or corrections, such as tests of normality and adjustment for multiple comparisons
☐	☒	A full description of the statistical parameters including central tendency (e.g. means) or other basic estimates (e.g. regression coefficient) AND variation (e.g. standard deviation) or associated estimates of uncertainty (e.g. confidence intervals)
☐	☒	For null hypothesis testing, the test statistic (e.g. *F*, *t*, *r*) with confidence intervals, effect sizes, degrees of freedom and *P* value noted<br>*Give P values as exact values whenever suitable.*
☐	☒	For Bayesian analysis, information on the choice of priors and Markov chain Monte Carlo settings
☒	☐	For hierarchical and complex designs, identification of the appropriate level for tests and full reporting of outcomes
☐	☒	Estimates of effect sizes (e.g. Cohen's *d*, Pearson's *r*), indicating how they were calculated

Our web collection on statistics for biologists contains articles on many of the points above.

Software and code

Policy information about availability of computer code

Data collection	No software was used for data collection

Data analysis

EAGER v.1.92.55
ClipAndMerge
AdapterRemoval v.2
BWA v.0.7.12
DeDup v.0.12.2
SAMtools v1.3
pileupCaller v.8.6.5
EIGENSOFT v.7.2.1 (mergeit, convertf)
DamageProfiler v.0.3.1
ANGSD v.0.919
contamMix
PLINK v.1.9
smartpca v.10210
DyStruct v.1.1.0
ADMIXTOOLS v.4.1
admixr R package
rethinking R package
DATES v.753
RStudio v.1.1.38
OxCal v.4.4

For manuscripts utilizing custom algorithms or software that are central to the research but not yet described in published literature, software must be made available to editors and reviewers. We strongly encourage code deposition in a community repository (e.g. GitHub). See the Nature Portfolio guidelines for submitting code & software for further information.

Data

Policy information about availability of data

All manuscripts must include a data availability statement. This statement should provide the following information, where applicable:

- Accession codes, unique identifiers, or web links for publicly available datasets
- A description of any restrictions on data availability
- For clinical datasets or third party data, please ensure that the statement adheres to our policy

Alignment files of the nuclear and mitochondrial DNA sequences for the newly sequenced individuals are available at the ENA database under the accession number PRJEB48109.

Field-specific reporting

Please select the one below that is the best fit for your research. If you are not sure, read the appropriate sections before making your selection.

☒ Life sciences ☐ Behavioural & social sciences ☐ Ecological, evolutionary & environmental sciences

For a reference copy of the document with all sections, see nature.com/documents/nr-reporting-summary-flat.pdf

Life sciences study design

All studies must disclose on these points even when the disclosure is negative.

Sample size	Sample size was not predetermined and was based on the availability of skeletal material.
Data exclusions	No data from the newly generated ancient samples were excluded. Related individuals, identified among the previously published datasets used to contextualize genetic variation, were excluded according to a pre-defined criteria outlined in the methods.
Replication	Principal component, DyStruct, qpAdm, Admixture History Graph (AHG), and admixture dating analyses were carried out based on two different datasets that included present-day populations from Island Southeast Asia genotyped on the Affymetrix 6.0 (dataset 1) and the Affymetrix Axiom Genome-Wide Human Array (dataset 2). The findings were consistent for all analyses.
Randomization	We grouped ancient samples from the same archaeological site based on radiocarbon dates or archaeological context when relevant to improve the power of estimations, and grouped present-day samples according to the population of origin. We confirmed that samples from the same site and age have a similar genetic profile in unsupervised analysis (PCA and DyStruct) before grouping them.
Blinding	Blinding was not relevant for this study. The data analysis was performed for all individuals separately or into subgroups defined by external information (archaeological context and dates).

Reporting for specific materials, systems and methods

We require information from authors about some types of materials, experimental systems and methods used in many studies. Here, indicate whether each material, system or method listed is relevant to your study. If you are not sure if a list item applies to your research, read the appropriate section before selecting a response.

Materials & experimental systems

n/a	Involved in the study
☒	☐ Antibodies
☒	☐ Eukaryotic cell lines
☐	☒ Palaeontology and archaeology
☒	☐ Animals and other organisms
☒	☐ Human research participants
☒	☐ Clinical data
☒	☐ Dual use research of concern

Methods

n/a	Involved in the study
☒	☐ ChIP-seq
☒	☐ Flow cytometry
☒	☐ MRI-based neuroimaging

Palaeontology and Archaeology

Specimen provenance

The specimens reported in this study come from eight archaeological sites in Indonesia: Aru Manara, Tanjung Pinang, Gua Uattamdi, Topogaro, Liang Bua, Liang Toge, Komodo, and Jareng Bori. The archaeological context and detailed information on the specimens collection are described in the Supplementary information.

Specimen deposition

The remaining portion of the bones sampled for DNA analysis from the Aru Manara, Topogaro, Tanjung Pinang, and Uattamdi sites will be deposited at the Pusat Penelitian Arkeologi Nasional (Jakarta, Indonesia), and those from Jareng Bori, Komodo, Liang Bua, and Liang Toge will be returned to Prof. Koesbardiati at Universitas Airlangga (Surabaya, Indonesia).

Dating methods

For this study we obtained 13 direct radiocarbon dates. Five radiocarbon dating laboratories were used: Curt-Engelhorn-Zentrum Archäometrie gGmbH in Mannheim (Germany), Radiocarbon Dating Laboratory at the University of Waikato (New Zealand), the University of Oxford's Radiocarbon Accelerator Unit (UK), the Australian National University in Canberra (Australia), and the Iso-trace Research Department of Chemistry, in the University of Otago (New Zealand). Pretreatment processes, quality control protocols, and dating methods used by each laboratory are provided in Supplementary Information and Supplementary Table 12. Conventional radiocarbon ages were calibrated using the OxCal 4.4 program and the INTCAL20 calibration curve, with uncertainties reported at 68% and 95% confidence interval.

☒ Tick this box to confirm that the raw and calibrated dates are available in the paper or in Supplementary Information.

Ethics oversight

For the skeletal material from Tanjung Pinang and Uattamdi, the research was undertaken as part of a collaborative project between Pusat Penelitian Arkeologi Nasional (Jakarta) and the Australian National University, under Lembaga Ilmu Pengetahuan Indonesia Research Permits 6939/S.K./1990, 307/I/KS/1994, and 10290/V3/KS/1995. For the skeletal material from Aru Manara, the research was undertaken as part of a collaborative project between Pusat Penelitian Arkeologi Nasional (Jakarta) and the Tokai University, under Kementarian Riset dan Teknologi Research Permits 0291/SIP/FRP/VIII/2011 and Pusat Penelitian Arkeologi Nasional export permit UM.001/2595/PAN/KPK/IX/2012, 2634/H5/TU/2017. For the skeletal material from Topogaro, the research was undertaken as part of a collaborative project between Pusat Penelitian Arkeologi Nasional (Jakarta) and the Tokai University, under Kementarian Riset dan Teknologi Research Permits 40/EXT/SIP/FRP/SM/VII/2015 and 194/SIP/FRP/E5/Dit.KI/VII/2017 and Pusat Penelitian Arkeologi Nasional export permit 2634/H5/TU/2017. For the skeletal material from Liang Bua, Liang Toge and Komodo, the research was undertaken as part of a collaborative project between Universitas Airlangga (Surabaya, Indonesia) and the Max Planck Institute for the Science of Human History (Jena, Germany) under the Kementerian Ristekdikti Research Permit 303/SIP/FRP/E5/Dit.KI/IX/2017 and Pusat Penelitian Arkeologi Nasional export permit 11404H5/TU/2017. Research of the human remains from Jareng Bori were carried out as part of a collaboration between Australian National University and Universitas Gaja Madja under the Kementerian Ristekdikti Research Permit 1209/FRP/E5/Dit.KI/VI/2016.

Note that full information on the approval of the study protocol must also be provided in the manuscript.
